# Building blocks for recognition-encoded oligoesters that form H-bonded duplexes†
†Electronic supplementary information (ESI) available: Detailed experimental procedures with spectroscopic characterization data, ^19^F NMR titration spectra, binding isotherms, limiting chemical shifts for free and bound states. See DOI: 10.1039/c8sc04896g


**DOI:** 10.1039/c8sc04896g

**Published:** 2019-01-11

**Authors:** Filip T. Szczypiński, Christopher A. Hunter

**Affiliations:** a Department of Chemistry , University of Cambridge , Lensfield Road , Cambridge CB2 1EW , UK . Email: herchelsmith.orgchem@ch.cam.ac.uk

## Abstract

Competition from intramolecular folding is a major challenge in the design of synthetic oligomers that form intermolecular duplexes in a sequence-selective manner. One strategy is to use very rigid backbones that prevent folding, but this design can prejudice duplex formation if the geometry is not exactly right. The alternative approach found in nucleic acids is to use bases (or recognition units) that have different dimensions. A long-short base-pairing scheme makes folding geometrically difficult and is compatible with the flexible backbones that are required to guarantee duplex formation. A monomer building block equipped with a long hydrogen bond donor (phenol, **D**) recognition unit and a monomer building block equipped with a short hydrogen bond acceptor (phosphine oxide, **A**) recognition unit were prepared with differentially protected alcohol and carboxylic acid groups. These compounds were used to synthesise the homo and hetero-sequence 2-mers **AA**, **DD** and **AD**. ^19^F and ^31^P NMR experiments were used to characterize the assembly properties of these compounds in toluene solution. **AA** and **DD** form a stable doubly-hydrogen-bonded duplex with an effective molarity of 20 mM for formation of the second intramolecular hydrogen bond. **AD** forms a duplex of similar stability. There is no evidence of intramolecular folding in the monomeric state of this compound, which shows that the long-short base-pairing scheme is effective. The ester coupling chemistry used here is an attractive method for the synthesis of long oligomers, and the properties of the 2-mers indicate that this molecular architecture should give longer mixed sequence oligomers that show high fidelity sequence-selective duplex formation.

## Abstract 2

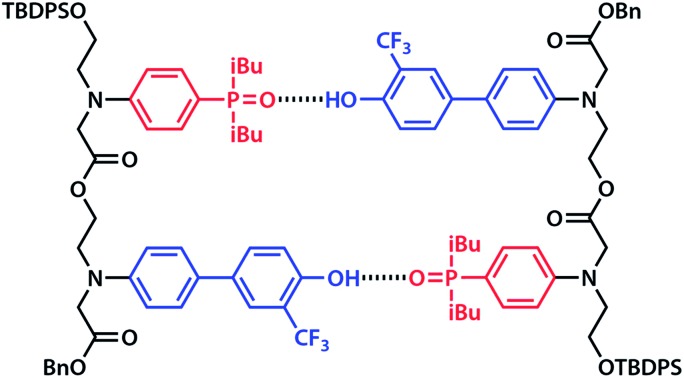
A long-short base-pairing scheme hinders intramolecular folding and allows the use of flexible backbones in duplex-forming oligomers.

## 1 Introduction

### 1.1 Background

Two sequence-complementary strands of nucleic acid will form a stable duplex due to hydrogen bonding interactions between the bases. This supramolecular structure was immediately recognised to provide a plausible mechanism for information transfer between a template strand and a copy in the key biological processes of replication, translation and transcription, where the sequence of the copy is organised by the same base-pairing interactions that lead to duplex formation.^1^,^2^ These copying processes are currently unique to nucleic acids and represent the molecular basis for the evolution of life on this planet. Synthetic systems that form duplexes in the same way are therefore likely to provide a platform for template-directed synthesis of mixed sequence oligomers, and ultimately to the application of directed evolution for the discovery of new functional non-biological molecules.^3^–^8^

It is clear that duplex formation is not restricted to the precise molecular structure found in DNA and RNA. A range of nucleic acid analogues have been prepared in which the phosphate diester,^9^–^11^ the bases,^7^,^12^–^15^ and the sugar have been replaced,^16^–^22^ and all of these oligomers form stable duplexes. Synthetic oligomers that bear no relation to nucleic acids have also been shown to form duplexes through various non-covalent interactions: metal–ligand coordination,^23^,^24^ salt bridges,^25^,^26^ aromatic interactions,^27^ and hydrogen bonding.^28^–^30^ By using two different complementary recognition sites as the equivalent of the nucleic acid bases, it is also possible to encode sequence information into synthetic oligomers, and sequence-selective duplex formation has been demonstrated for short sequences.^26^,^31^

We have been using a single hydrogen bond between a hydrogen bond donor (*e.g.* phenol, **D**) and a hydrogen bond acceptor (*e.g.* phosphine oxide, **A**) as the base-pairing interaction for duplex formation. This two letter alphabet allows information to be encoded in an oligomer as the sequence of **A** and **D** recognition sites. Provided the backbone does not contain any polar functional groups that could compete with the base-pairing interactions, the use of a single hydrogen bond as the base-pair removes any possibility of mismatches, because **A** cannot interact with **A** and **D** cannot interact with **D**. A number of different backbone architectures have been characterized, and the nature of the backbone was found to play a crucial role in the assembly properties of these oligomers.

**Fig. 1:**
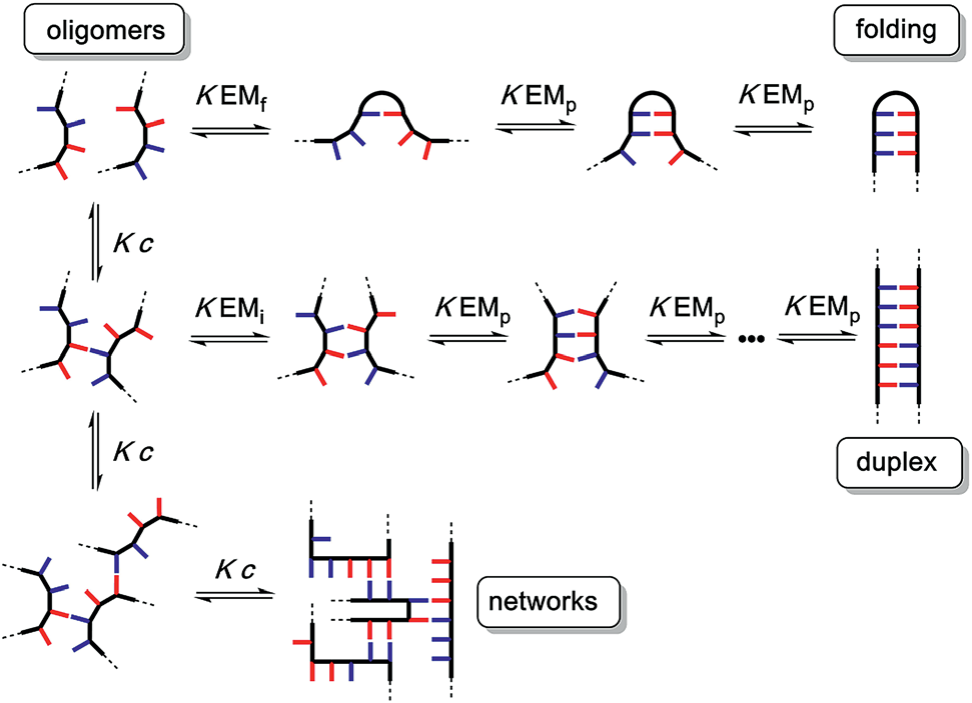
Possible channels for supramolecular assembly of recognition-encoded oligomers. The outcome depends on the concentration, *c*, the association constant for the intermolecular base-pairing interaction, *K*, and the effective molarities for folding, EM_f_, duplex initiation, EM_i_ and duplex propagation, EM_p_.

The different possible self-assembly channels are illustrated in Fig. 1. The key requirement for duplex formation is that the equilibrium constant for propagation of the intramolecular hydrogen bonds that zip up the duplex, *K* EM_p_, is greater than one (*K* is the association constant for formation of an intermolecular hydrogen bond, and EM_p_ is the effective molarity for propagation of intramolecular hydrogen bonds in the duplex).^32^,^33^ One of the competing assembly channels is formation of multiple intermolecular interactions that lead to higher order networks, but this process can be avoided by operating at a concentration, *c*, which is lower than the value of EM_i_, the effective molarity for formation of the first intramolecular hydrogen bond that initiates duplex formation. The other major competing assembly channel is due to the formation an intramolecular hydrogen bond within an oligomer, which leads to folding. The probability of this process is determined by the equilibrium constant *K* EM_f_, where EM_f_ is the effective molarity for folding.

**Fig. 2:**
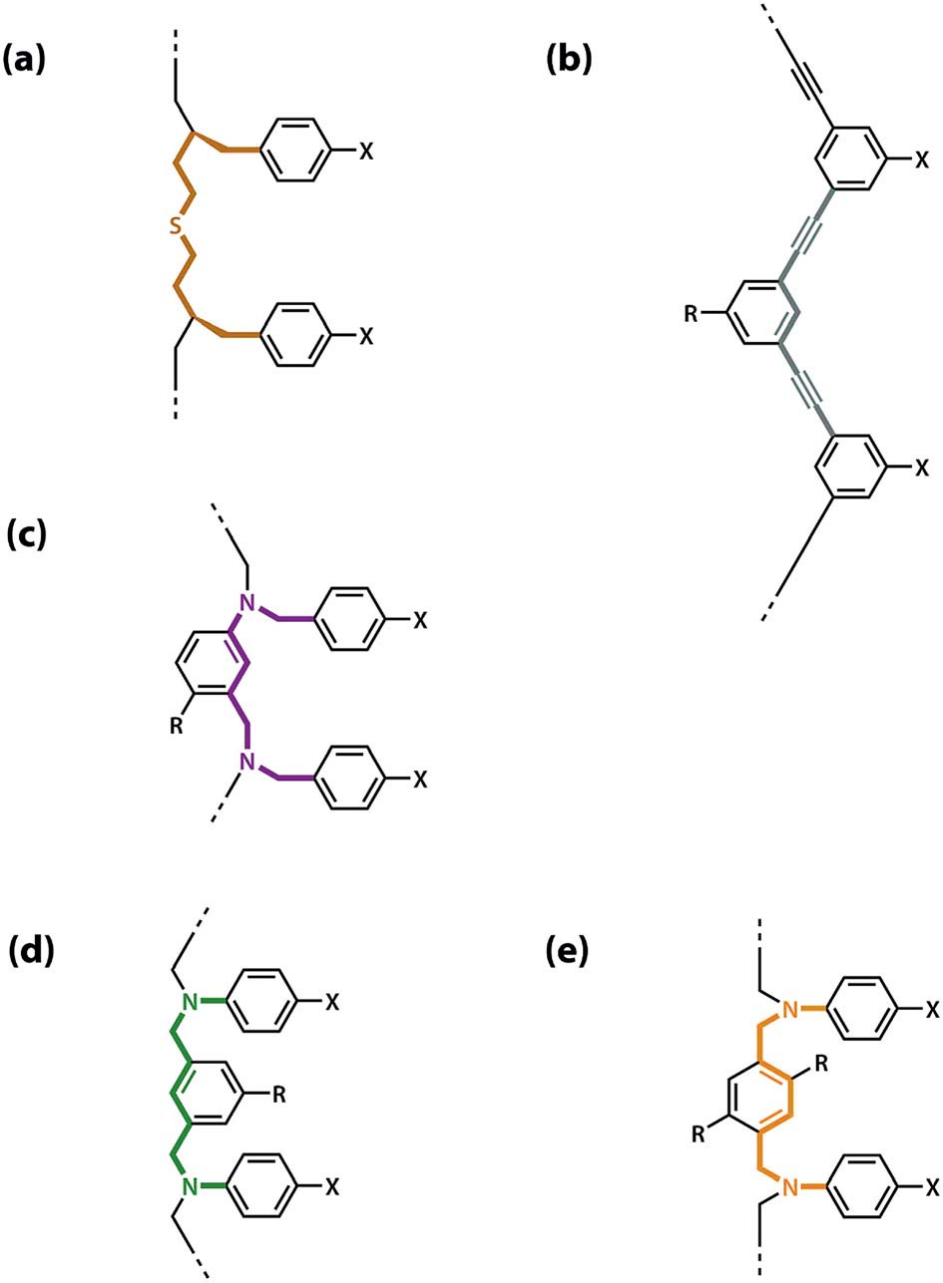
Backbones (a)–(e) of the previously reported synthetic information molecules.^34^–^36^,^38^

The values of the three effective molarity parameters depend on the conformational properties of the backbone. For the very flexible backbone shown in Fig. 2(a), the values of EM_i_ and EM_p_ are 10 mM to 30 mM, and the duplex channel dominates for length complementary homo-oligomers.^34^ For the very rigid backbone shown in Fig. 2(b), similar results were obtained with EM_i_ and EM_p_ values of 40 mM to 70 mM.^35^ Geometry is critical for more rigid backbones. The backbone shown in Fig. 2(b) has a well-defined geometry, which places the recognition groups in the correct orientation for duplex formation. However, for backbones of intermediate rigidity, where the conformational properties are more difficult to predict, mixed results were obtained. The backbone shown in Fig. 2(c) formed duplexes with EM_i_ = EM_p_ = 10 mM,^36^ but the backbones shown in Fig. 2(d) and (e) did not lead to extended duplexes. For these two systems, EM_i_ was similar to the values found for the other backbones (10 mM to 20 mM), but the geometry was not compatible with duplex propagation, and EM_p_ was too low to measure.^37^

The results obtained for homo-oligomers suggest that highly flexible backbones should provide a reliable platform for the design of duplex-forming oligomers. Conformational flexibility ensures that the backbone will always be able to adapt to a geometry compatible with base-pair formation in an extended duplex. More rigid backbones are difficult to design with the degree of accuracy required to guarantee the geometric complementarity needed for formation of an extended duplex.^37^ The values of effective molarity measured for the very flexible backbone and the very rigid backbone shown in Fig. 2 are similar, so it appears that effective molarities associated with duplex formation are not adversely affected by conformational flexibility. Very flexible backbones are easily accessed, so this approach would make backbone design straightforward.

**Fig. 3:**
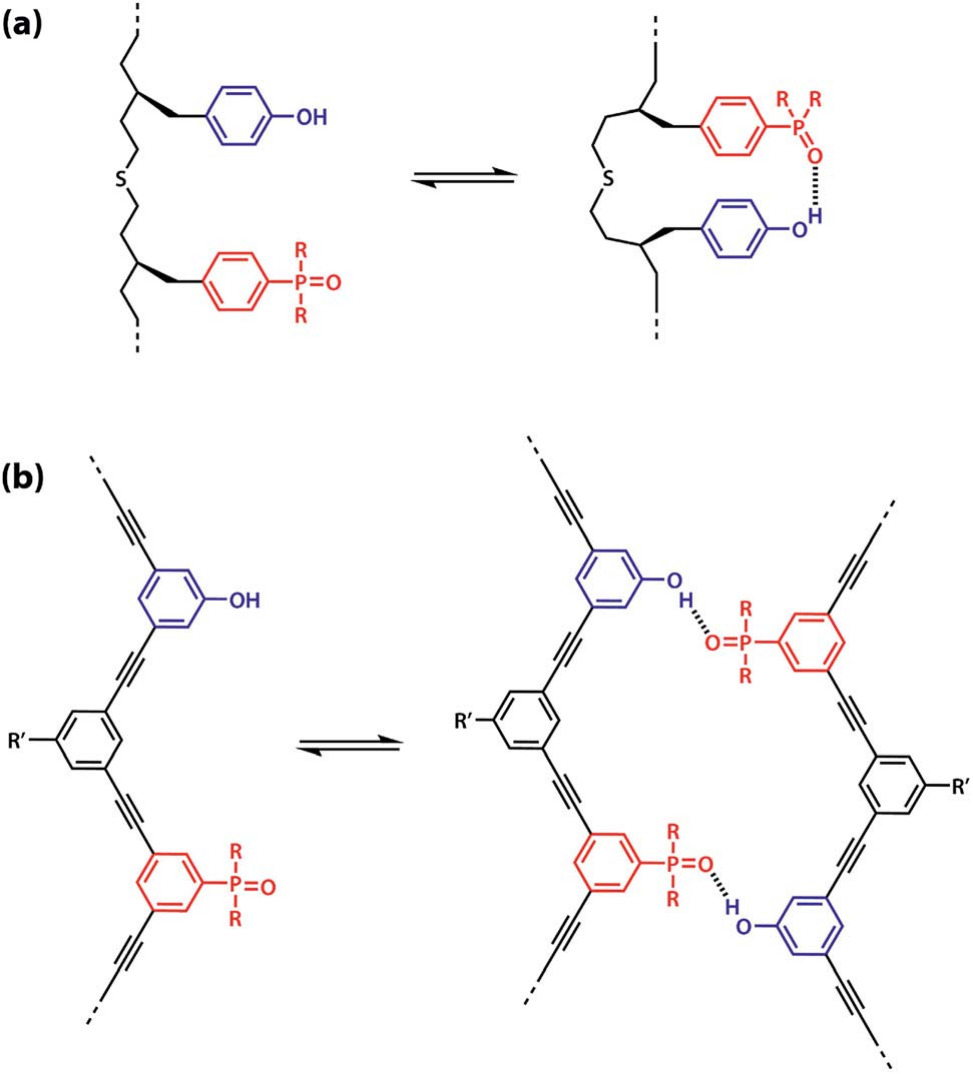
(a) Intramolecular 1,2-folding in information molecules with flexible backbones. (b) Duplex formation in information molecules with rigid backbones.

However, the effective molarity for intramolecular folding, EM_f_, also depends on the conformational properties of the backbone. As shown in Fig. 3(a), a long flexible backbone promotes 1,2-folding between **A** and **D** recognition units that are adjacent in sequence. The value of EM_f_ for this system is about 10 mM, which is comparable to the values of effective molarity for zipping up the duplex, so the folding channel will dominate for mixed sequence oligomers of this architecture.^39^ Of course, longer mixed sequence oligomers will always be able to fold, no matter what backbone is used, and indeed sequence-encoded folding of single-stranded RNA is key to the biological properties.^2^ Folded nucleic acid structures involve looped out bases, so if a single-stranded nucleic acid is annealed with a sequence-complementary strand, duplex formation will dominate, because additional base-pairing interactions are made in the duplex. However, Fig. 1 shows that if 1,2-folding is possible, the number of base-pairs formed in the folding and duplex channels can be identical, so the folding channel will dominate. Minimising 1,2-folding is therefore critical to the design of recognition-encoded oligomers that form sequence-selective duplexes with high fidelity.

**Fig. 4:**
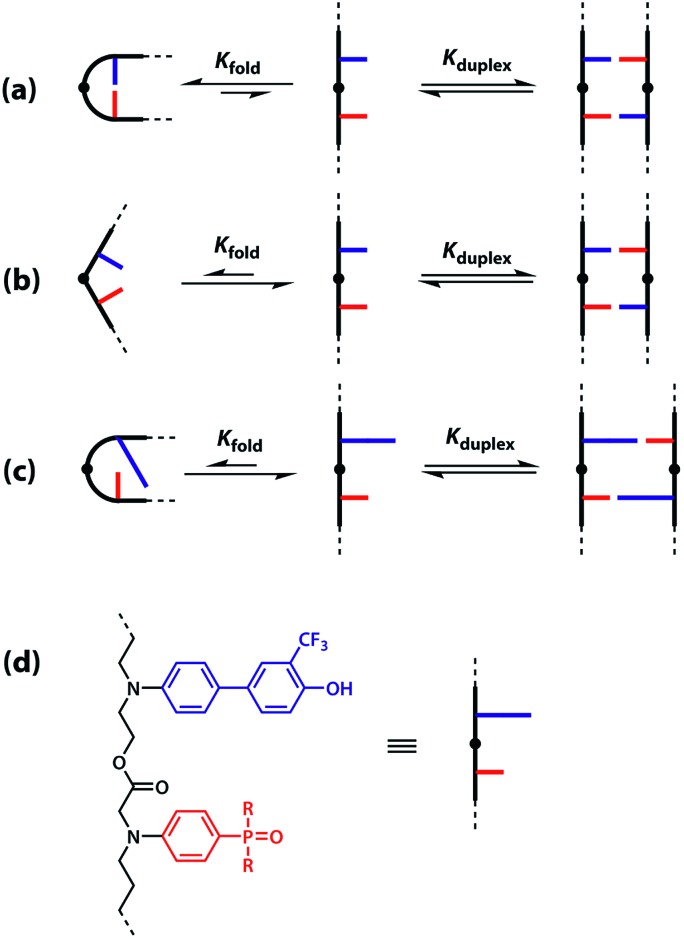
(a) Flexible backbones lead to 1,2-folding; (b) rigid backbones prevent 1,2-folding; (c) recognition units of different dimensions prevent 1,2-folding; (d) molecular design corresponding to the short-long base-pairing scheme.

One strategy for avoiding 1,2-folding is to reduce the value of EM_f_ by increasing the rigidity of the backbone. As shown in Fig. 3(b), the very rigid backbone that we studied previously does not fold, so duplex formation is the dominant assembly channel for mixed sequence oligomers of this architecture. However, it would be preferable to work with more flexible backbones to guarantee duplex formation, as explained above. Here, we explore an alternative strategy for preventing 1,2-folding in oligomers with a very flexible backbone. If two short bases are attached to a long flexible backbone, 1,2-folding is favoured (Fig. 4(a)). Fig. 4(b) illustrates how folding can be prevented by attaching the two short bases to a rigid backbone. Fig. 4(c) shows how changing the dimensions of the bases can be used to prevent folding. By making one of the bases longer than the other, the probability of finding a backbone conformation compatible with folding is significantly reduced, and the duplex assembly channel should dominate. Fig. 4(d) shows the corresponding molecular design that we validate in this paper. It is worth noting that this short-long base-pairing scheme has similar geometrical properties to the purine–pyrimidine base-pairing system found in nucleic acids.

The backbone proposed in Fig. 4(d) uses ester linkages as the coupling chemistry for the synthesis of oligomers. Esters are sufficiently weak hydrogen bond acceptors (*β* ≈ 5.5) not to compete significantly with the phosphine oxide recognition units (*β* ≈ 10.5).^40^ Ester coupling is sufficiently high-yielding to be used for the synthesis of polymers, and iterative coupling could be automated in a peptide synthesiser.^41^–^43^ Orthogonal protecting groups have been developed for the preparation of oligoesters with sequences of different building blocks.^44^–^51^ Here, we describe synthesis of the required monomer building blocks, demonstrate their use in the synthesis of different 2-mer sequences, and show that the long-short base-pairing scheme successfully prevents 1,2-folding for this oligomer architecture.

## 2 Results and discussion

### 2.1 Synthesis

**Scheme 1:**
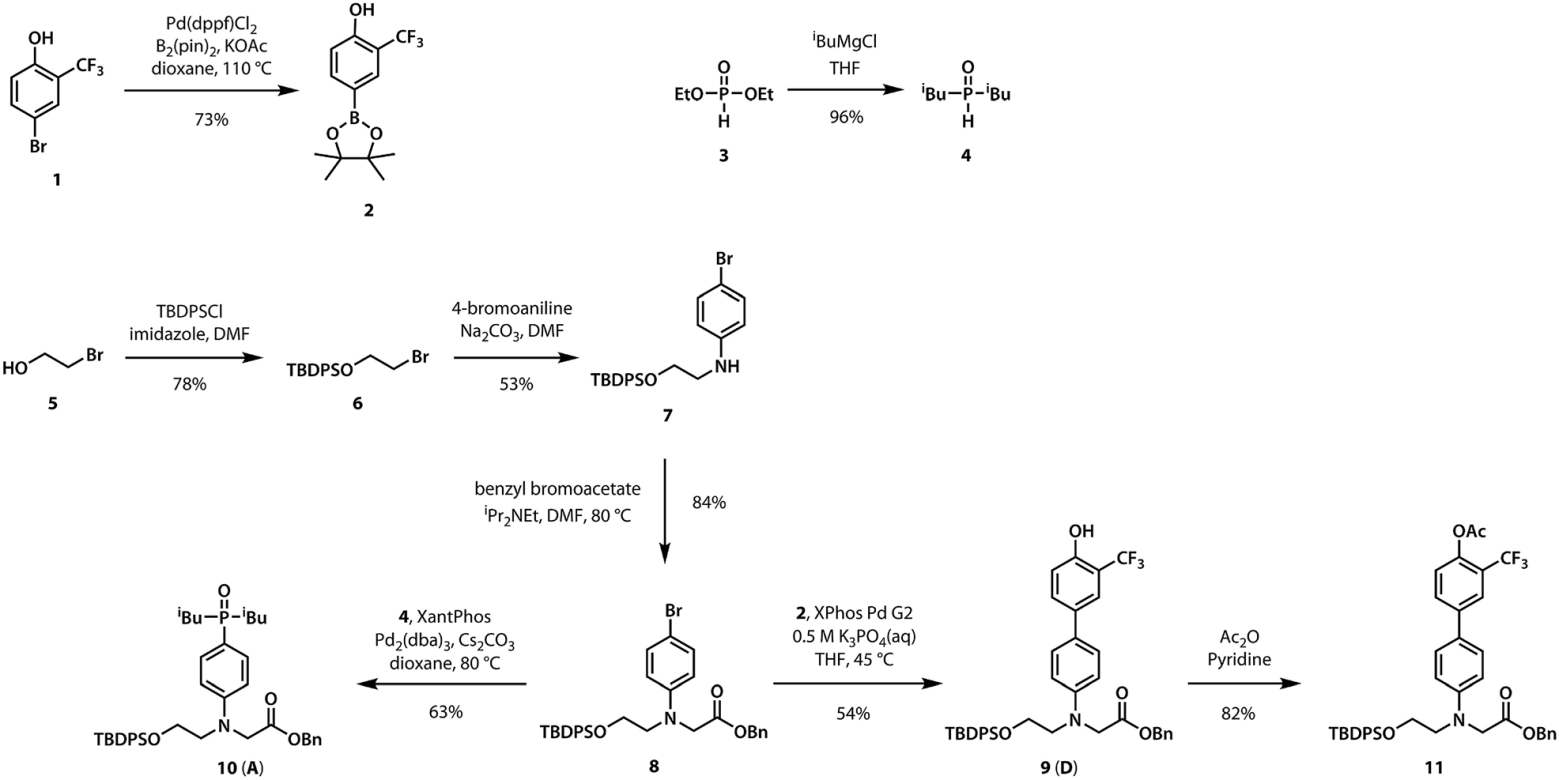
Synthesis of the orthogonally-protected monomers for information oligoesters.

A divergent approach to the synthesis of the monomer building blocks was employed, in which a common aromatic bromide intermediate was coupled with the hydrogen bond donor and acceptor recognition units, as shown in Scheme 1. Commercially available 2-bromoethanol **5** was protected as the silyl ether **6**, which was then used for alkylation of 4-bromoaniline to yield **7**. Aniline **7** was alkylated with benzyl bromoacetate to give the key intermediate **8**. Commercially available phenol **1** was converted to the boronic ester **2**, which was coupled with **8** under Suzuki–Miyaura conditions to give the hydrogen bond donor monomer **9** (**D**). Treatment of commercially available diethyl phosphite **3** with iso-butylmagnesium chloride gave **4**, which was coupled with **8** using palladium(0) and XantPhos to yield the hydrogen bond acceptor monomer **10** (**A**).

**Scheme 2:**
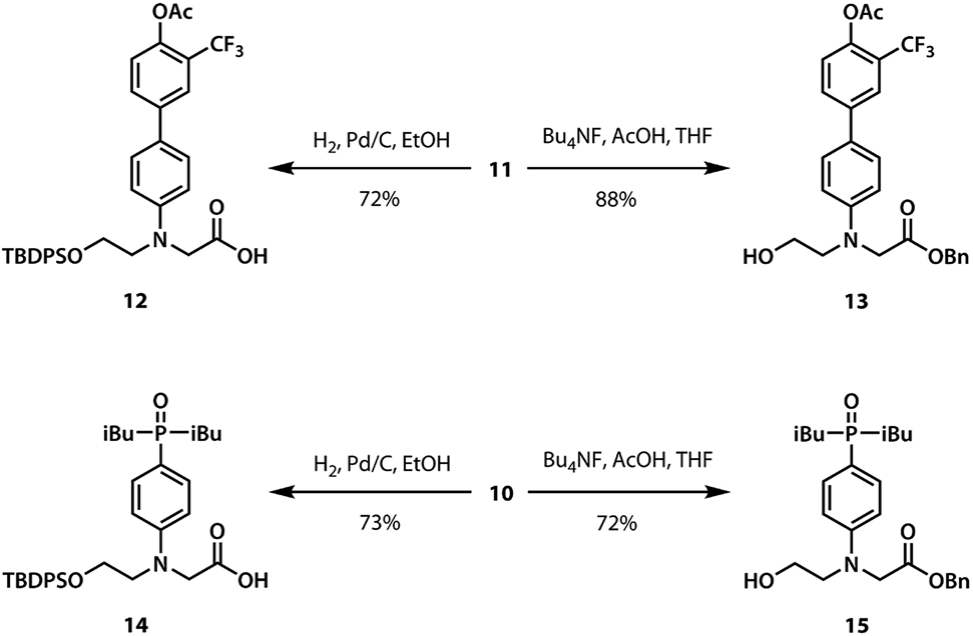
Synthesis of the monoprotected hydroxyacid monomers.

**Scheme 3:**
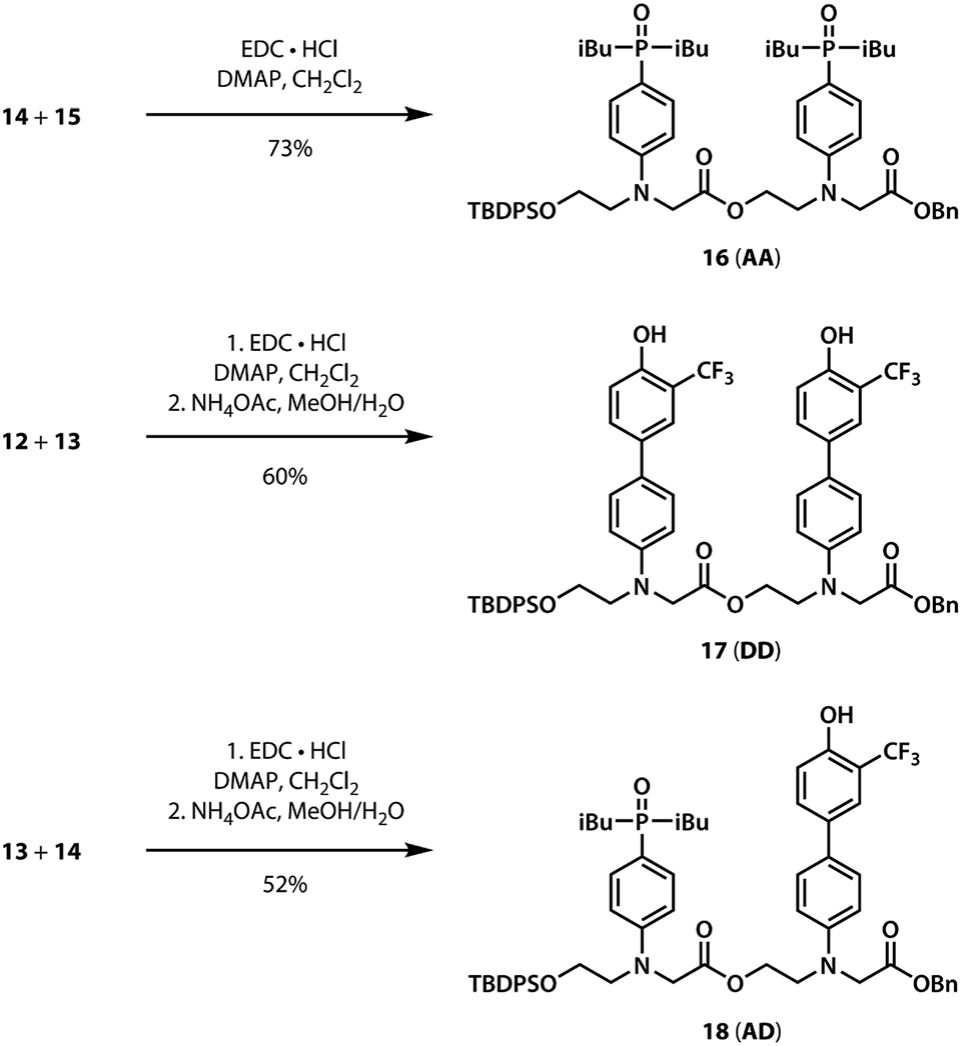
Synthesis of the **AA**, **DD**, and **AD** 2-mers.

For the ester coupling reactions, the potentially reactive phenol moiety in **9** was first protected as the acetyl ester **11** (Scheme 2). The benzyl and TBDPS protecting groups in **10** and **11** were removed orthogonally to give the four precursors **12–15** required for ester coupling reactions. Treatment with hydrogen gas over palladium on charcoal gave the monoprotected carboxylic acids **12** and **14**. Alternatively, reaction with *n*-tetrabutylammonium fluoride buffered with acetic acid gave the monoprotected alcohols **13** and **15**. These monoprotected hydroxyacid monomers were used to synthesise three different 2-mer sequences by EDC coupling with a catalytic amount of *N*,*N*-dimethylaminopyridine (Scheme 3). Coupling **14** with **15** gave **AA** directly. **AD** and **DD** were obtained with the phenol groups protected as acetate esters, but these groups were removed quantitatively by stirring in a solution of ammonium acetate in water and methanol.

### 2.2 NMR binding studies

**Fig. 5:**
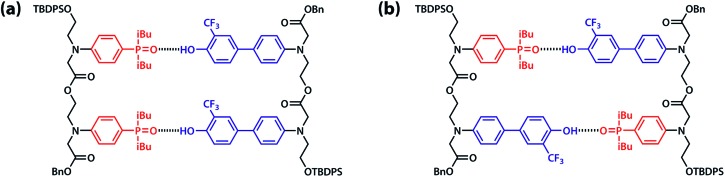
Hydrogen bonded duplexes formed by (a) the **AA** and **DD** 2-mers, and (b) the self-complementary **AD** 2-mer.

Duplex formation and folding were investigated using ^19^F and ^31^P NMR titrations and dilutions in toluene-d_8_ at 298 K. The association constant for formation of the **A·D** complex, which makes a single intermolecular hydrogen bond, was measured by titrating **A** into **D**. A large upfield change in the ^19^F NMR chemical shift of **D** was observed, and the data fit well to a 1 : 1 binding isotherm to give an association constant of *K***_A D_** = 3.8 × 10^3^ M^–1^ (Table 1). The association constant for the **AA·DD** complex was similarly measured by titrating **AA** into **DD**, and the association constant for dimerization of **AD** was determined by a ^19^F NMR dilution experiment in toluene-d_8_ at 298 K. The association constants for the **AA·DD** and **AD·AD** complexes are both two orders of magnitude higher than that for **A·D**, which indicates that there are two cooperative hydrogen bonding interactions in the complexes formed by the sequence complementary 2-mers (Table 1). The limiting ^19^F and ^31^P NMR chemical shifts of the free species (*δ*_free_) and fully bound complexes (*δ*_bound_) were determined by extrapolation of the binding isotherms (Table 1). The values are similar for all three complexes. The large upfield limiting complexation-induced changes in ^19^F NMR chemical shift (0.4 ppm) indicate that all of the phenol groups form hydrogen bonds in all of the complexes. The large downfield limiting complexation-induced changes in ^31^P NMR chemical shift (5–7 ppm) indicate that all of the phosphine oxide groups form hydrogen bonds in all of the complexes. Comparison of the values of the free ^19^F and ^31^P NMR chemical shifts of **AA**, **DD**, and **AD** show that there is no significant intramolecular hydrogen bonding in **AD**, *i.e.* there is no folding in the monomeric state. The results indicate that both the **AA·DD** and **AD·AD** duplexes are fully assembled through the intended base-pairing interactions at mM concentrations in toluene solution at room temperature as shown in Fig. 5.

**Table 1:** Association constants (*K*), effective molarities (EM), limiting NMR chemical shifts (*δ*_free_ and *δ*_bound_), and complexation-induced changes in the chemical shifts (Δ*δ*) for the formation of duplexes in toluene at 298 K

Complex	log *K*/M^–1^	^19^F NMR			^31^P NMR		
		*δ*_free_/ppm	*δ*_bound_/ppm	Δ*δ*/ppm	*δ*_free_/ppm	*δ*_bound_/ppm	Δ*δ*/ppm
**A·D**	3.6 ± 0.1	–61.2	–61.6	–0.4	34.2	41.0	6.8
**AA·DD**	5.8 ± 0.1	–61.1	–61.5	–0.4	34.3	39.3	5.0
**AD·AD**	5.2 ± 0.1	–61.1	–61.5	–0.4	35.8	40.9	5.1

**Fig. 6:**
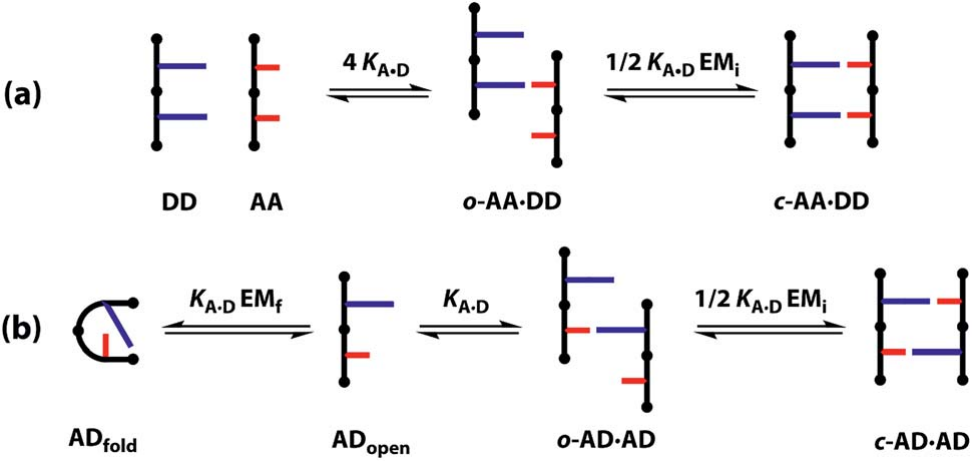
Competing equilibria in the assembly of (a) the **AA·DD** duplex and (b) the **AD·AD** duplex.

A schematic representation of the equilibria involved in duplex assembly is shown in Fig. 6. For **AA·DD**, formation of the first intermolecular hydrogen bond gives an open complex, and formation of the second intramolecular hydrogen bond gives the closed duplex. Assuming that all of the hydrogen bonds in the systems described here are of similar strength, it is possible to describe the association constant for formation of the closed ***c*-AA·DD** duplex in terms of the association constant for formation of a single intermolecular hydrogen bond *K***_A·D_** and the effective molarity for the intramolecular interaction EM_i_. The backbone in these systems has a direction, because the hydroxyl and acid ends are different, so parallel and anti-parallel orientations of the duplex are possible. As the end groups are spatially separated from the recognition sites, we assume that the two possible ***c*-AA·DD** have similar stability. Therefore, the open complex ***o*-AA·DD** has four equally populated states and the closed duplex ***c*-AA·DD** has two.

It is possible to express the association constants for duplex formation in terms of *K***_A·D_** and EM_i_:1
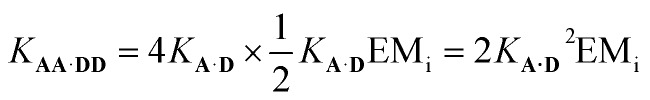



Hence the effective molarity for duplex formation can be determined as:2
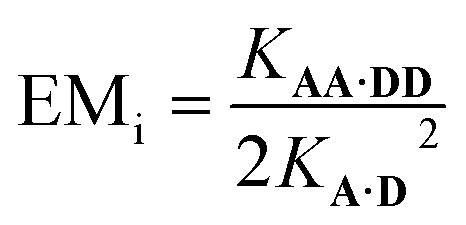



The association constants in Table 1 were used to calculate EM_i_ for this system as 19 ± 3 mM, which is consistent with values of supramolecular effective molarities we have measured for other hydrogen bonded duplexes.^31^,^34^–^36^,^38^,^39^ The equilibrium constant for closing the duplex is given by 
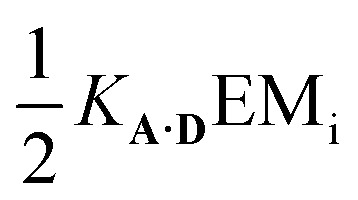
 and is 40 for this system, which implies that the duplex is fully closed and only 2% of the species populate the partially-bound open state ***o*-AD·AD**.

For the closed hetero-2-mer duplex ***c*-AD·AD**, there is no degeneracy associated with the backbone directionality, because the anti-parallel orientation is determined by the sequence. However, there is the possibility of intramolecular 1,2-folding in the monomeric state, which is governed by the corresponding effective molarity EM_f_. Hence, the observed dimerisation constant *K***_AD·AD_** depends on the concentrations of the folded (**AD**_folded_) and open (**AD**_open_) species that are populated in the monomeric state:

3 [**AD**] = [**AD**_open_] + [**AD**_fold_] = [**AD**_open_](1 + *K*_**A·D**_EM_f_)

4
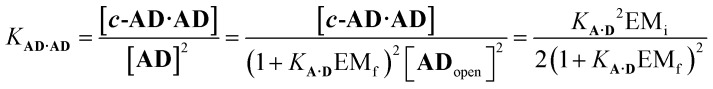



Assuming that the effective molarity for duplex formation, EM_i_, is the same for **AA·DD** and **AD·AD**, it is possible to combine eqn (2) and (4) to determine (*K***_A·D_**EM_f_ + 1), which is the factor that describes the fraction of monomeric **AD** that exists in the folded state:5
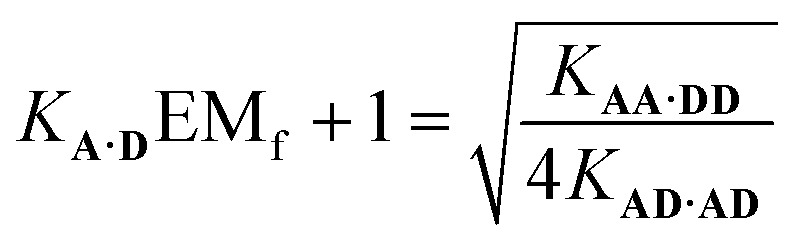



Substituting the values from Table 1 into eqn (5) gives a value of 1.0 for (*K***_A·D_**EM_f_ + 1), which is consistent with the NMR chemical shift data. These results indicate that virtually all monomeric **AD** exists in the open state and the 1,2-folding does not compete with duplex formation in this system.

If the two arrangements of the ***c*-AA·DD** were not degenerate, the statistical factor in eqn (1) would be equal to one, giving (*K***_A·D_**EM_f_ + 1) ≈ 1.4. This value would require that 30% of monomeric **AD** exists in the folded state, which is not consistent with the NMR chemical shift data, suggesting that assumption that the parallel and antiparallel backbone arrangements are equally populated in the ***c*-AA·DD** duplex is reasonable.

### 2.3 Molecular mechanics calculations

**Fig. 7:**
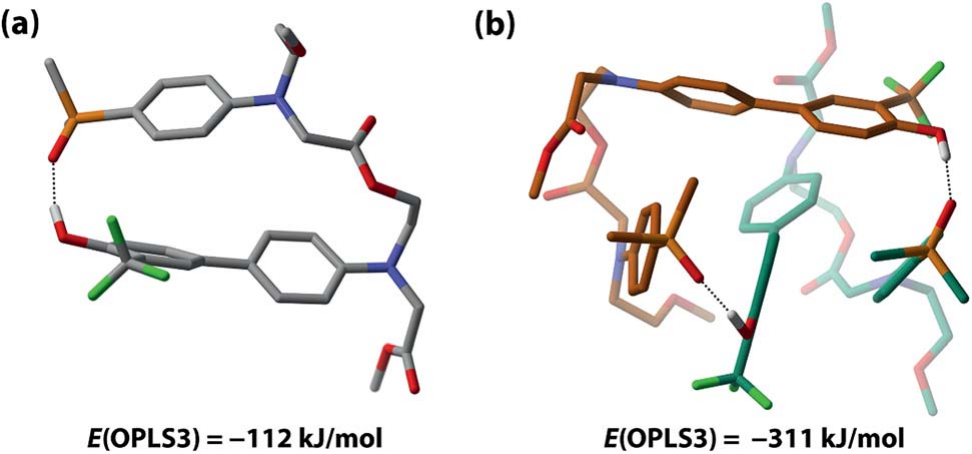
Lowest-energy structures of (a) **AD** and (b) **AD·AD**. The OPLS3 force field with implicit chloroform solvation model was employed. Protecting groups and alkyl groups on phosphorus were replaced with methyl groups. Carbon atoms are colour coded by molecule, and hydrogen atoms are not shown for clarity. Hydrogen bonds are shown as dotted lines.

The competition between duplex formation and intramolecular 1,2-folding in **AD** were further investigated using molecular mechanics calculations. The OPLS3 force field with implicit chloroform solvation model was employed, as implemented in the MacroModel software (the experiments were carried out in toluene, but chloroform is the only non-polar implicit solvent model implemented).^52^ A conformational search was performed on the **AD** monomer and the lowest energy structure, shown in Fig. 7(a), is a folded species. The calculation is clearly inconsistent with the experimental results, reinforcing our previous findings that computational methods do not provide a reliable method for predicting the self-assembly properties of synthetic molecules of this complexity.^39^ To investigate whether this folded structure is strongly preferred over duplex formation by the force-field, two molecules of **AD** were constrained to have one intermolecular hydrogen bond, and a conformational search gave the closed ***c*-AD·AD** duplex shown in Fig. 7(b) as the lowest energy structure. No open ***o*-AD·AD** structures were found within 5 kJ mol^–1^ of the minimum. The calculated energy of the duplex is 87 kJ mol^–1^ lower than the energy of two folded monomers, which suggests that there is considerable strain associated with folding in this system.

### 2.4 Double hydrogen bonding

Oxygen hydrogen bond acceptors can interact with more than one hydrogen bond donor, which can degrade the fidelity of sequence-selective duplex formation.^31^ In order to investigate whether the base-pair recognition system used here would suffer from this problem, **A** was titrated into **DD**. The changes in ^19^F NMR chemical shift of the **DD** did not fit to a 1 : 1 isotherm (see ESI†), so a 1 : 2 binding model was investigated:6
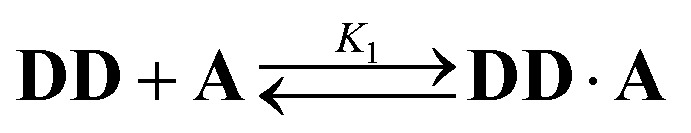

7
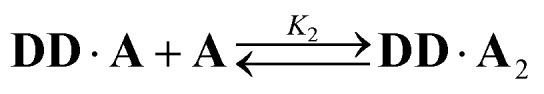



**Fig. 8:**
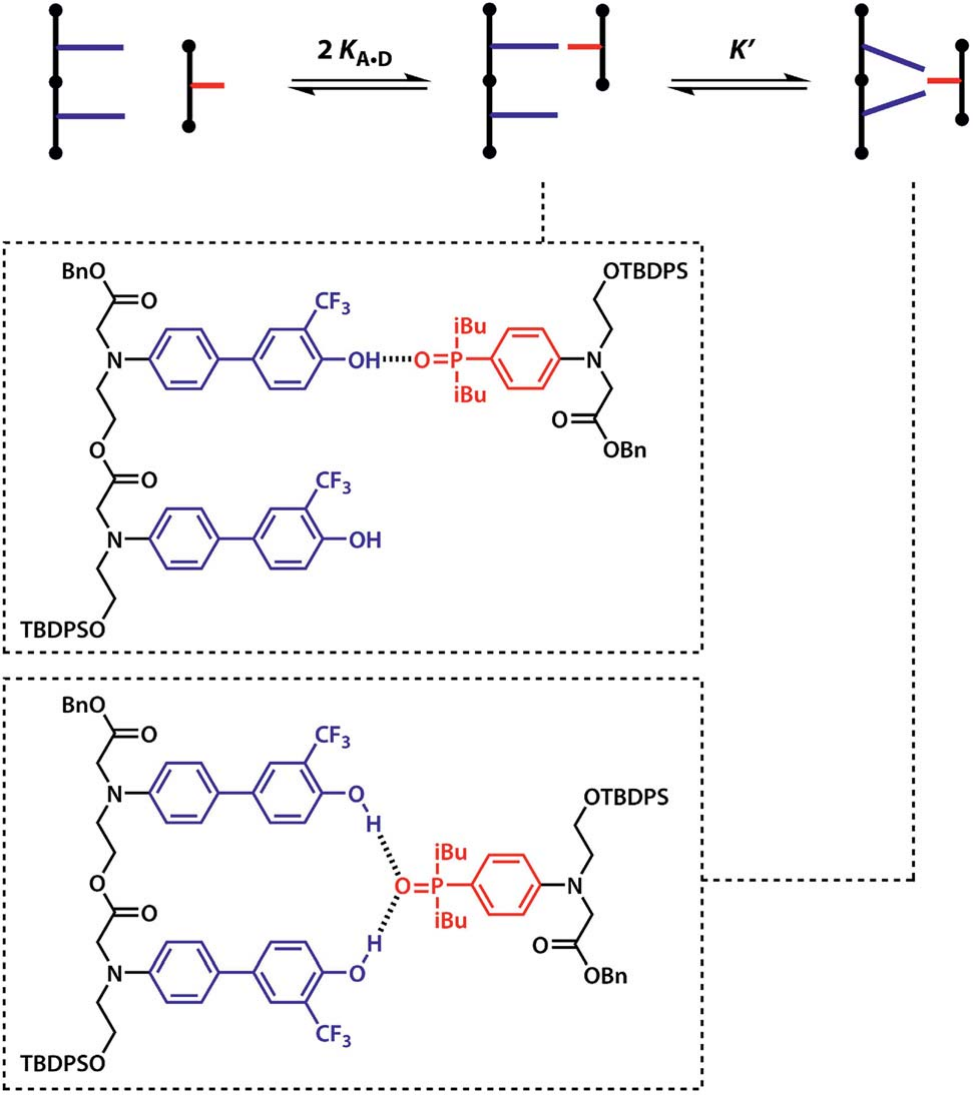
Pathway towards formation of a double hydrogen bond between **DD** 2-mer and **A**. *K***_A·D_** is the intermolecular association constant for formation a single **A·D** hydrogen bond, *K*′ is the association constant for the interaction of the second phenol with the same phosphine oxide. Statistical factors represent the degeneracy of the structures involved.

The two donor binding sites were assumed to be independent and identical, hence *K*_1_*K*_2_ = *K*_**A·D**_^2^ could be fixed in the least squares regression analysis. The association constant for the **DD·A** was determined to be *K*_1_ = (15 000 ± 2000) M^–1^, which is four times greater than the single hydrogen bond association constant *K***_A·D_** and suggests additional stabilisation due to a hydrogen bond between the second phenol and the phosphine oxide. We can represent the equilibria leading to the doubly bonded complex as in Fig. 8.

Noting that both 1 : 1 complexes give rise to the observed association constant, *K*_1_ can be expressed as:

8 *K*_1_ = 2*K*_**A·D**_ + 2*K*_**A·D**_*K*′

The association constant for the formation of the second hydrogen bond is, therefore:9
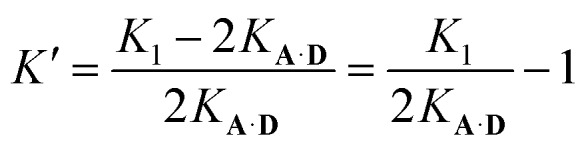



Using eqn (9) and the measured value for *K*_1_, the association constant for the interaction of the second phenol donor with the acceptor is *K*′ (1.0 ± 0.2), which means that the double-bonded complex represents 50% of the 1 : 1 complex. The ratio of *K***_A·D_**EM_i_ and *K*′ describes the competition between a correctly recognised duplex and a doubly hydrogen-bonded mismatched complex. This ratio is 80 for this system, therefore sequence selectivity should be achieved for longer information oligoesters with fidelity of 99%. For comparison, the previously reported sequence-containing information oligomer shows *K*′ = 1.6 and *K***_A·D_**EM_i_ = 9.9, hence exhibits sequence fidelity of 86%.^31^,^39^ While the value of *K*′ for the system described here is comparable with that reported earlier, the exceptionally strong hydrogen-bonding interaction between the recognition units should lead to superior performance the formation of closed duplexes with high sequence fidelity.

## 3 Conclusions

In conclusion, candidates for new information molecules were synthesised and their behaviour in toluene was studied through ^19^F and ^31^P NMR spectroscopy. The monomeric building blocks are readily accessible and 2-mers were easily synthesised through efficient ester coupling reactions, with scope for the synthesis of longer oligomers using the same methodology. A long-short base-pairing scheme akin to purines and pyrimidines in natural nucleic acids was employed in order to reduce intramolecular folding and a flexible backbone was used to ensure the geometric complementarity required for duplex formation. Homo- and hetero-2-mers were observed to form stable duplexes in toluene at 298 K with effective molarities for duplex formation of 20 mM and without any substantial 1,2-folding. The observed trends were consistent with those previously reported using ^31^P NMR, thus providing a convenient handle for studying supramolecular association. Formation of double hydrogen bonds to the oxygen-based acceptor was found to be much less favoured than the desired base-pairing interactions. This system appears to be ideally suited to the synthesis of longer oligomers which are expected to show the possibility of high-fidelity sequence-specific information recognition *via* hydrogen bonding in organic solvents.

## Conflicts of interest

There are no conflicts to declare.

## Supplementary Material

Supplementary information
