# Supplementary material for: Building blocks for recognition-encoded oligoesters that form H-bonded duplexes
Source: Chem Sci. 2019 Jan 11;10(8):2444–51. doi: 10.1039/c8sc04896g (PMC6385898; doi:10.1039/c8sc04896g)
Supplement: Supplementary file 1 [file SC-010-C8SC04896G-s001.pdf]

Electronic Supplementary Material (ESI) for Chemical Science.
This journal is © The Royal Society of Chemistry 2019

Building blocks for recognition-encoded oligoesters that form H-bonded duplexes

Supplementary Information

Filip T. Szczypiński and Christopher A. Hunter

Contents

1 **General Methods** 3

2 **Synthesis** 4

2.1 Synthesis of **2** 4
2.2 Synthesis of **4** 6
2.3 Synthesis of **6** 7
2.4 Synthesis of **7** 8
2.5 Synthesis of **8** 10
2.6 Synthesis of **9** 12
2.7 Synthesis of **10** 14
2.8 Synthesis of **11** 17
2.9 Synthesis of **12** 19
2.10 Synthesis of **13** 21
2.11 Synthesis of **14** 23
2.12 Synthesis of **15** 25
2.13 Synthesis of **16** 27
2.14 Synthesis of **17** 30
2.15 Synthesis of **18** 32

3 **1:1 Binding Isotherm Derivation and Implementation** 35

4 **Dimerisation Isotherm Derivation and Implementation** 37

5 **Implementation of 1:2 Binding Isotherm** 39

6 NMR Studies **41**

6.1 **D · A** Binding Isotherm - Repetition 1 41

6.2 **D · A** Binding Isotherm - Repetition 2 42

6.3 **D · A** Binding Isotherm - Repetition 3 43

6.4 **DD · AA** Binding Isotherm - Repetition 1 44

6.5 **DD · AA** Binding Isotherm - Repetition 2 45

6.6 **AD** Dimerisation Isotherm - Repetition 1 46

6.7 **AD** Dimerisation Isotherm - Repetition 2 47

7 Double Hydrogen Bonding **48**

7.1 **DD · A** 1:1 Binding Isotherm - Repetition 1 51

7.2 **DD · A** 1:1 Binding Isotherm - Repetition 2 51

7.3 **DD · A** 1:2 Binding Isotherm (Identical Sites, Fixed Values) - Repetition 1 52

7.4 **DD · A** 1:2 Binding Isotherm (Identical Sites, Fixed Values) - Repetition 2 52

7.5 **DD · A** 1:2 Binding Isotherm (Identical Sites) - Repetition 1 53

7.6 **DD · A** 1:2 Binding Isotherm (Identical Sites) - Repetition 2 53

7.7 **DD · A** 1:2 Binding Isotherm (Independent Sites) - Repetition 1 54

7.8 **DD · A** 1:2 Binding Isotherm (Independent Sites) - Repetition 2 54

8 Molecular Modelling **55**

References **55**

1 General Methods

Commercial reagents were used as received without further purification. Dichloromethane and tetrahydrofurane were purified and dried using PureSolv MD 5 Solvent Purification System. Routine and characterisation NMR spectra were recorded on Bruker 400 MHz Avance III HD Smart Probe, 400 MHz Smart Probe, and 400 MHz Avance III HD Spectrometers at 298 K and using Wilmard 5 mm Thin Wall Precision NMR sample tubes. NMR titrations and dilutions were performed on Bruker 400 MHz Avance III HD Smart Probe Spectrometer. Upon each addition, the solution was manually shaken before acquiring the spectrum, which was sufficient time for equilibration to be reached. Chemical shifts for ^{1}H, ^{13}C, ^{19}F, and ^{31}P are reported in ppm on the δ scale; ^{1}H and ^{13}C were referenced to the residual solvent peak; ^{19}F and ^{31}P were unreferenced. Coupling constants (J) are reported in hertz (Hz). The following abbreviations are used to describe signal multiplicity for ^{1}H and ^{13}C NMR spectra: s: singlet, d: doublet, t: triplet, m: multiplet, br: broad. High resolution electrospray ionization mass spectrometry (HRMS-ESI) was performed on Waters LCT Premier TOF Spectrometer or by the Mass Spectrometry Service at the Department of Chemistry. Melting point measurements were performed on Mettler Toledo MP90. Infrared (IR) spectra were recorded on Bruker Alpha FTIR Spectrometer with single reflection diamond Platinum ATR. The liquid chromatography mass spectrometry (LCMS) analysis of samples was performed using Waters Acquity H-class UPLC coupled with a single quadrupole Waters SQD2. Acquity UPLC CSH C18 Column, 130Å, 1.7 µm, 2.1 mm x 50 mm was used as the UPLC column. The conditions of the UPLC method were as follows: solvent A: water +0.1% formic acid; solvent B: acetonitrile +0.1% formic acid; gradient of 0-2 minutes 5% - 100%B + 1 minute 100%B with re-equilibration time of 2 minutes. Flow rate: 0.6 ml/min; column temperature of 40 °C; injection volume of 2 µL. The signal was monitored at 254 nm. Chromatographic separations were performed on Teledyne ISCO CombiFlash Rf+UV-Vis and CombiFlash Rf+Lumen, using prepacked cartridges of silica (25 µm or 50 µm PuriFlash Columns). Mixtures were solid loaded using silica gel 60 (Merck, 40–63 µm). The signal was monitored at 254 nm and (if CombiFlash Rf+Lumen) using evaporative light scattering detector.

2 Synthesis

2.1 Synthesis of 2

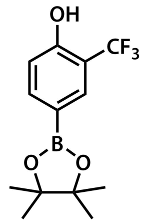

To a degassed solution of 4-bromo-2-(trifluoromethyl)phenol (1.7 g, 7.0 mmol), bis(pinacolato)diboron (2.1 g, 8.4 mmol), and potassium acetate (1.1 g, 10.5 mmol) in dioxane (20 ml) was added [1,1'-bis(diphenylphosphino)ferrocene]dichloropalladium(II) (572 mg, 0.7 mmol, complex with CH_2Cl_2). The mixture was heated under reflux for three hours, cooled down, and filtered through Celite. The residue was washed with ethyl acetate (5 × 10 ml). Combined organic layers were concentrated under vacuum and the residue was purified using column chromatography (0-40% ethyl acetate in petroleum ether) to yield product **2** as a white waxy solid (2.0 g, 5.2 mmol, 73%).

^{1}H NMR (400 MHz, $CDCl_3$) δ 7.98 (s, 1H), 7.83 (d, J = 7.8 Hz, 1H), 6.91 (d, J = 8.1 Hz, 1H), 6.29 (s, 1H), 1.34 (s, 12H) ppm.

^{13}C NMR (101 MHz, $CDCl_3$) δ 156.4 (q, J = 2 Hz), 140.0, 133.8 (q, J = 5 Hz), 124.0 (q, J = 273 Hz), 120.4 (br), 116.9, 116.3 (q, J = 30 Hz), 84.2, 24.8 ppm.

^{19}F NMR (376 MHz, $CDCl_3$) -61.0 ppm.

FT-IR (neat): 3232.8 (br), 2983.7, 1613.0 cm^{-1}.

HR-MS (ESI): m/z = 289.1220 $[M+H]^+$ (calcd. for $C_{13}H_{17}O_3F_3{}^{11}B$: 289.1223, Δ -1.0 ppm).

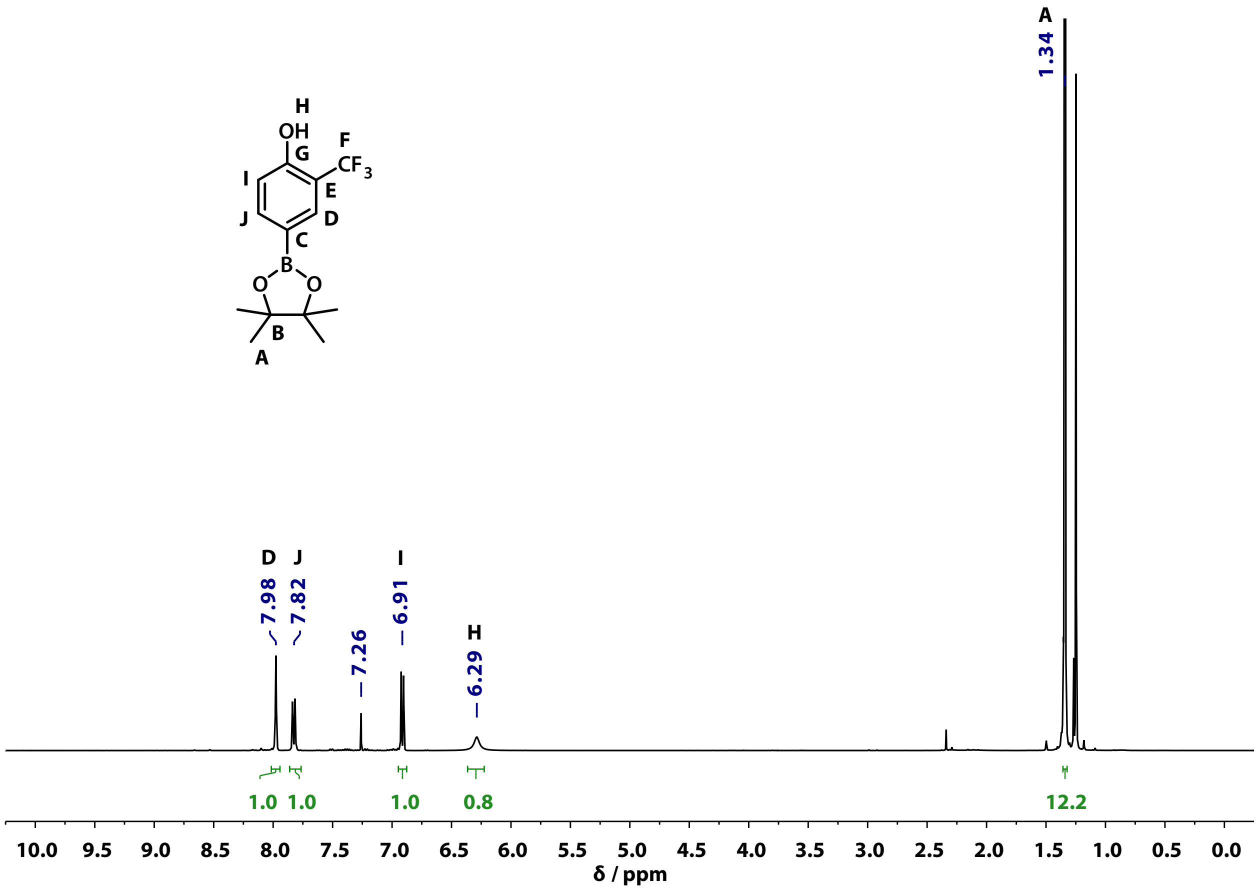
H
OH
G
F
CF3
I
E
J
D
C
B
O
O
B
A
A
1.34
D J
7.98
7.82
7.26
I
6.91
H
6.29
1.0 1.0
1.0
0.8
12.2
10.0 9.5 9.0 8.5 8.0 7.5 7.0 6.5 6.0 5.5 5.0 4.5 4.0 3.5 3.0 2.5 2.0 1.5 1.0 0.5 0.0
δ / ppm

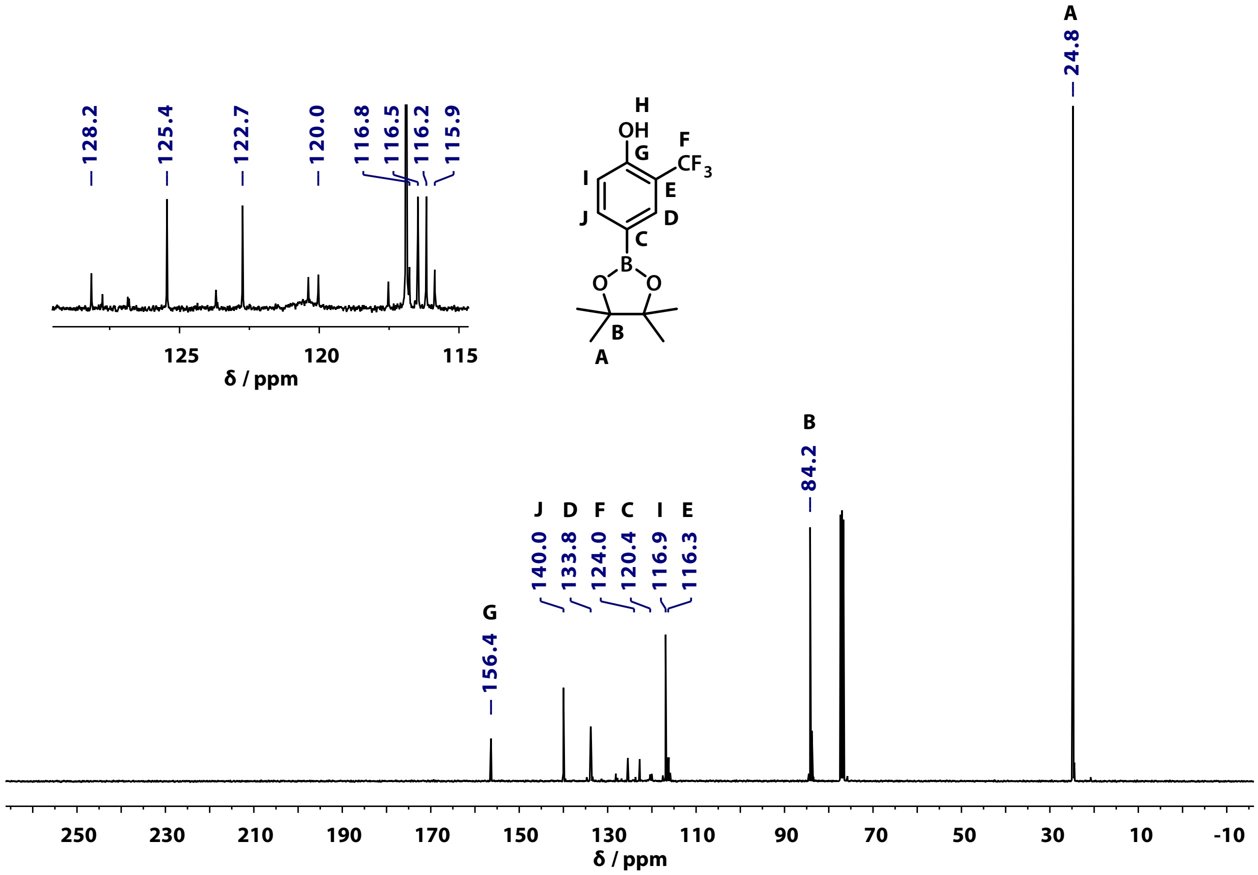
128.2
125.4
122.7
120.0
116.8
116.5
116.2
115.9
125 120 115
δ / ppm
H
OH
G
F
CF3
I
E
J
D
C
B
O
O
B
A
A
24.8
B
84.2
J D F C I E
140.0
133.8
124.0
120.4
116.9
116.3
G
156.4
250 230 210 190 170 150 130 110 90 70 50 30 10 -10
δ / ppm

2.2 Synthesis of 4

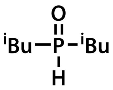

A solution of diethyl phosphite (3.3 ml, 3.6 g, 25.9 mmol) in tetrahydrofuran (50 ml) was added dropwise to a solution of *iso*-butylmagnesium chloride (2 M in Et_2O, 38.8 ml, 77.6 mmol) at 0 °C over 15 minutes. The mixture was stirred at 0 °C for 15 minutes and then at room temperature. After two hours, the reaction mixture was cooled to 0 °C and aqueous solution of hydrochloric acid (0.1 M, 50 ml) was added dropwise over 20 minutes. The obtained gel was suspended in dichloromethane (50 ml) and agitated well for 5 minutes. The resulting slurry was filtered through Celite. The residue was washed with dichloromethane (100 ml) and the filtrate layers were separated. Combined organic phases were dried with anhydrous magnesium sulfate, filtered and concentrated under vacuum. The residue was azeotroped with diethyl ether, yielding compound **4** as white solid (4.0 g, 24.8 mmol, 96%), which was used without further purification.

^{1}**H NMR** (400 MHz, $CDCl_3$) δ 7.01 (dtt, J = 444.9, 5.9, 1.9 Hz, 1H), 2.24 – 2.05 (m, 2H), 1.88 – 1.72 (m, 2H), 1.58 – 1.45 (m, 2H), 1.06 (d, J = 6.7 Hz, 12H) ppm. Agrees with the spectra reported in the literature.[1]

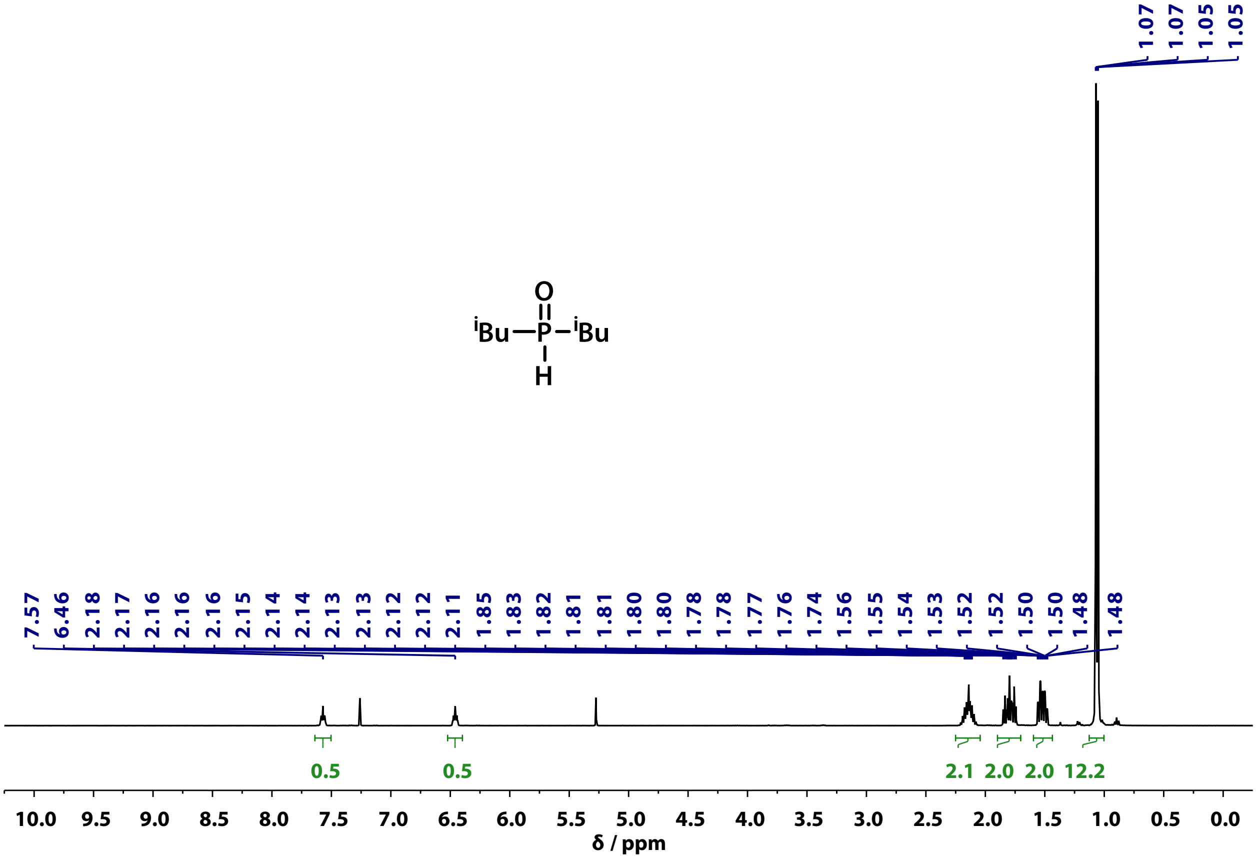

2.3 Synthesis of 6

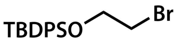

Compound **6** was synthesised according to the literature and the spectra agree with those reported.[2]

^{1}**H NMR** (400 MHz, $CDCl_3$) δ 7.7 – 7.7 (m, 4H), 7.5 – 7.4 (m, 6H), 4.0 (t, J = 6.4 Hz, 2H), 3.5 (t, J = 6.4 Hz, 2H), 1.1 (s, 9H) ppm.

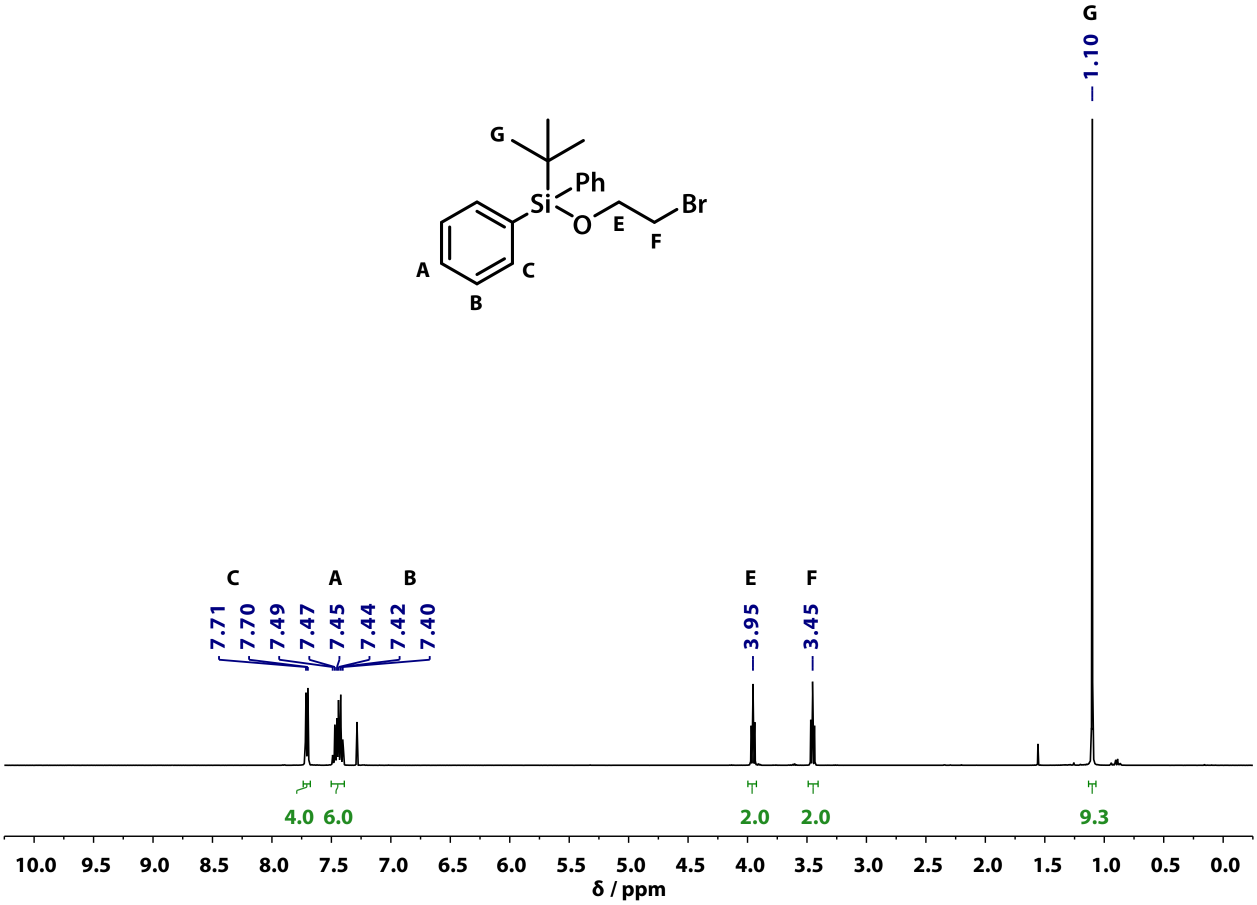

2.4 Synthesis of 7

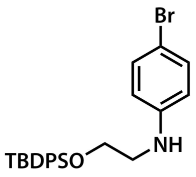

A suspension of 4-bromoaniline (1550 mg, 9.1 mmol), sodium carbonate (1650 g, 18.3 mmol), and compound **6** (2630 mg, 10 mmol) in *N,N*-dimethylformamide (15 ml) was stirred was three days. The mixture was partitioned between ice-cold water (125 ml) and ethyl acetate (30 ml). The layers were separated and the aqueous layer was subsequently washed with ethyl acetate (2 × 30 ml). The combined organic extracts were washed with 5% solution of lithium chloride (3×20 ml) and brine (2×20 ml). The washed solution was dried with anhydrous magnesium sulfate, filtered and concentrated under vacuum. The residue was purified using column chromatography (0-30% dichloromethane in petroleum ether) to yield product **7** as a colourless oil (2.2 g, 4.8 mmol, 53%).

^{1}H NMR (400 MHz, $CDCl_3$) δ 7.8 – 7.7 (m, 4H), 7.5 – 7.4 (m, 6H), 7.3 (d, J = 8.8 Hz, 2H), 6.5 (d, J = 8.8 Hz, 2H), 4.1 (t, J = 5.9 Hz, 1H), 3.9 (t, J = 5.6 Hz, 2H), 3.3 (q, J = 5.9, 5.6 Hz, 2H), 1.1 (s, 9H) ppm.

^{13}C NMR (101 MHz, $CDCl_3$) δ 147.3, 135.6, 133.3, 131.9, 129.9, 127.8, 114.7, 109.0, 62.2, 45.8, 26.9, 19.2 ppm.

FT-IR (neat): 3409, 3070, 2956, 2929, 2856, 1595 cm^{-1}.

HR-MS (ESI): m/z = 454.1190 $[M+H]^+$ (calcd. for $C_{24}H_{29}NOSi^{79}Br$: 454.1202, Δ -2.6 ppm).

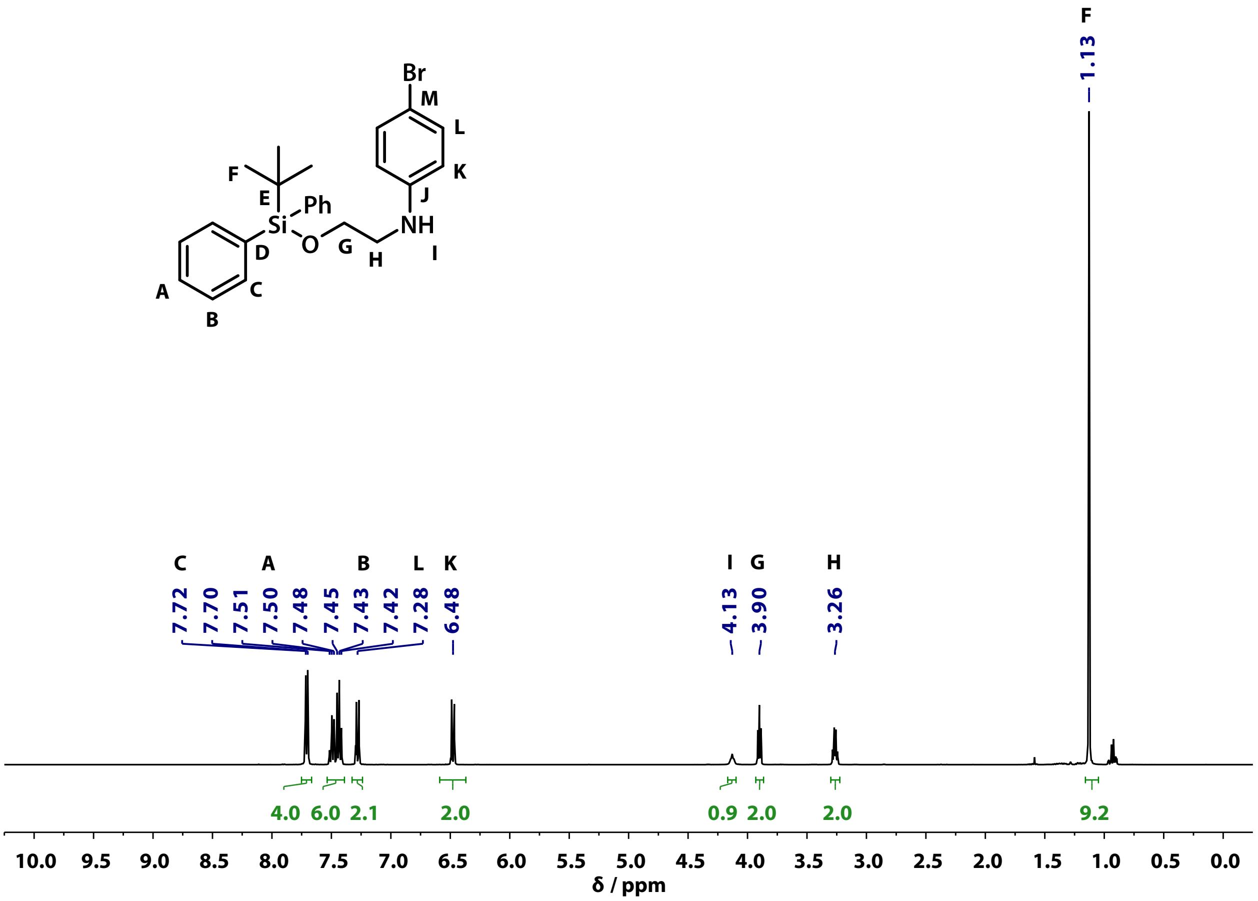
Br
M
L
K
J
F
E
Ph
Si
O
G
H
NH
I
D
A
C
B
F
1.13
C A B L K
7.72 7.70 7.51 7.50 7.48 7.45 7.43 7.42 7.28 6.48
I G H
4.13 3.90 3.26
4.0 6.0 2.1 2.0 0.9 2.0 2.0 9.2
10.0 9.5 9.0 8.5 8.0 7.5 7.0 6.5 6.0 5.5 5.0 4.5 4.0 3.5 3.0 2.5 2.0 1.5 1.0 0.5 0.0
δ / ppm

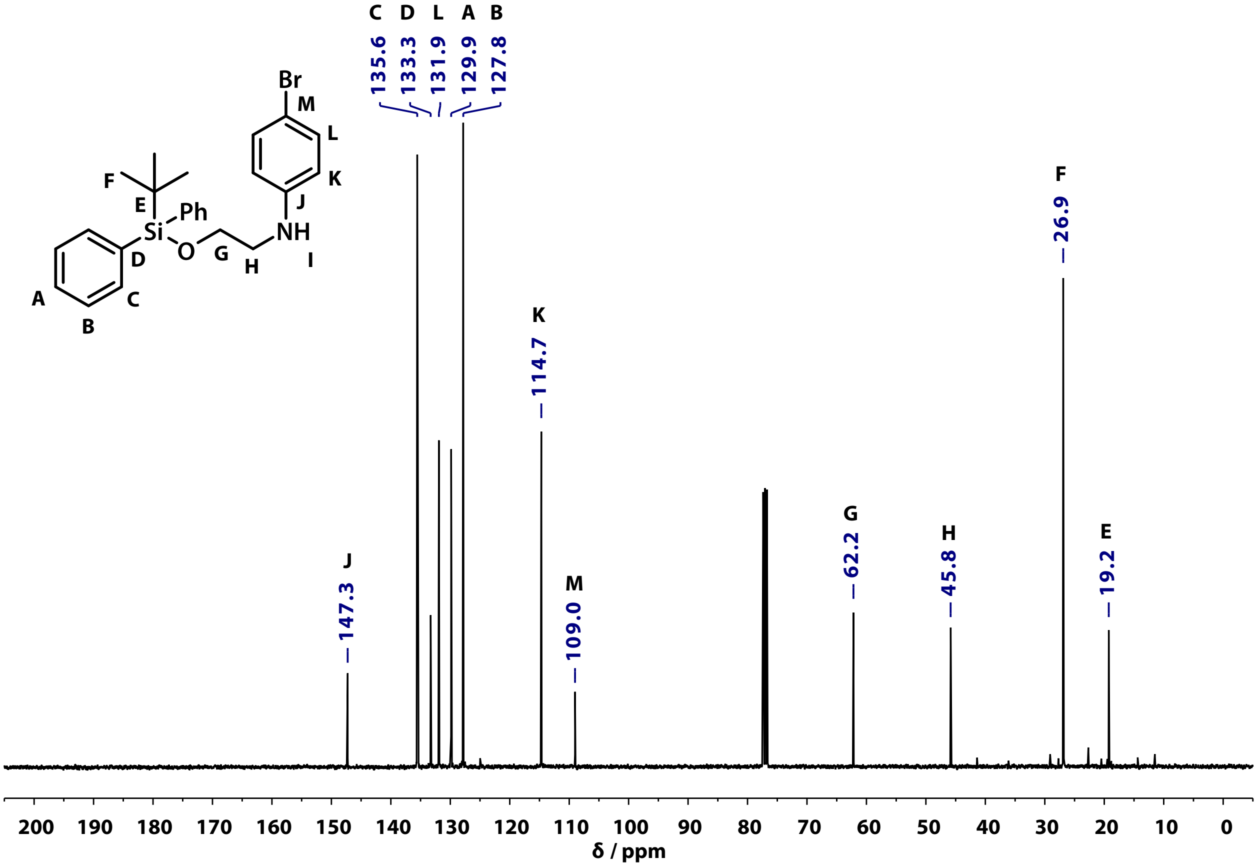
C D L A B
135.6 133.3 131.9 129.9 127.8
Br
M
L
K
J
F
E
Ph
Si
O
G
H
NH
I
D
A
C
B
F
26.9
K
114.7
J
147.3
M
109.0
G
62.2
H
45.8
E
19.2
200 190 180 170 160 150 140 130 120 110 100 90 80 70 60 50 40 30 20 10 0
δ / ppm

2.5 Synthesis of 8

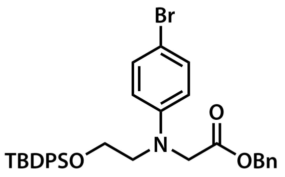

A solution of 7 (2.4 g, 5.3 mmol), benzyl bromoacetate (1.8 ml, 2.4 g, 10.6 mmol), and *N,N*-diisopropylethylamine (1.9 ml, 1.4 g, 10.6 mmol) in *N,N*-dimethylformamide (8 ml) was stirred at 80 °C overnight under nitrogen atmosphere. The reaction mixture then diluted with water (100 ml) and extracted with ethyl acetate (3 × 20 ml). The combined organic extracts were washed with 5 % solution of lithium chloride (4 × 10 ml) and with brine (2 × 20 ml). The washed solution was dried with anhydrous magnesium sulfate, filtered and concentrated under vacuum. The residue was purified using column chromatography (0-30% dichloromethane in petroleum ether) to yield product **8** as a colourless oil (2.6 g, 4.5 mmol, 84%).

^{1}H NMR (400 MHz, $CDCl_3$) δ 7.69 – 7.58 (m, 4H), 7.49 – 7.29 (m, 11H), 7.18 (d, J = 9.1 Hz, 2H), 6.33 (d, J = 9.1 Hz, 2H), 5.16 (s, 2H), 4.05 (s, 2H), 3.83 (t, J = 6.3 Hz, 2H), 3.52 (t, J = 6.3 Hz, 2H), 1.06 (s, 9H) ppm.

^{13}C NMR (101 MHz, $CDCl_3$) δ 170.6, 146.8, 135.5, 135.4, 133.2, 131.8, 129.8, 128.6, 128.4, 128.2, 127.7, 113.5, 109.0, 66.7, 61.2, 53.9, 53.1, 26.8, 19.0 ppm.

FT-IR (neat): 3070, 3046, 2957, 2930, 2857, 1744 cm^{-1}.

HR-MS (ESI): m/z = 602.1737 $[M+H]^+$ (calcd. for $C_{33}H_{37}NO_3Si^{79}Br$: 602.1726, Δ 1.8 ppm).

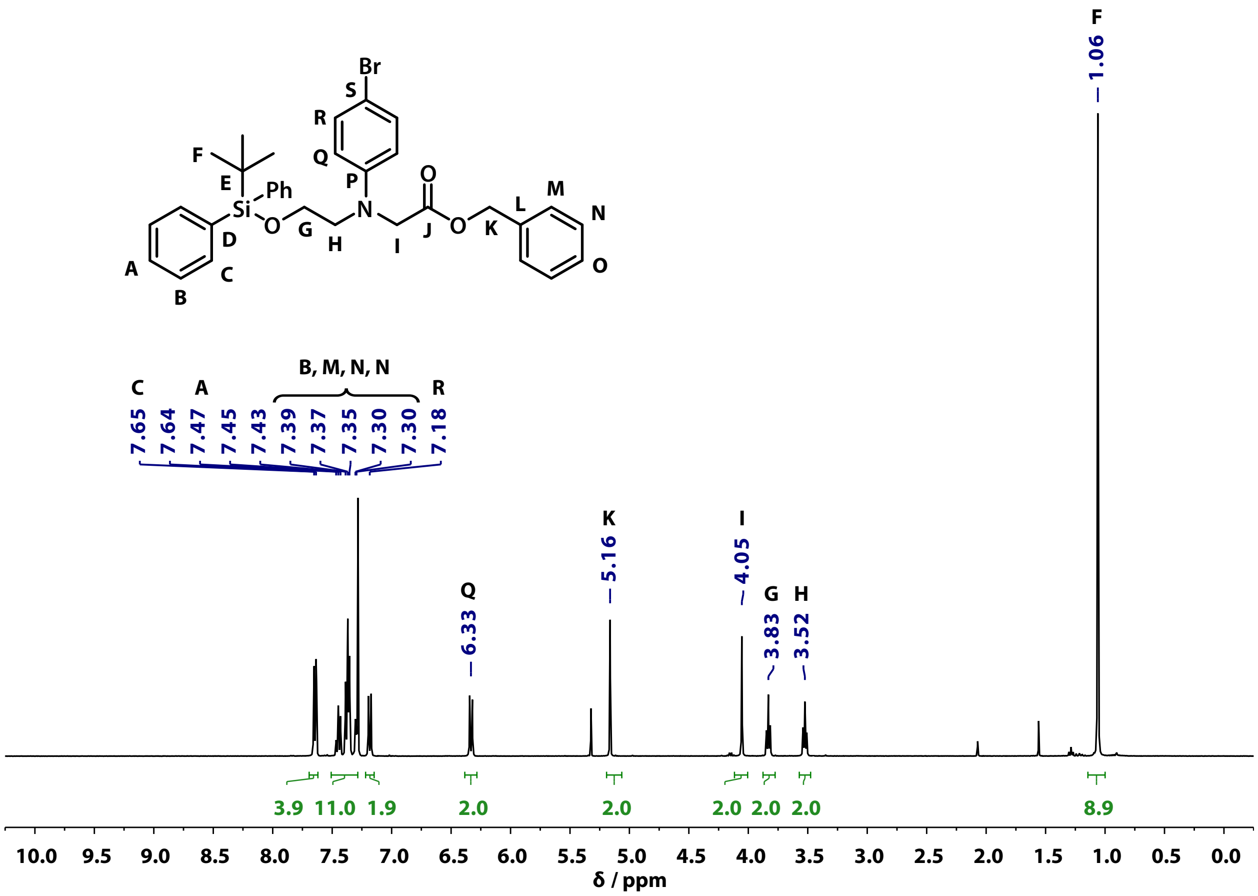
Br
Si
Ph
O
N
A B C D E F G H I J K L M N O P Q R S
C A B, M, N, N R
7.65 7.64 7.47 7.45 7.43 7.39 7.37 7.35 7.30 7.30 7.18
Q 6.33
K 5.16
I 4.05
G 3.83
H 3.52
F 1.06
3.9 11.0 1.9 2.0 2.0 2.0 2.0 2.0 8.9
10.0 9.5 9.0 8.5 8.0 7.5 7.0 6.5 6.0 5.5 5.0 4.5 4.0 3.5 3.0 2.5 2.0 1.5 1.0 0.5 0.0
δ / ppm

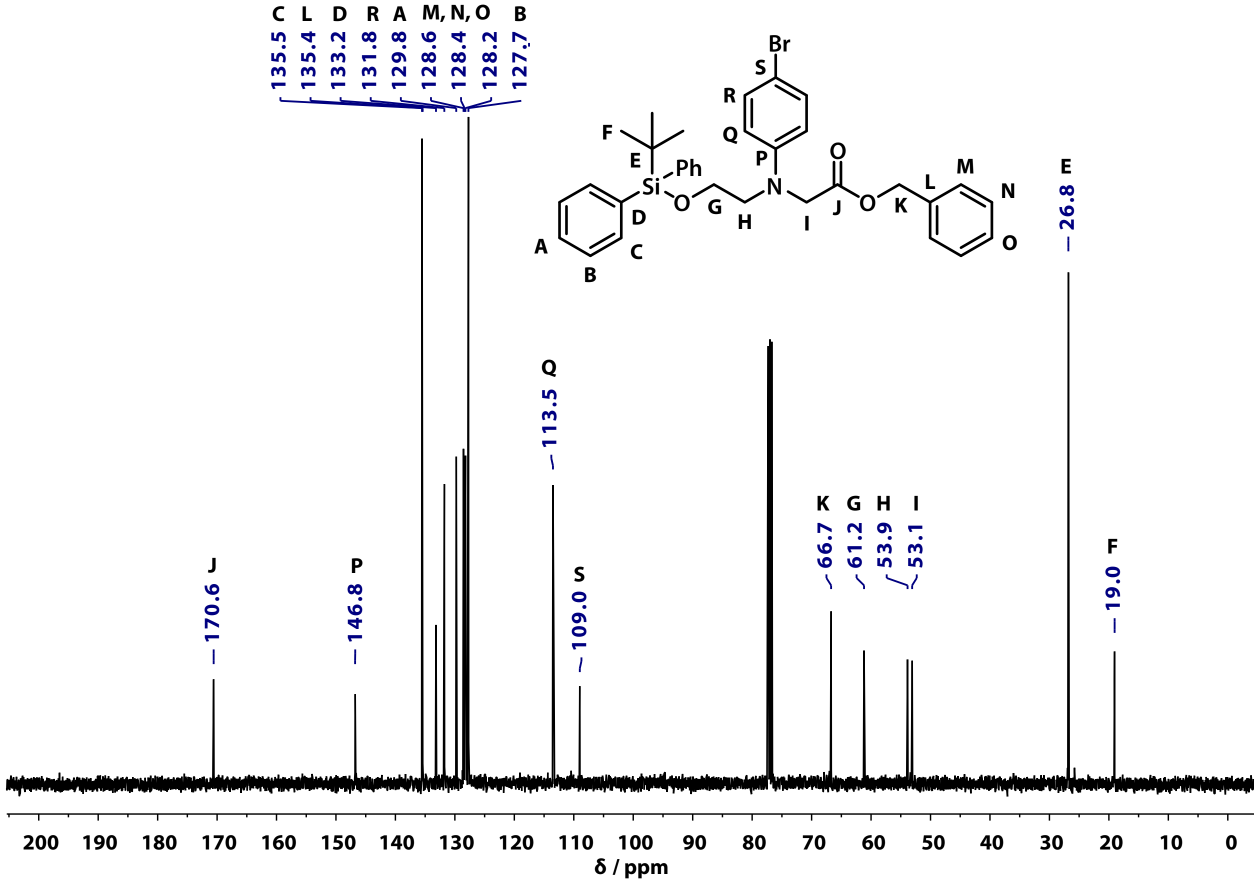
C L D R A M, N, O B
135.5 135.4 133.2 131.8 129.8 128.6 128.4 128.2 127.7
J 170.6
P 146.8
Q 113.5
S 109.0
K 66.7
G 61.2
H 53.9
I 53.1
E 26.8
F 19.0
200 190 180 170 160 150 140 130 120 110 100 90 80 70 60 50 40 30 20 10 0
δ / ppm

2.6 Synthesis of 9

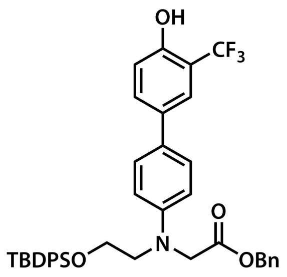

A degassed solution of potassium phosphate (12 ml, 0.5 M) was added to solution of bromide **8** (1.8 g, 3.0 mmol), boronic acid **2** (1.3 g, 4.5 mmol), and XPhos-Pd-G2 pre-catalyst (71 mg, 0.1 mmol) in dry tetrahydrofuran (6 ml) under nitrogen atmosphere. The mixture was stirred at 45 °C overnight, cooled down and filtered through Celite. The filtrate was diluted with water (15 ml) and extracted with ethyl acetate (3 × 10 ml). The combined organic extracts were washed with brine (20 ml), dried with anhydrous magnesium sulfate, filtered and concentrated under vacuum. The residue was purified using column chromatography (0-30% ethyl acetate in petroleum ether) to yield product **9** as a yellow oil (1.1 g, 1.6 mmol, 54%).

^{1}H NMR (400 MHz, $CDCl_3$) δ 7.69 – 7.65 (m, 4H), 7.63 (d, J = 2.0 Hz, 1H), 7.54 (dd, J = 8.4, 2.0 Hz, 1H), 7.48 – 7.30 (m, 15H), 6.97 (d, J = 8.4 Hz, 1H), 6.54 (d, J = 8.9 Hz, 2H), 5.45 (br s, 1H), 5.19 (s, 2H), 4.14 (s, 2H), 3.89 (t, J = 6.2 Hz, 2H), 3.61 (t, J = 6.2 Hz, 2H), 1.07 (s, 9H) ppm.

^{13}C NMR (101 MHz, $CDCl_3$) δ 171.4, 152.1, 146.9, 135.6, 135.4, 133.6, 133.3, 131.0, 129.7, 128.6, 128.4, 128.3, 128.2, 127.7, 127.4, 125.6 (q, J = 273 Hz), 124.1 (q, J = 5 Hz), 117.9, 116.5 (q, J = 30 Hz), 112.1, 66.9, 61.5, 53.8, 53.1, 26.8, 19.1 ppm.

^{19}F NMR (376 MHz, $CDCl_3$) δ -60.7 ppm.

FT-IR (neat): 3380 (br), 3069, 2957, 2930, 2857, 1732, 1613, 1504 cm^{-1}.

HR-MS (ESI): m/z = 684.2780 $[M+H]^+$ (calcd. for $C_{40}H_{41}F_3NO_4Si$: 684.2757, Δ 3.4 ppm).

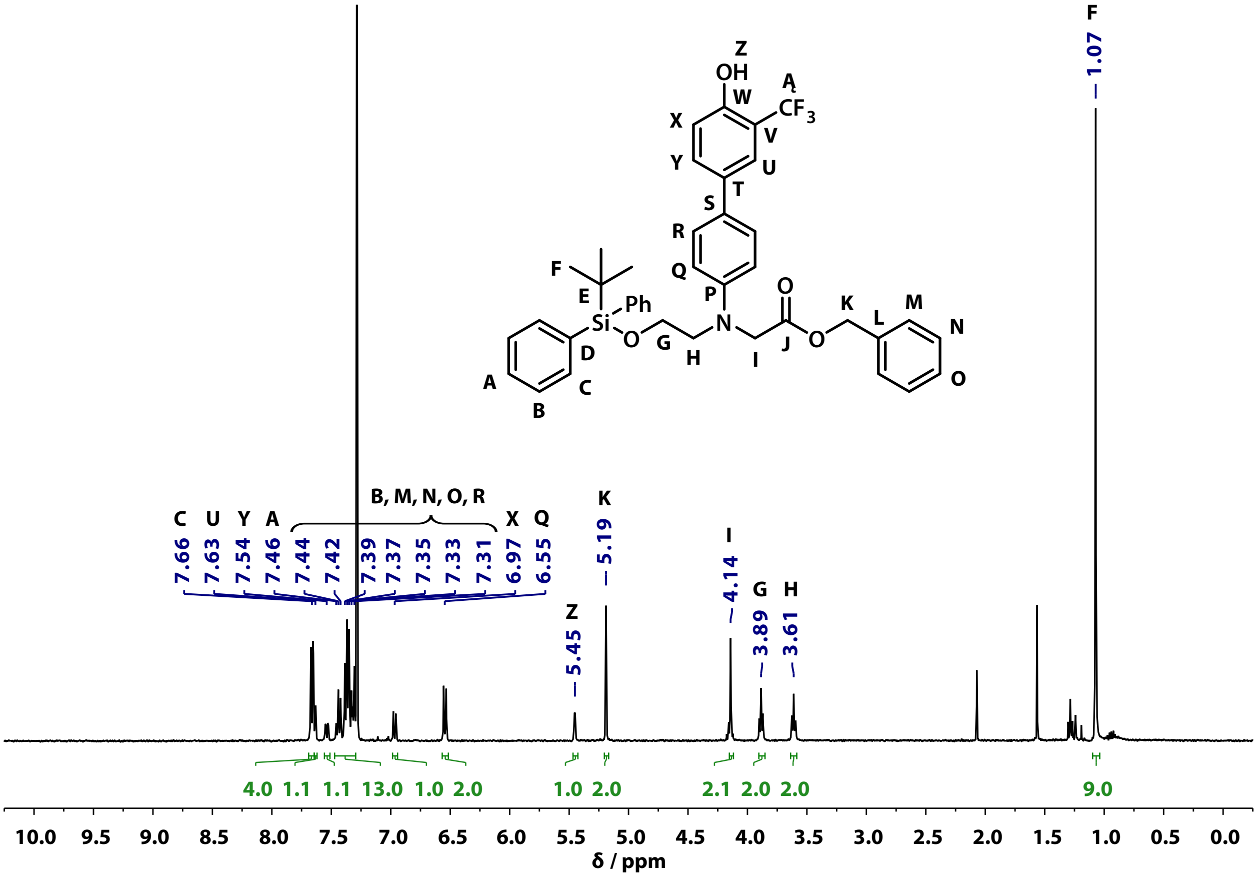

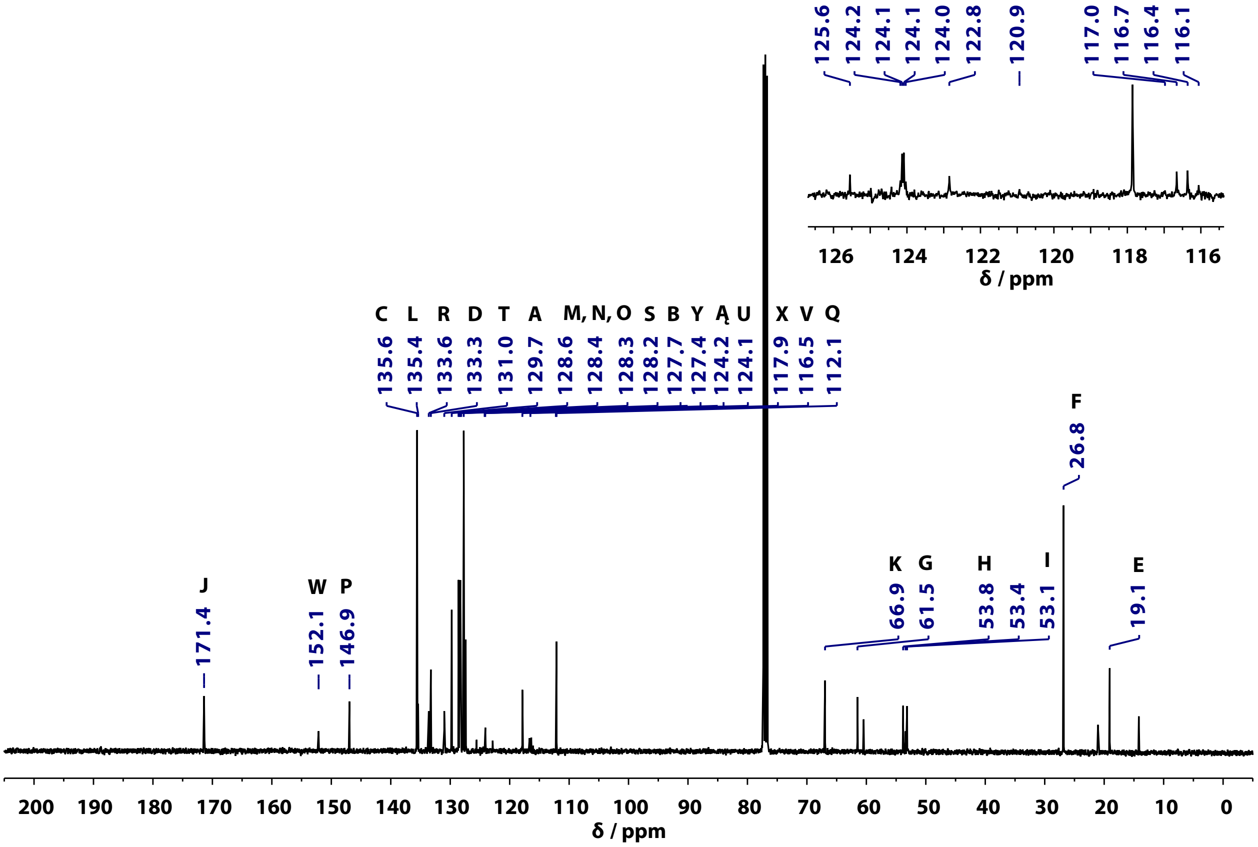

2.7 Synthesis of 10

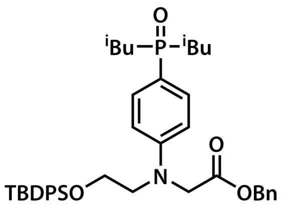

A solution of bromide **8** (1.8 g, 3.0 mmol), compound **4** (511 mg, 3.2 mmol), caesium carbonate (1.5 g, 4.5 mmol), XantPhos (52 mg, 0.1 mmol), and tris(dibenzylideneacetone)dipalladium(0) (28 mg, 0.03 mmol) in dioxane (10 ml) as degassed and stirred at 80 °C for three days. Upon completion, the reaction was diluted with water (30 ml) and extracted with ethyl acetate (3 × 20 ml). The combined organic extracts were washed with brine (20 ml), dried with anhydrous magnesium sulfate, filtered and concentrated under vacuum. The residue was purified using column chromatography (0-100% ethyl acetate in petroleum ether) to yield product **10** as a colourless oil (1.3 g, 1.9 mmol, 63%).

^{1}H NMR (400 MHz, $CDCl_3$) δ 7.61 (d, J = 6.7 Hz, 4H), 7.44 – 7.27 (m, 13H), 6.54 (dd, J = 8.9, 2.1 Hz, 2H), 5.16 (s, 2H), 4.09 (s, 2H), 3.84 (t, J = 6.0 Hz, 2H), 3.59 (t, J = 6.0 Hz, 2H), 2.04 – 1.90 (m, 2H), 1.90 – 1.78 (m, 2H), 1.70 – 1.60 (m, 2H), 1.03 (s, 9H), 1.03 (d, J = 6.5 Hz, 6H), 0.86 (d, J = 6.6 Hz, 6H) ppm.

^{13}C NMR (101 MHz, $CDCl_3$) δ 170.4, 150.0 (d, J = 2 Hz), 135.6, 135.4, 133.1, 131.8 (d, J = 10 Hz), 129.8, 128.6, 128.4, 128.2, 127.8, 119.7 (d, J = 100 Hz), 111.5 (d, J = 12 Hz), 66.9, 61.4, 53.5, 52.8, 40.2 (d, J = 68 Hz), 26.8, 24.8 (d, J = 9 Hz), 24.6 (d, J = 8 Hz), 23.5 (d, J = 3 Hz), 19.1 ppm.

^{31}P NMR(162 MHz, $CDCl_3$) δ 38.9 ppm.

FT-IR (neat): 2954, 2929, 2891, 2867, 1745, 1598, 1512 cm^{-1}.

HR-MS (ESI): m/z = 684.3636 $[M+H]^+$ (calcd. for $C_{41}H_{55}NO_4SiP$: 684.3638, Δ -0.3 ppm).

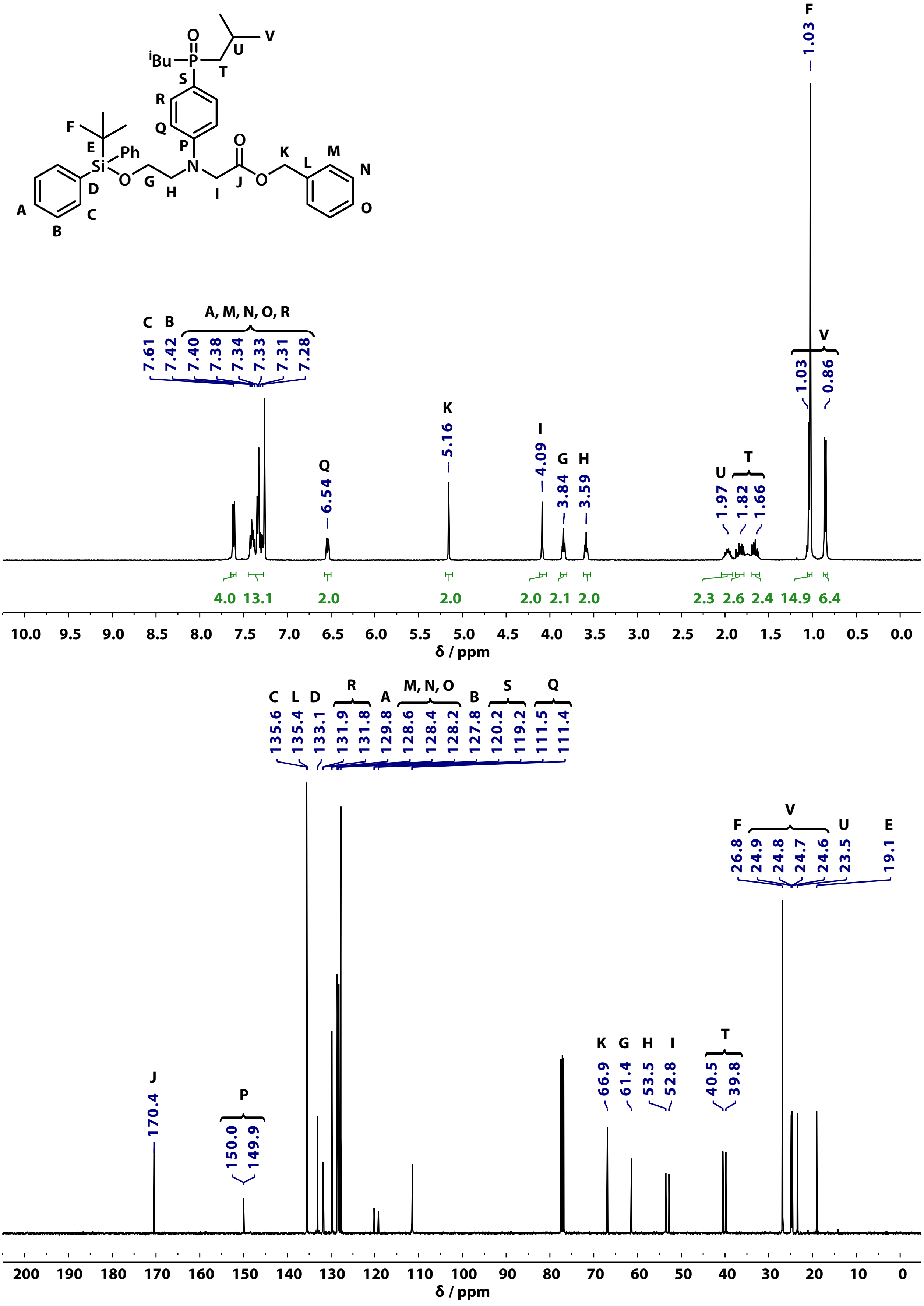

iBu
Ph
Si
O
N
A B C D E F G H I J K L M N O P Q R S T U V
C B A, M, N, O, R
7.61 7.42 7.40 7.38 7.34 7.33 7.31 7.28
Q 6.54
K 5.16
I 4.09
G 3.84
H 3.59
U 1.97
T 1.82 1.66
1.03 0.86
F 1.03
4.0 13.1 2.0 2.0 2.0 2.1 2.0 2.3 2.6 2.4 14.9 6.4
δ / ppm
C L D R A M, N, O B S Q
135.6 135.4 133.1 131.9 131.8 129.8 128.6 128.4 128.2 127.8 120.2 119.2 111.5 111.4
J 170.4
P 150.0 149.9
K G H I
66.9 61.4 53.5 52.8
T 40.5 39.8
F V U E
26.8 24.9 24.8 24.7 24.6 23.5 19.1
δ / ppm

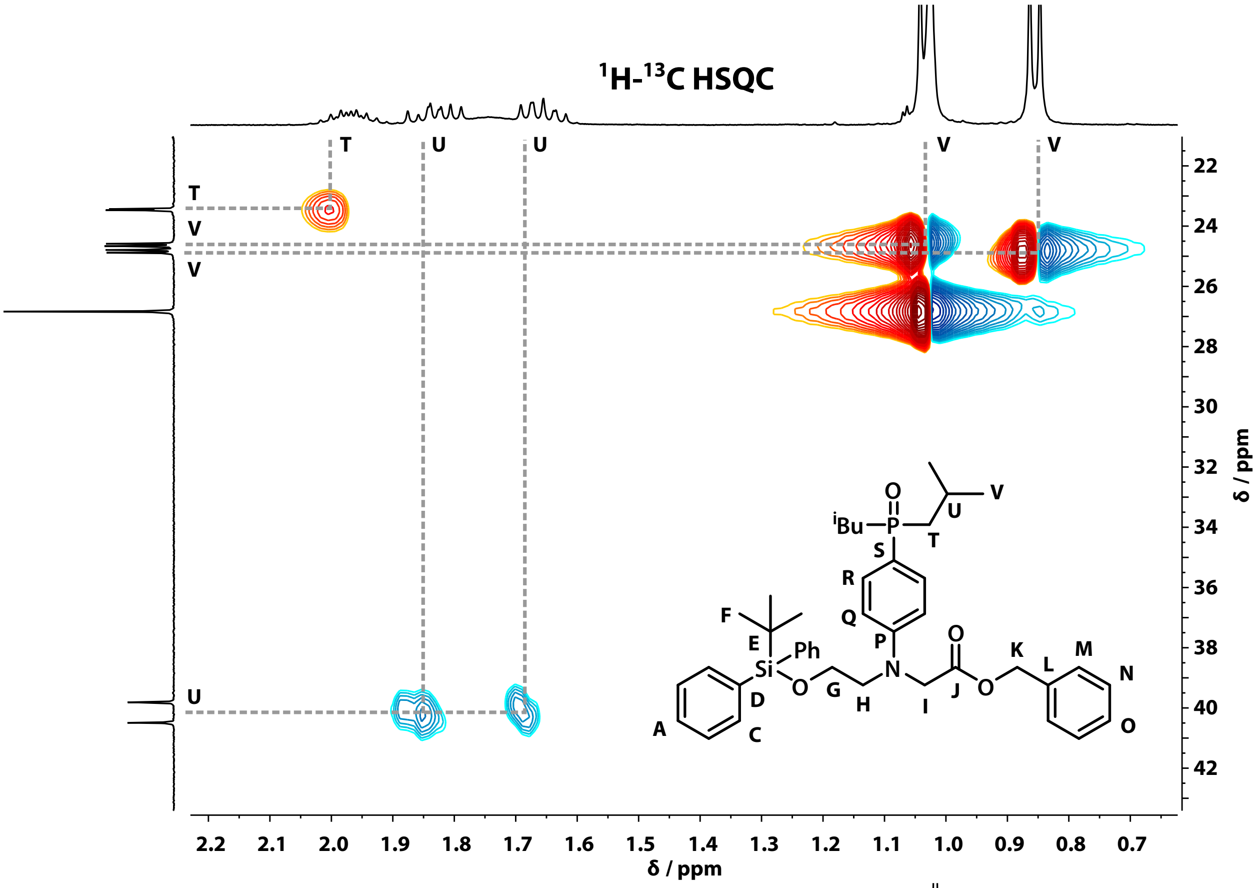

^{1}H-^{13}C HSQC
T
U
U
V
V
T
V
V
U
δ / ppm
δ / ppm
^{i}Bu

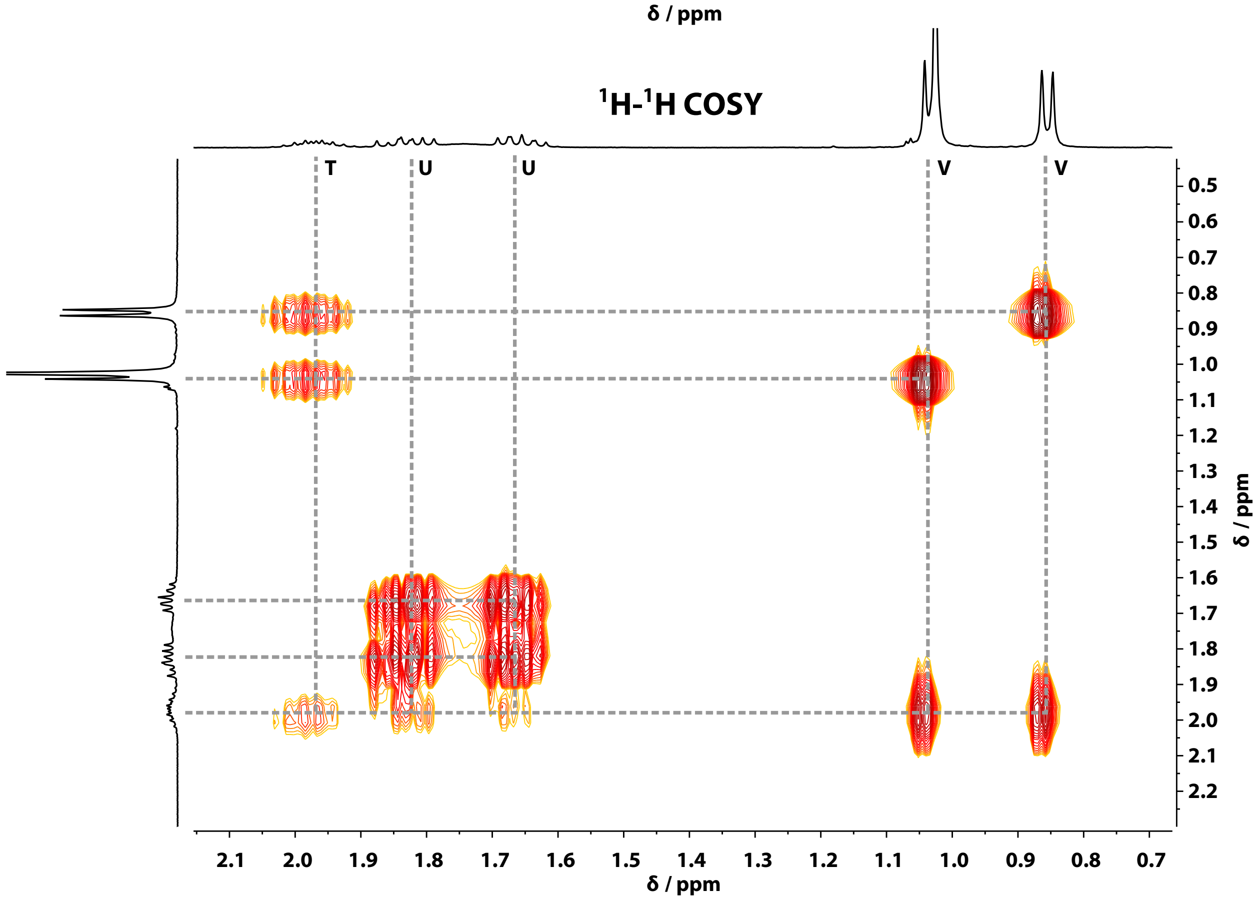

^{1}H-^{1}H COSY
T
U
U
V
V
δ / ppm
δ / ppm

2.8 Synthesis of 11

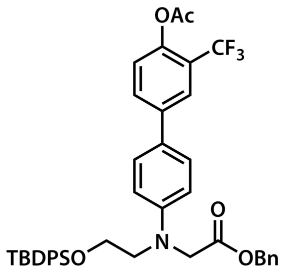

A solution of phenol **10** (300 mg, 0.5 mmol) in acetic anhydride (2.4 ml) and pyridine (2.4 ml) was stirred at ambient temperature overnight. Upon completion, the reaction was diluted with water (30 ml) and extracted with ethyl acetate (3 × 15 ml). The combined organic extracts were washed with water (3 × 15 ml), brine (15 ml), dried with anhydrous magnesium sulfate, filtered and concentrated under vacuum. The residue was purified using column chromatography (0-20% ethyl acetate in petroleum ether) to yield product **11** as a yellow oil (296 mg, 0.4 mmol, 82%).

^{1}H NMR (400 MHz, $CDCl_3$) δ 7.78 (d, J = 2.2 Hz, 1H), 7.73 – 7.65 (m, 5H), 7.48 – 7.41 (m, 2H), 7.40 – 7.29 (m, 11H), 7.25 (d, J = 7.9 Hz, 1H), 6.56 (d, J = 8.9 Hz, 2H), 5.19 (s, 2H), 4.15 (s, 2H), 3.90 (t, J = 6.2 Hz, 2H), 3.62 (t, J = 6.2 Hz, 2H), 2.37 (s, 3H), 1.08 (s, 9H) ppm.

^{13}C NMR (101 MHz, $CDCl_3$) δ 170.8, 169.2, 147.7, 146.2 (br s), 139.4, 135.6, 135.5, 133.3, 130.4, 129.8, 128.6, 128.4, 128.3, 127.9, 127.8, 127.5, 124.6, 124.5 (q, J = 5 Hz), 123.1 (d, J = 273 Hz), 122.9 (q, J = 31 Hz), 112.2, 66.8, 61.4, 53.8, 53.2, 26.9, 20.8, 19.1 ppm.

^{19}F NMR (376 MHz, $CDCl_3$) δ -61.8 ppm.

FT-IR (neat): 2956, 2931, 2858, 1771, 1748, 1611 cm^{-1}.

HR-MS (ESI): m/z = 726.2836 $[M+H]^+$ (calcd. for $C_{42}H_{43}NO_5F_3Si$: 726.2863, Δ -3.7 ppm).

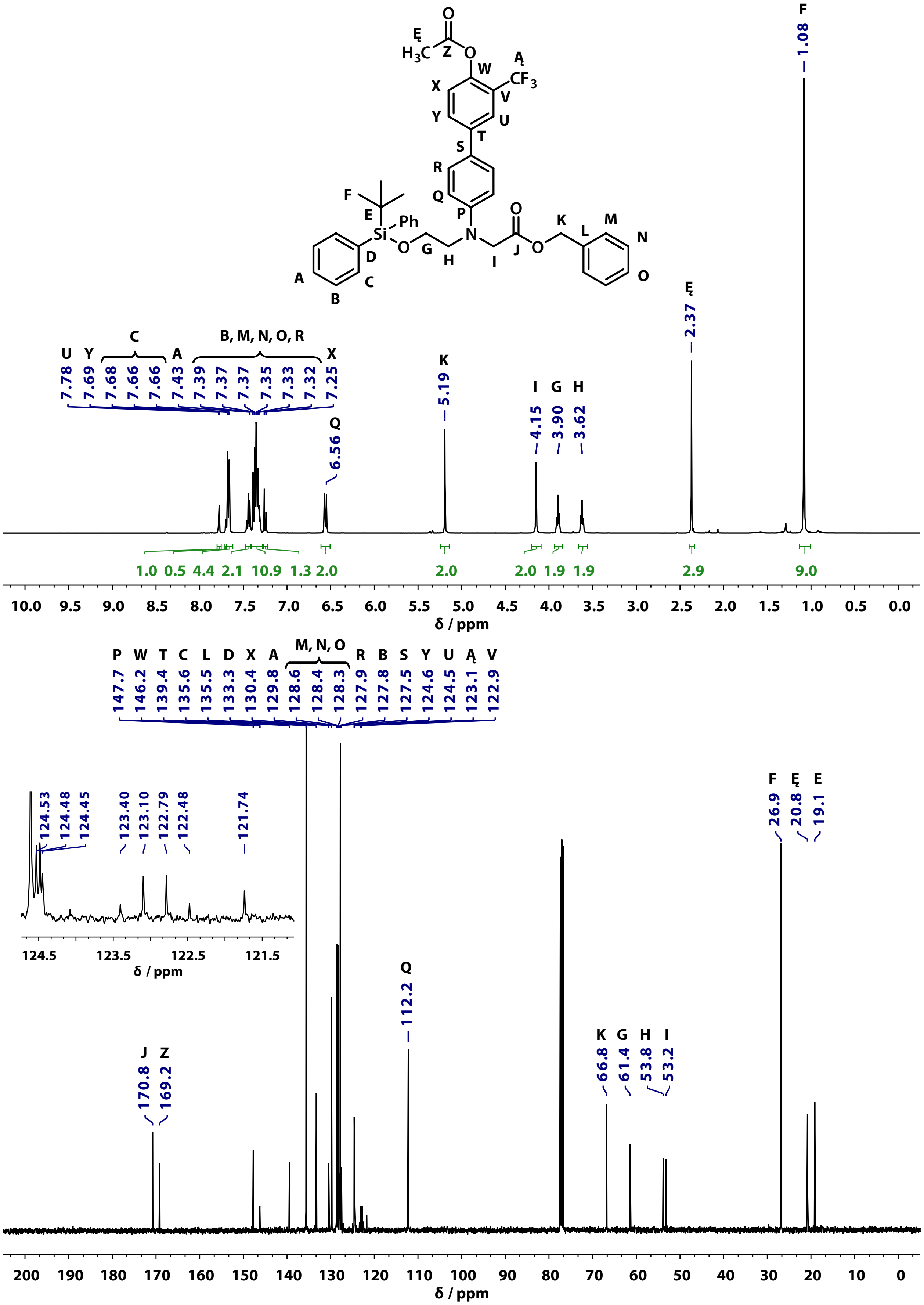

F
1.08
Ę
2.37
U Y C A B, M, N, O, R X
7.78 7.69 7.68 7.66 7.66 7.43 7.39 7.37 7.37 7.35 7.33 7.32 7.25
Q
6.56
K
5.19
I G H
4.15 3.90 3.62
1.0 0.5 4.4 2.1 10.9 1.3 2.0
2.0
2.0 1.9 1.9
2.9
9.0
10.0 9.5 9.0 8.5 8.0 7.5 7.0 6.5 6.0 5.5 5.0 4.5 4.0 3.5 3.0 2.5 2.0 1.5 1.0 0.5 0.0
δ / ppm
P W T C L D X A M, N, O R B S Y U Ą V
147.7 146.2 139.4 135.6 135.5 133.3 130.4 129.8 128.6 128.4 128.3 127.9 127.8 127.5 124.6 124.5 123.1 122.9
124.53 124.48 124.45 123.40 123.10 122.79 122.48 121.74
124.5 123.5 122.5 121.5
δ / ppm
F Ę E
26.9 20.8 19.1
Q
112.2
J Z
170.8 169.2
K G H I
66.8 61.4 53.8 53.2
200 190 180 170 160 150 140 130 120 110 100 90 80 70 60 50 40 30 20 10 0
δ / ppm

2.9 Synthesis of 12

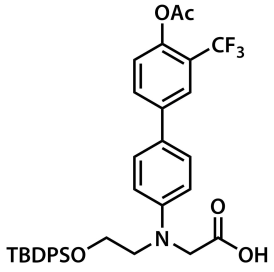

Nitrogen gas was bubbled for 15 min through a suspension of **11** (150 mg, 0.21 mmol) and palladium on carbon (22 mg, 10 wt% loading, 0.02 mmol) in absolute ethanol (10 ml). Hydrogen gas was then purged for 15 min through the suspension, which was subsequently left stirring under hydrogen atmosphere overnight. The reaction mixture was filtered through Celite, washed with ethanol (3 × 15 ml) and concentrated under vacuum to yield compound **12** as a white wax (97 mg, 0.2 mmol, 72%).

^{1}H NMR (400 MHz, $CDCl_3$) δ 10.32 (br s, 1H), 7.80 (br d, J = 2.2 Hz, 1H), 7.72 – 7.68 (m, 5H), 7.51 – 7.336 (m, 8H), 7.27 (br d, J = 9.3 Hz, 1H), 6.64 (br d, J = 8.3 Hz, 2H), 4.18 (s, 2H), 3.92 (br t, J = 5.9 Hz, 2H), 3.63 (br t, J = 5.9 Hz, 2H), 2.39 (s, 2H), 1.10 (s, 7H) ppm.

^{13}C NMR (101 MHz, $CDCl_3$) δ 176.3, 169.2, 147.4, 146.3 (br s), 139.2, 135.6, 133.0, 130.5, 129.9, 128.1, 128.0, 127.8, 124.7, 124.6 (q, J = 5 Hz), 123.1 (q, J = 273 Hz), 123.0 (q, J = 31 Hz), 112.6, 61.5, 53.8, 53.6, 26.8, 20.8, 19.1 ppm.

^{19}F NMR (376 MHz, $CDCl_3$) δ -62.3 ppm.

FT-IR (neat): 3045, 2955, 2931 (br), 2858, 1771, 1718, 1528 cm^{-1}.

HR-MS (ESI): m/z = 636.2402 $[M+H]^+$ (calcd. for $C_{35}H_{37}NO_5F_3Si$: 636.2393, Δ 1.4 ppm).

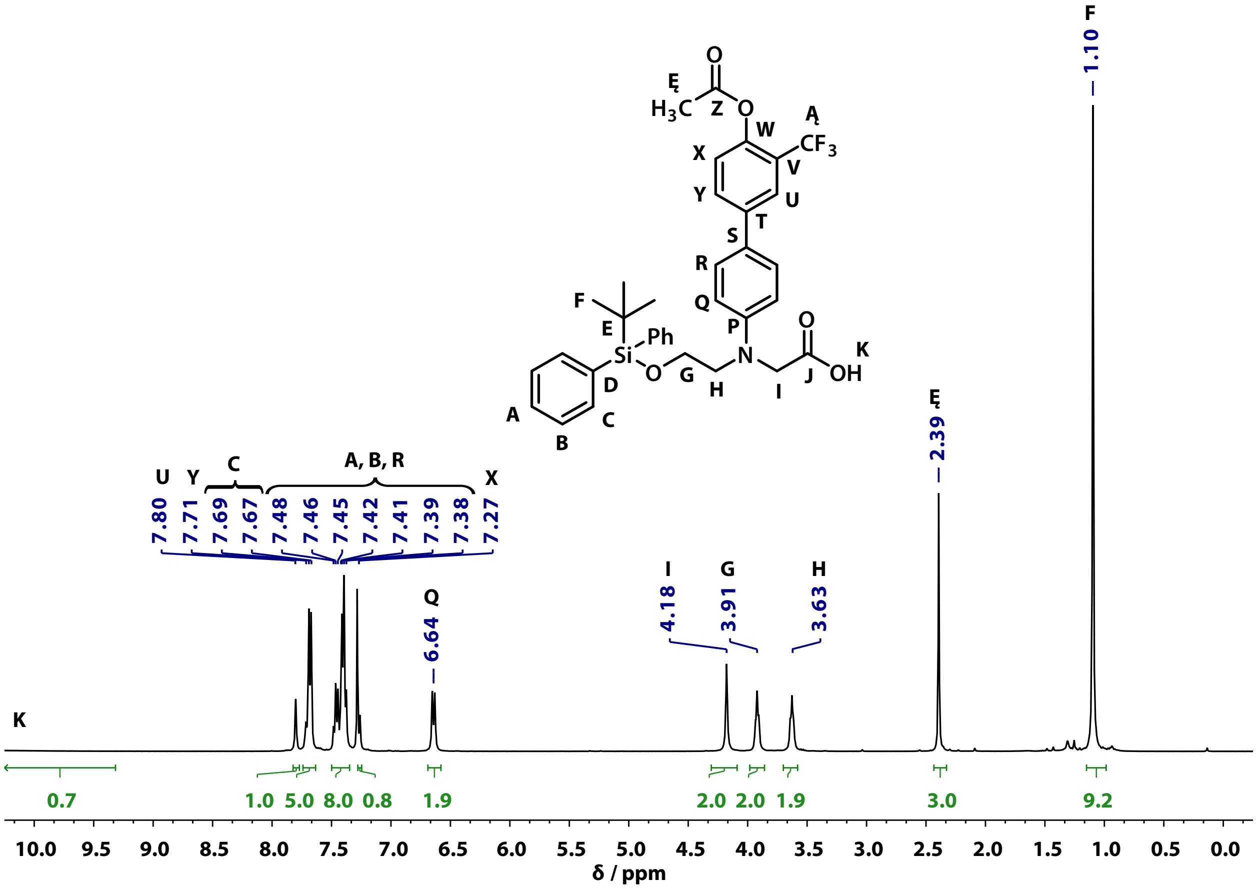
F
1.10
Ę
2.39
H3C Z O
O W
Ą CF3
X V
Y U
T
S
R
Q
P
F
E
Ph
Si
O G
N
H I
J
O
K
OH
D
A C
B
U Y C A, B, R X
7.80 7.71 7.69 7.67 7.48 7.46 7.45 7.42 7.41 7.39 7.38 7.27
Q
6.64
I G H
4.18 3.91 3.63
K
0.7 1.0 5.0 8.0 0.8 1.9 2.0 2.0 1.9 3.0 9.2
10.0 9.5 9.0 8.5 8.0 7.5 7.0 6.5 6.0 5.5 5.0 4.5 4.0 3.5 3.0 2.5 2.0 1.5 1.0 0.5 0.0
δ / ppm

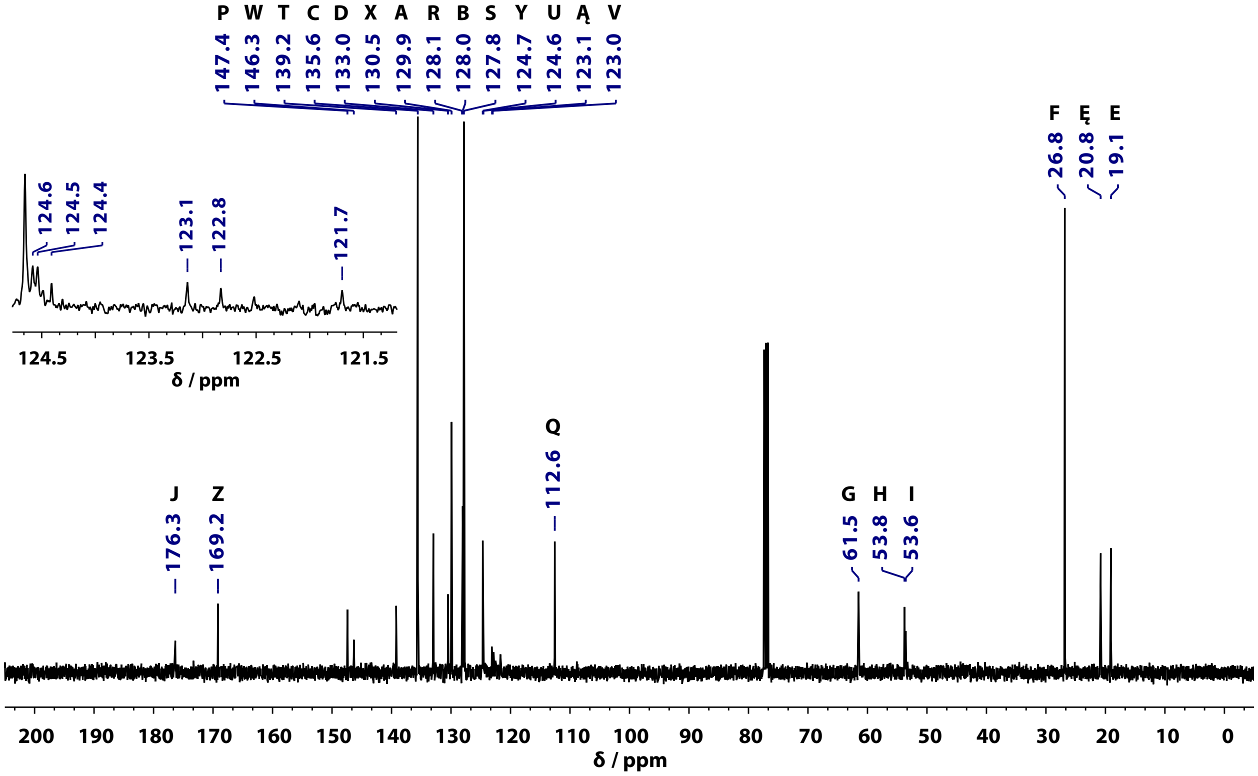
P W T C D X A R B S Y U Ą V
147.4 146.3 139.2 135.6 133.0 130.5 129.9 128.1 128.0 127.8 124.7 124.6 123.1 123.0
F Ę E
26.8 20.8 19.1
124.6 124.5 124.4 123.1 122.8 121.7
124.5 123.5 122.5 121.5
δ / ppm
Q
112.6
J Z
176.3 169.2
G H I
61.5 53.8 53.6
200 190 180 170 160 150 140 130 120 110 100 90 80 70 60 50 40 30 20 10 0
δ / ppm

2.10 Synthesis of 13

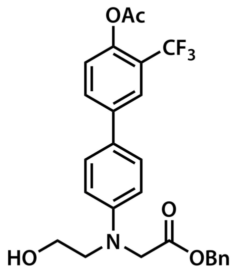

To a solution of compound **11** (60 mg, 0.08 mmol) in anhydrous tetrahydrofuran (5 ml) with acetic acid (38 µl, 0.7 mmol) was added *n*-tetrabutylammonium acetate (120 µl, 1 M in THF, 0.12 mmol). The solution was stirred under nitrogen atmosphere overnight. Upon completion, the reaction mixture was diluted with water (10 ml) and extracted with ethyl acetate (3 × 10 ml). The combined organic extracts were washed with brine (10 ml), dried with anhydrous magnesium sulfate, filtered and concentrated under vacuum. The residue was purified using column chromatography (0-50% ethyl acetate in petroleum ether) to yield product **13** as a white wax (35 mg, 0.07 mmol, 88%).

^{1}H NMR (400 MHz, $CDCl_3$) δ 7.77 (d, J = 2.2 Hz, 1H), 7.72 – 7.63 (dd, J = 8.5, 2.2 Hz, 1H), 7.41 (d, J = 8.8 Hz, 2H), 7.40 – 7.35 (m, 5H), 7.24 (d, J = 8.5 Hz, 1H), 6.68 (d, J = 8.8 Hz, 2H), 5.25 (s, 2H), 4.21 (s, 2H), 3.85 (br q, J = 5.2 Hz, 2H), 3.68 (br t, J = 4.8 Hz, 2H), 3.37 (br t, J = 6.6 Hz, 1H), 2.35 (s, 3H) ppm.

^{13}C NMR (101 MHz, $CDCl_3$) δ 172.8, 169.1, 147.1, 146.3 (br), 139.2, 135.0, 130.5, 127.7, 128.7, 128.6, 128.1, 128.1, 124.6, 124.6 (d, J = 5 Hz), 123.0 (d, J = 31 Hz), 123.0 (d, J = 273 Hz), 112.4, 67.6, 60.1, 55.2, 54.4, 20.8 ppm.

^{19}F NMR (376 MHz, $CDCl_3$) δ -61.9 ppm.

FT-IR (neat): 3443 (br), 3036, 2948, 2879, 1769, 1731, 1610 cm^{-1}.

HR-MS (ESI): m/z = 488.1672 $[M+H]^+$ (calcd. for $C_{26}H_{25}O_5NF_3$: 488.1679, Δ -1.59 ppm).

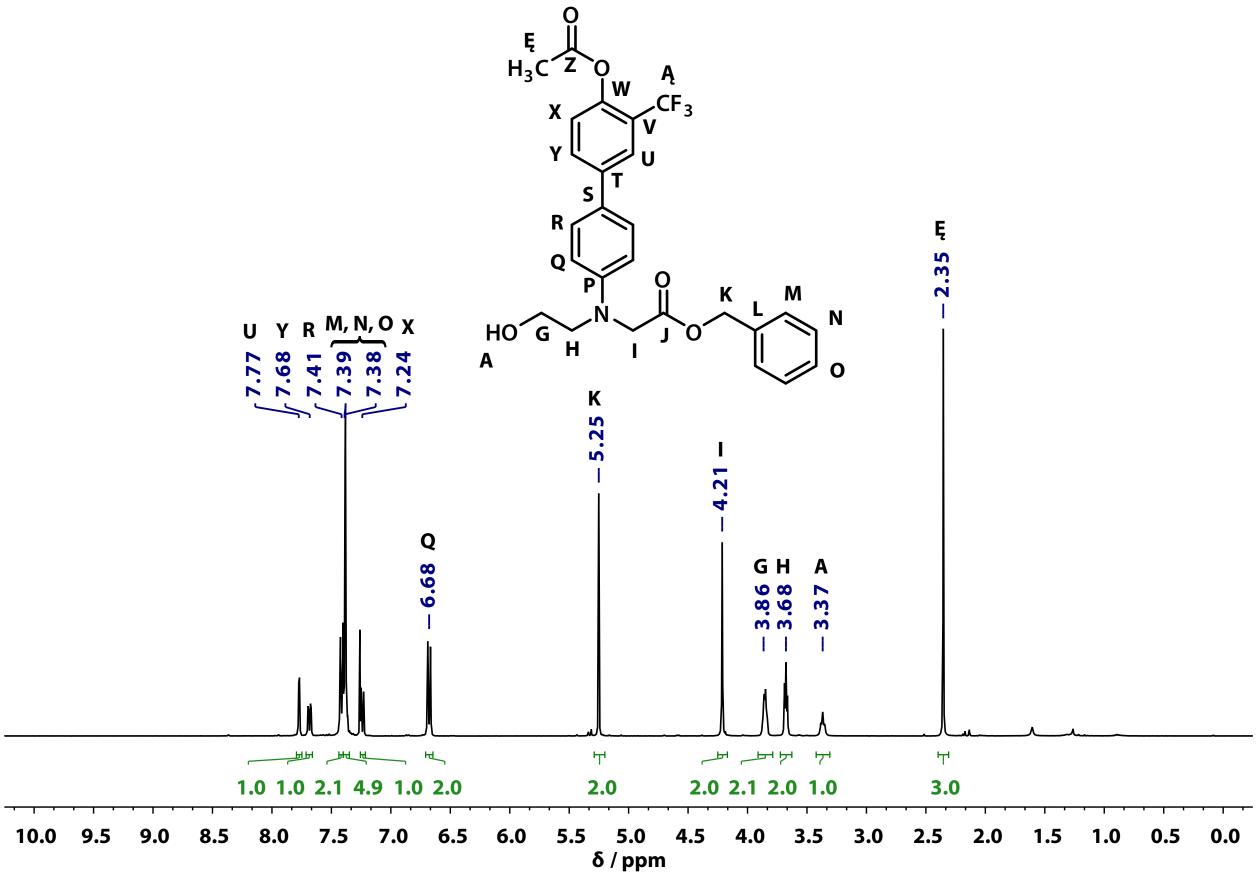
O
Ę
H_3C Z O
Ą
W CF_3
X V
Y U
T
S
R
Q
P
O
K M
L
N
HO G N J O
A H I
O
U Y R M, N, O X
7.77 7.68 7.41 7.39 7.38 7.24
Q
6.68
K
5.25
I
4.21
G H A
3.86 3.68 3.37
Ę
2.35
1.0 1.0 2.1 4.9 1.0 2.0
2.0
2.0 2.1 2.0 1.0
3.0
10.0 9.5 9.0 8.5 8.0 7.5 7.0 6.5 6.0 5.5 5.0 4.5 4.0 3.5 3.0 2.5 2.0 1.5 1.0 0.5 0.0
δ / ppm

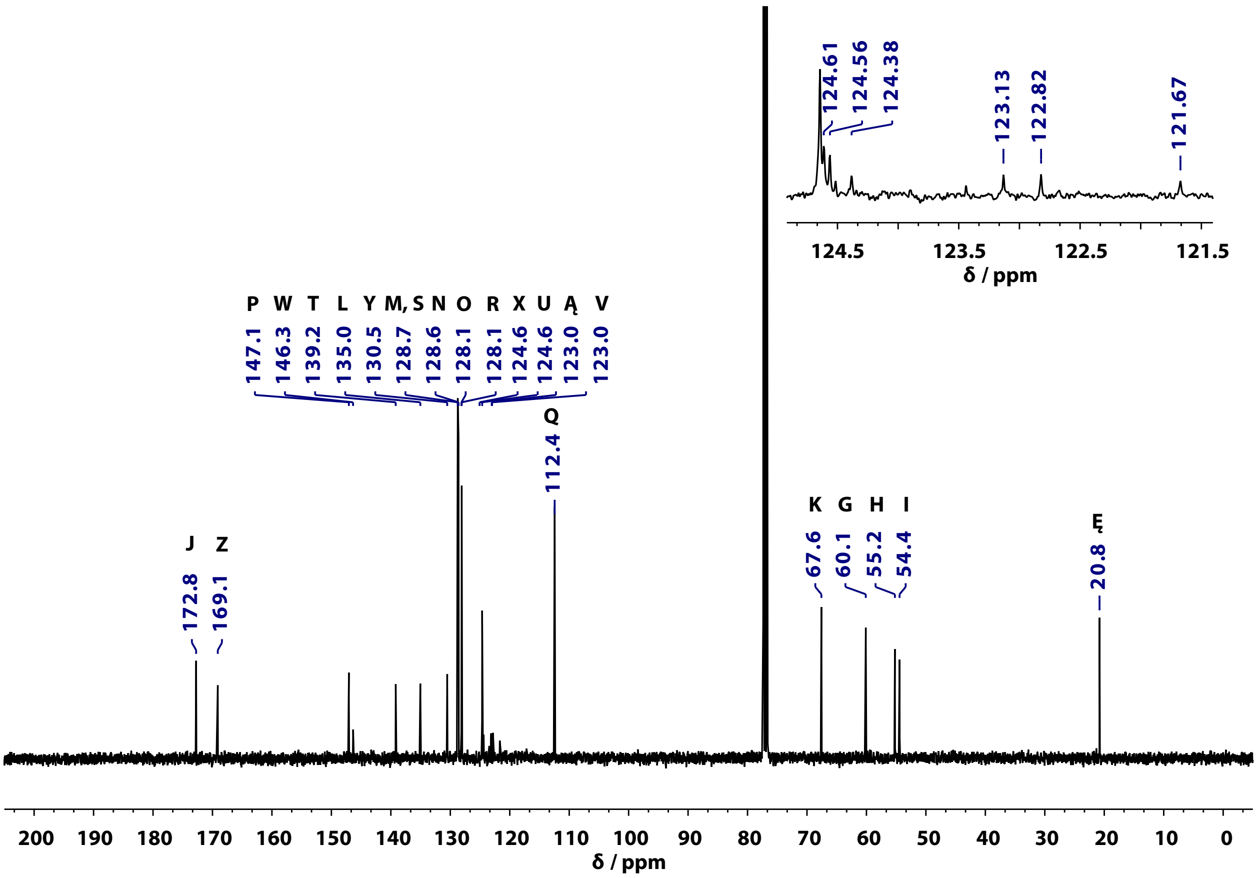
124.61 124.56 124.38
123.13 122.82
121.67
124.5 123.5 122.5 121.5
δ / ppm
P W T L Y M, S N O R X U Ą V
147.1 146.3 139.2 135.0 130.5 128.7 128.6 128.1 128.1 124.6 124.6 123.0 123.0
Q
112.4
J Z
172.8 169.1
K G H I
67.6 60.1 55.2 54.4
Ę
20.8
200 190 180 170 160 150 140 130 120 110 100 90 80 70 60 50 40 30 20 10 0
δ / ppm

2.11 Synthesis of 14

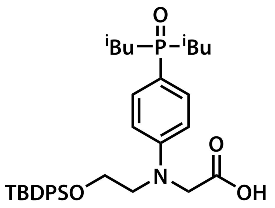

Nitrogen gas was bubbled for 15 min through a suspension of **10** (640 mg, 0.9 mmol) and palladium on carbon (155 mg, 10 wt% loading, 0.15 mmol) in absolute ethanol (15 ml). Hydrogen gas was then purged for 15 min through the suspension, which was subsequently left stirring under hydrogen atmosphere overnight. The reaction mixture was filtered through Celite, washed with ethanol (3 × 30 ml) and concentrated under vacuum to yield compound **14** as a white powder (389 mg, 0.66 mmol, 73%).

^{1}H NMR (400 MHz, $CDCl_3$) δ 7.67 – 7.59 (m, 4H), 7.45 – 7.30 (m, 8H), 6.55 (d, J = 6.7 Hz, 2H), 3.94 (s, 2H), 3.84 (t, J = 6.1 Hz, 2H), 3.60 (t, J = 6.1 Hz, 2H), 2.04 – 1.90 (m, 2H), 1.89 – 1.77 (m, 2H), 1.78 – 1.66 (m, 2H), 1.02 (s, 9H), 1.01 (d, J = 6.7 Hz, 6H), 0.87 (d, J = 6.6 Hz, 6H) ppm.

^{13}C NMR (101 MHz, $CDCl_3$) δ 171.6 (br), 150.6, 135.2, 132.9, 131.2 (d, J = 10 Hz), 129.4, 127.4, 116.3 (d, J = 103 Hz), 111.3 (d, J = 12 Hz), 61.0, 53.3, 52.3, 39.0 (d, J = 67 Hz), 26.5, 24.3 (d, J = 9 Hz), 24.2 (d, J = 8 Hz), 23.0 (d, J = 4 Hz), 18.7 ppm.

^{31}P NMR(162 MHz, $CDCl_3$) δ 45.0 ppm.

FT-IR (neat): 2953, 2929 (br), 2869, 1931 (br), 1598 cm^{-1}.

HR-MS (ESI): m/z = 594.3193 $[M+H]^+$ (calcd. for $C_{34}H_{49}NO_4PSi$: 594.3168, Δ 4.2 ppm).

mp. 192 °C.

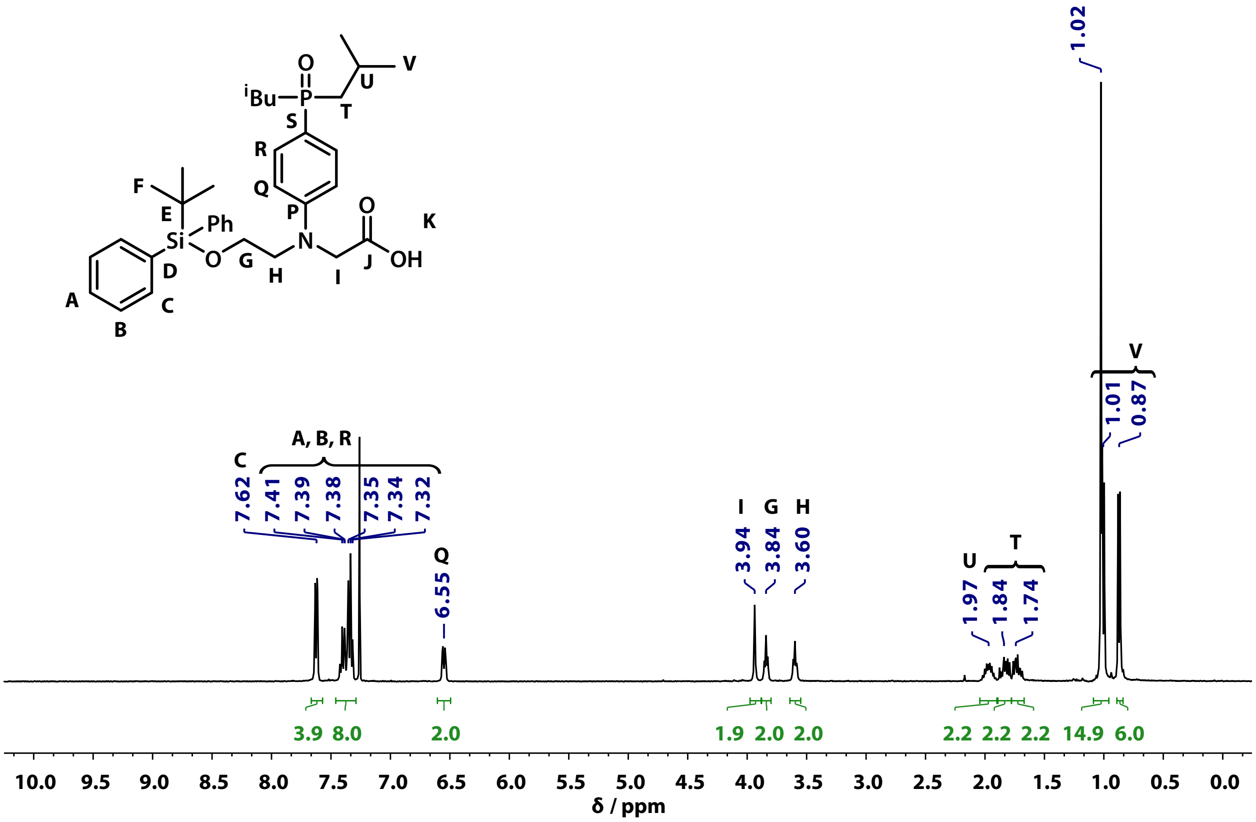

O
^{i}Bu
P
S
T
U
V
R
Q
P
F
E
Ph
Si
O
G
H
N
I
J
O
OH
K
D
A
C
B
C
A, B, R
7.62
7.41
7.39
7.38
7.35
7.34
7.32
Q
6.55
I G H
3.94
3.84
3.60
U
T
1.97
1.84
1.74
1.02
V
1.01
0.87
3.9 8.0 2.0 1.9 2.0 2.0 2.2 2.2 2.2 14.9 6.0
10.0 9.5 9.0 8.5 8.0 7.5 7.0 6.5 6.0 5.5 5.0 4.5 4.0 3.5 3.0 2.5 2.0 1.5 1.0 0.5 0.0
δ / ppm

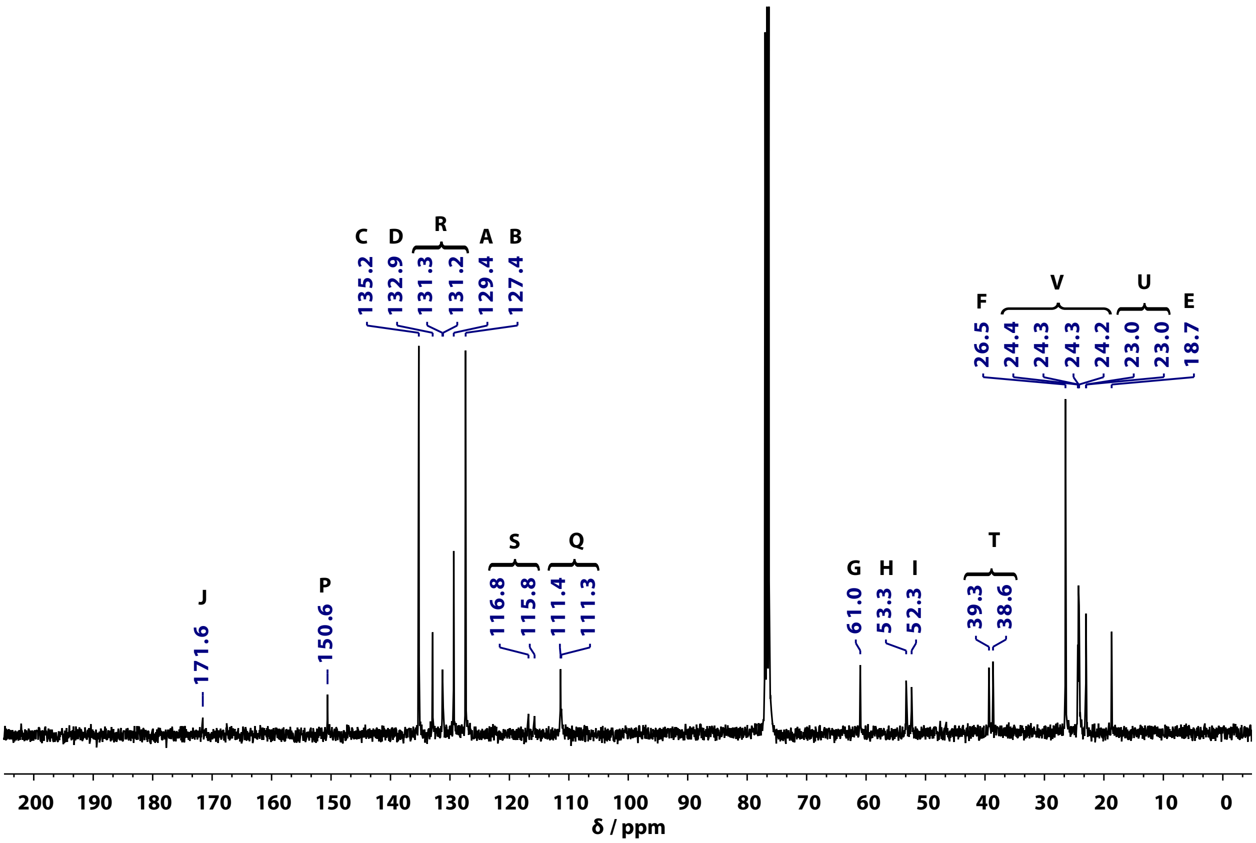

C D R A B
135.2
132.9
131.3
131.2
129.4
127.4
F V U E
26.5
24.4
24.3
24.3
24.2
23.0
23.0
18.7
J
171.6
P
150.6
S Q
116.8
115.8
111.4
111.3
G H I
61.0
53.3
52.3
T
39.3
38.6
200 190 180 170 160 150 140 130 120 110 100 90 80 70 60 50 40 30 20 10 0
δ / ppm

2.12 Synthesis of 15

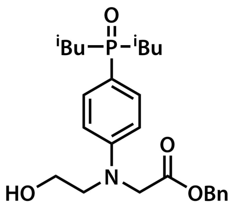

To a solution of compound **10** (358 mg, 0.5 mmol) in anhydrous tetrahydrofuran (5 ml) with acetic acid (335 µl, 5.9 mmol) was added *n*-tetrabutylammonium acetate (735 µl, 1 M in THF, 0.74 mmol). The solution was stirred under nitrogen atmosphere overnight. Upon completion, the reaction mixture was diluted with water (10 ml) and extracted with ethyl acetate (3 × 10 ml). The combined organic extracts were washed with brine (10 ml), dried with anhydrous magnesium sulfate, filtered and concentrated under vacuum. The residue was purified using column chromatography (0-10% methanol in dichloromethane) to yield product **15** as a colourless oil (167 mg, 0.34 mmol, 72%).

^{1}H NMR (400 MHz, $CDCl_3$) δ 7.53 – 7.43 (m, 2H), 7.43 – 7.31 (m, 6H), 6.67 (d, J = 6.7 Hz, 2H), 5.23 (s, 2H), 4.22 (s, 2H), 3.85 (t, J = 5.1 Hz, 2H), 3.67 (t, J = 5.0 Hz, 2H), 2.05 – 1.91 (m, 2H), 1.91 – 1.78 (m, 2H), 1.73 – 1.63 (m, 2H), 1.04 (d, J = 6.6 Hz, 6H), 0.87 (d, J = 6.6 Hz, 6H) ppm.

^{13}C NMR (101 MHz, $CDCl_3$) δ 172.1, 149.6, 135.0, 131.9 (d, J = 10 Hz), 128.7, 128.7, 128.5, 119.9 (d, J = 100 Hz), 111.7 (d, J = 12 Hz), 67.5, 59.8, 54.9, 54.0, 40.0 (d, J = 69 Hz), 24.8 (d, J = 9 Hz), 24.6 (d, J = 8 Hz), 23.4 (d, J = 4 Hz) ppm.

^{31}P NMR(162 MHz, $CDCl_3$) δ 39.9 ppm.

FT-IR (neat): 3288 (br), 2954, 2926, 2870, 1743, 1597, 1513 cm^{-1}.

HR-MS (ESI): m/z = 446.2453 $[M+H]^+$ (calcd. for $C_{25}H_{37}NO_4P$: 446.2460, Δ -1.6 ppm).

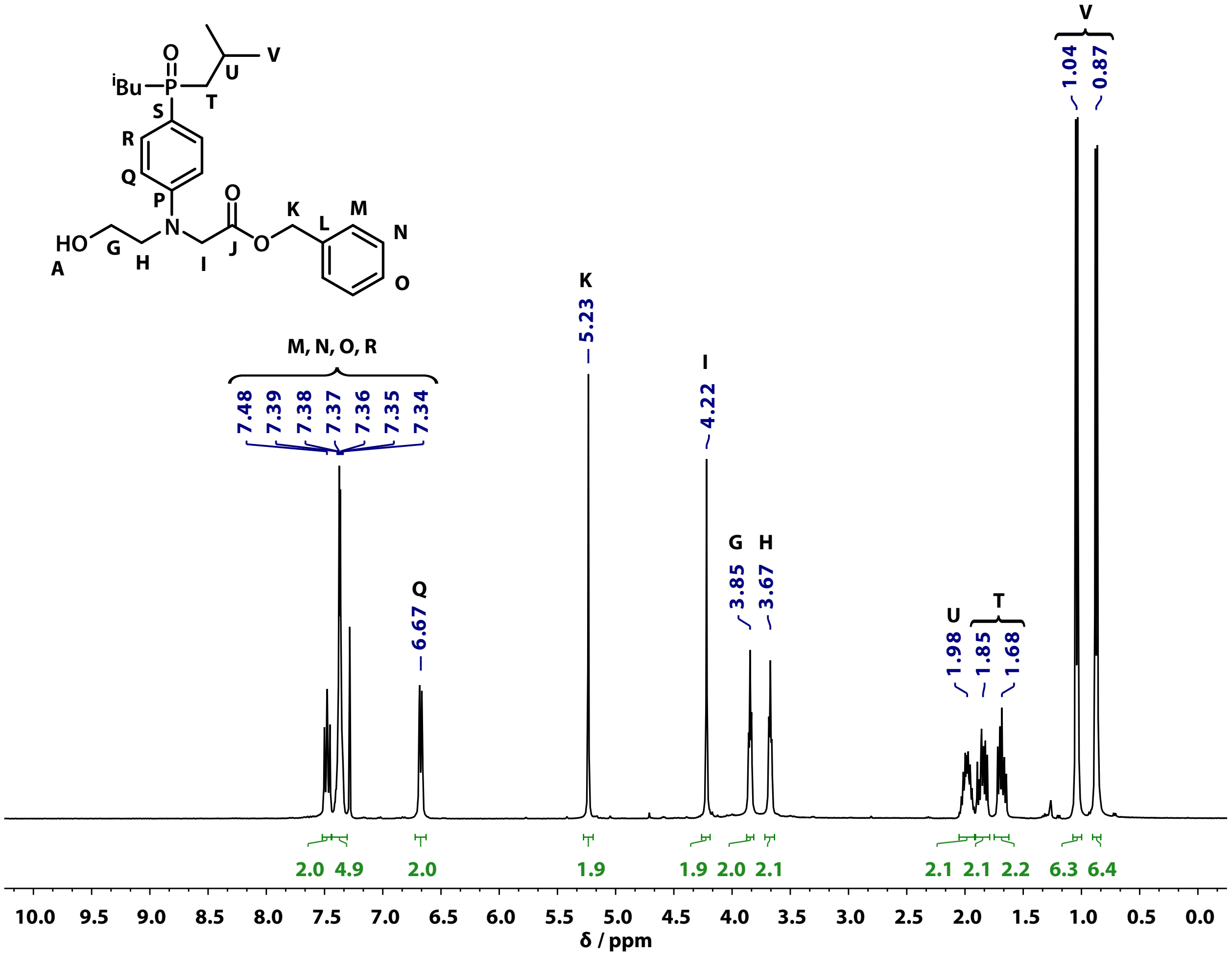

O
iBu
P
U
V
T
S
R
Q
P
N
O
K
L
M
HO
G
H
I
J
A
N
O
M, N, O, R
7.48
7.39
7.38
7.37
7.36
7.35
7.34
Q
6.67
K
5.23
I
4.22
G
H
3.85
3.67
U
T
1.98
1.85
1.68
V
1.04
0.87
2.0
4.9
2.0
1.9
1.9
2.0
2.1
2.1
2.1
2.2
6.3
6.4
10.0
9.5
9.0
8.5
8.0
7.5
7.0
6.5
6.0
5.5
5.0
4.5
4.0
3.5
3.0
2.5
2.0
1.5
1.0
0.5
0.0
δ / ppm

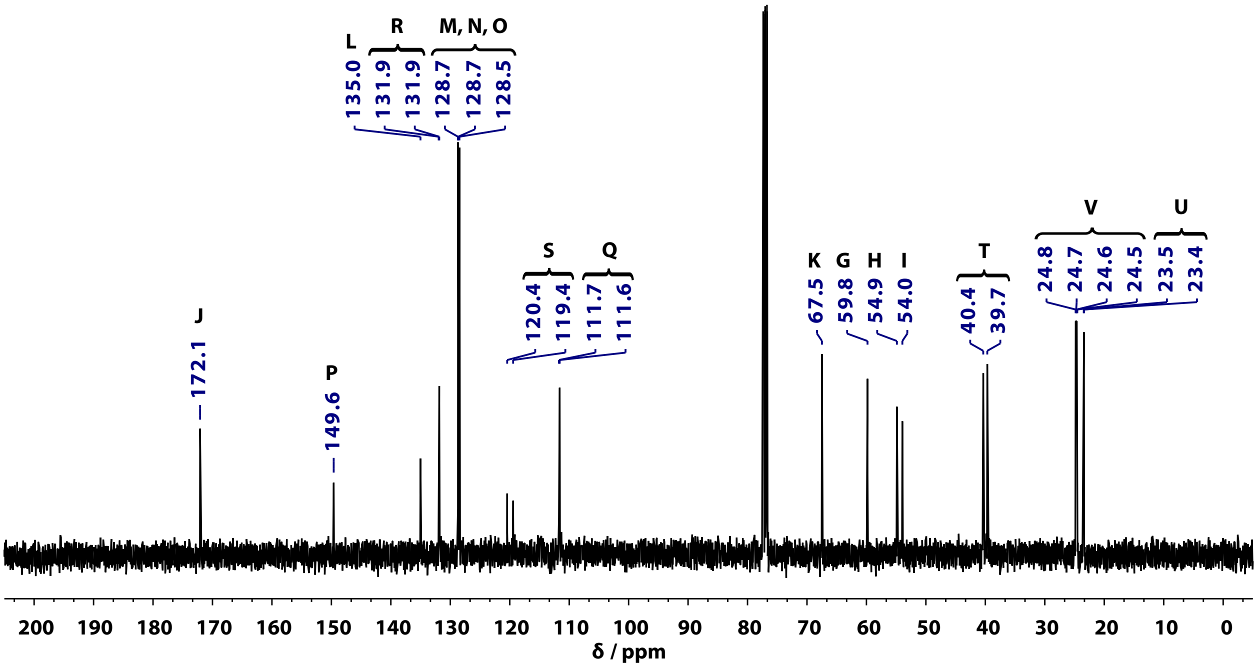

L
R
M, N, O
135.0
131.9
131.9
128.7
128.7
128.5
J
172.1
P
149.6
S
Q
120.4
119.4
111.7
111.6
K
G
H
I
67.5
59.8
54.9
54.0
T
40.4
39.7
V
24.8
24.7
24.6
24.5
U
23.5
23.4
200
190
180
170
160
150
140
130
120
110
100
90
80
70
60
50
40
30
20
10
0
δ / ppm

2.13 Synthesis of 16

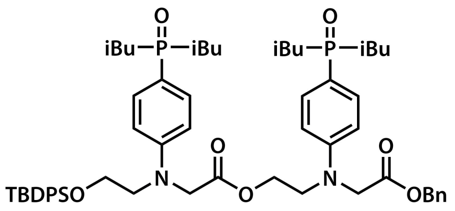

A solution of compounds **14** (107 mg, 0.2 mmol) and **15** (80 mg, 0.2 mmol) in anhydrous dichloromethane (5 ml) with *N,N*-dimethylaminopyridine (2 mg, 0.02 mmol) and EDC·HCl (41 mg, 0.2 mmol) was stirred overnight under nitrogen atmosphere. Upon completion, the reaction mixture was poured into water (20 ml). The layers were separated and the aqueous layer was subsequently washed with dichloromethane (3 × 15 ml). The combined organic extracts were washed with brine (20 ml). The washed solution was dried with anhydrous magnesium sulfate, filtered and concentrated under vacuum. The residue was purified using column chromatography (0-10% methanol in dichloromethane) to yield compound **16** as a white wax (135 mg, 0.15 mmol, 73%).

^{1}H NMR (400 MHz, $CDCl_3$) δ 7.49 (m, 4H), 7.43 – 7.24 (m, 15H), 6.70 – 6.64 (m, 2H), 6.53 – 6.46 (m, 2H), 5.16 (s, 2H), 4.33 (t, J = 6.2 Hz, 2H), 4.06 (s, 2H), 4.02 (s, 2H), 3.82 (t, J = 5.8 Hz, 2H), 3.67 (t, J = 6.2 Hz, 2H), 3.53 (t, J = 5.8 Hz, 2H), 2.05 – 1.90 (m, 4H), 1.90 – 1.76 (m, 4H), 1.74 – 1.57 (m, 4H), 1.03 – 1.02 (m, 21H), 0.84 (d, J = 6.5 Hz, 12H) ppm.

^{13}C NMR (101 MHz, $CDCl_3$) δ 170.5, 170.1, 149.8 (br), 149.4 (br), 135.5, 135.2, 133.1, 132.0 (d, J = 10 Hz), 131.9 (d, J = 9 Hz), 129.8, 128.7, 128.6, 128.3, 127.8, 119.8 (d, J = 99 Hz), 111.6 (d, J = 12 Hz), 111.4 (d, J = 12 Hz), 67.1, 62.2, 61.4, 53.4, 52.7, 52.6, 50.1, 40.1 (d, J = 68 Hz), 26.8, 24.8 (d, J = 9 Hz), 24.6 (d, J = 8 Hz), 23.4, 23.4, 19.0 ppm.

^{31}P NMR(162 MHz, $CDCl_3$) δ 38.8, 38.7 ppm.

FT-IR (neat): 3349 (br), 2954, 2929, 2869, 1745 (br), 1598, 1513 cm^{-1}.

HR-MS (ESI): m/z = 1021.5407 $[M+H]^+$ (calcd. for $C_{59}H_{83}N_2O_7P_2Si$: 1021.5445, Δ 3.7 ppm)
m/z = 511.2696 $[M+2H]^{2+}$ (calcd. for $C_{59}H_{84}N_2O_7P_2Si$: 1021.5445, Δ 11.7 ppm).

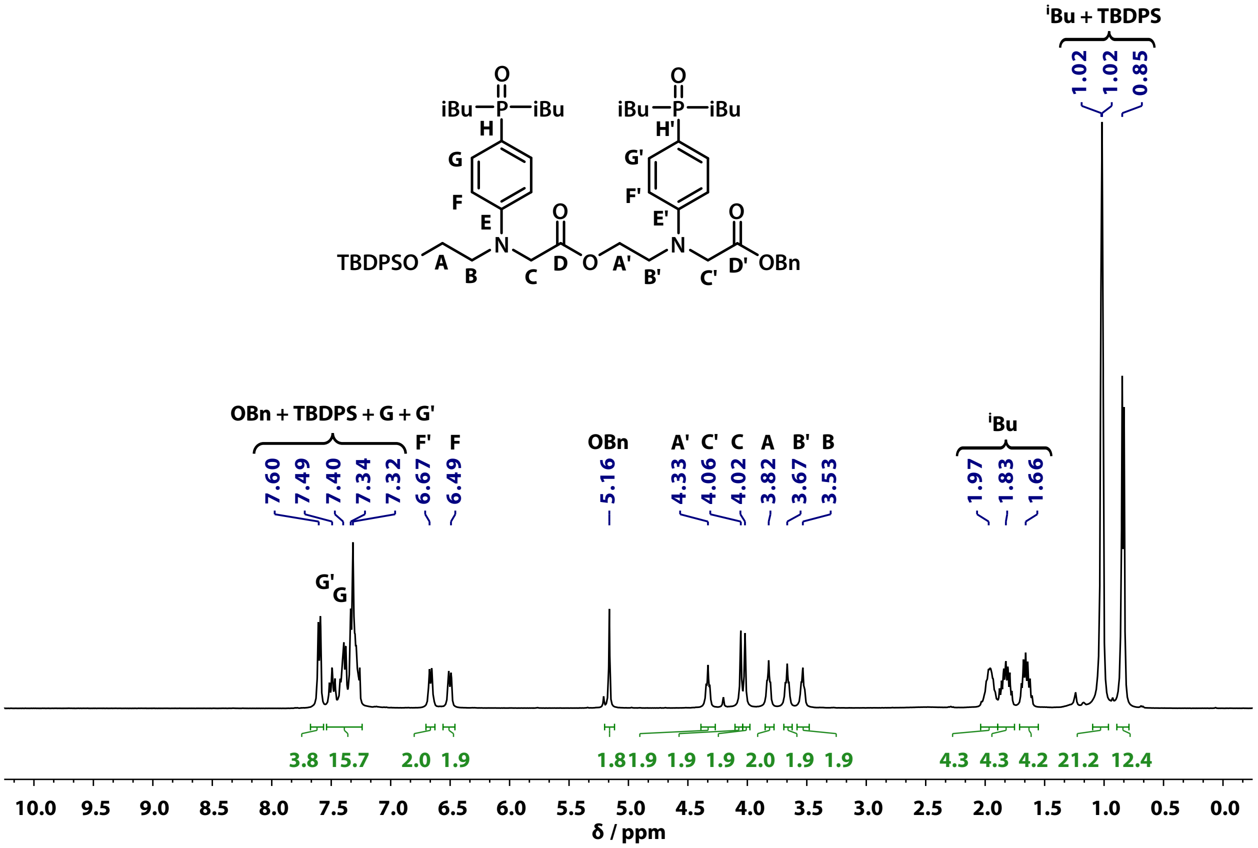
TBDPSO A B C D O A' B' C' D' OBn
iBu P iBu
O
H G F E N
H' G' F' E'
iBu + TBDPS
1.02 1.02 0.85
OBn + TBDPS + G + G'
7.60 7.49 7.40 7.34 7.32
F' F
6.67 6.49
OBn
5.16
A' C' C A B' B
4.33 4.06 4.02 3.82 3.67 3.53
iBu
1.97 1.83 1.66
G' G
3.8 15.7 2.0 1.9 1.81.9 1.9 1.9 2.0 1.9 1.9 4.3 4.3 4.2 21.2 12.4
10.0 9.5 9.0 8.5 8.0 7.5 7.0 6.5 6.0 5.5 5.0 4.5 4.0 3.5 3.0 2.5 2.0 1.5 1.0 0.5 0.0
δ / ppm

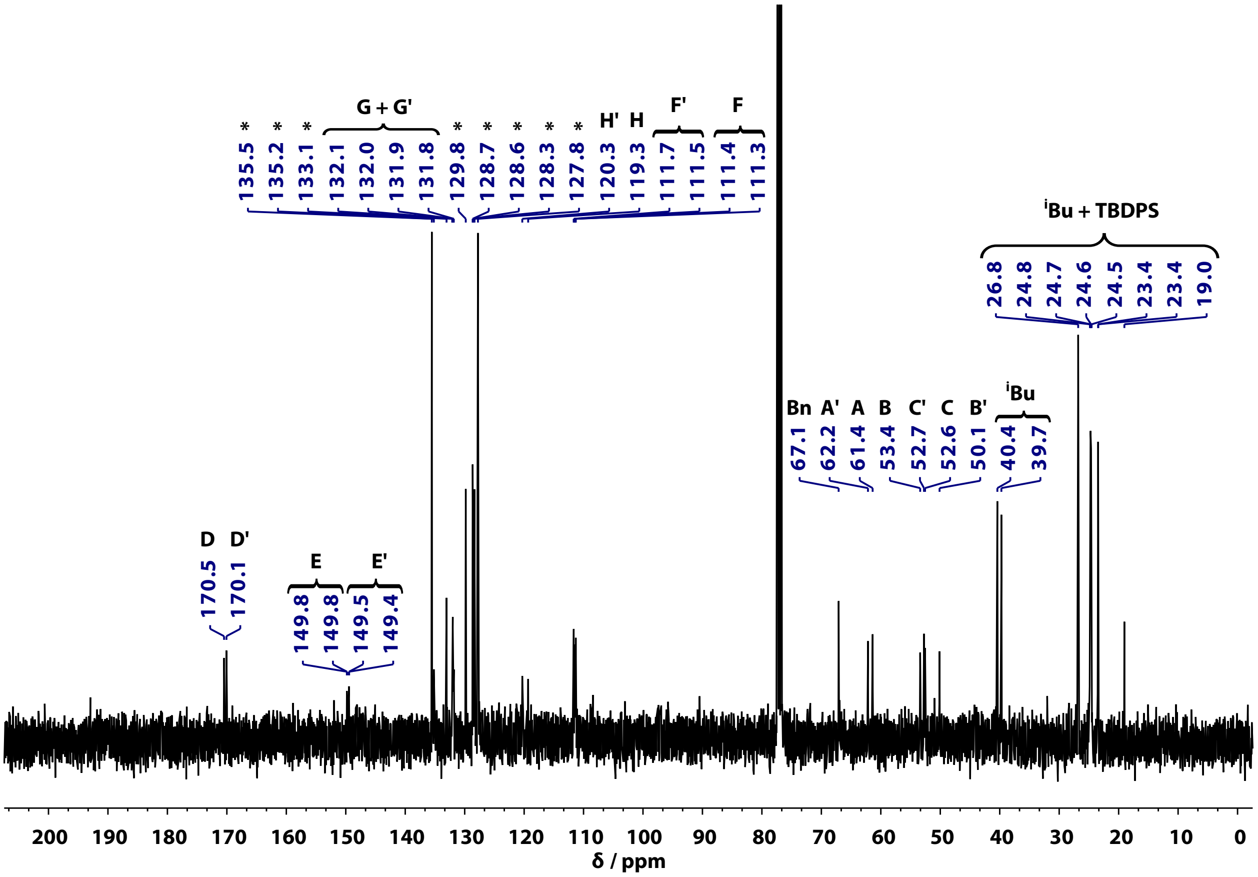
* * * G + G' * * * * * H' H F' F
135.5 135.2 133.1 132.1 132.0 131.9 131.8 129.8 128.7 128.6 128.3 127.8 120.3 119.3 111.7 111.5 111.4 111.3
iBu + TBDPS
26.8 24.8 24.7 24.6 24.5 23.4 23.4 19.0
Bn A' A B C' C B'
67.1 62.2 61.4 53.4 52.7 52.6 50.1
iBu
40.4 39.7
D D'
170.5 170.1
E E'
149.8 149.8 149.5 149.4
200 190 180 170 160 150 140 130 120 110 100 90 80 70 60 50 40 30 20 10 0
δ / ppm

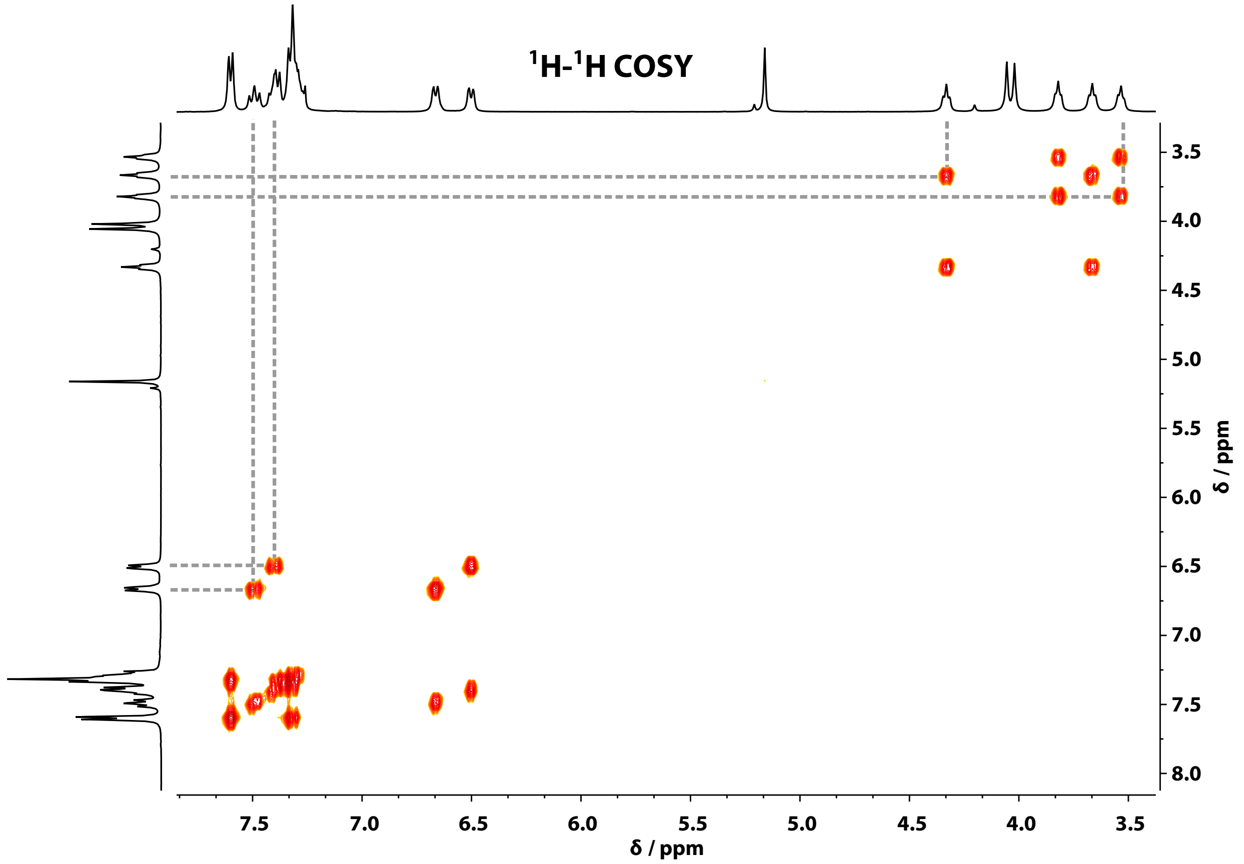

^{1}H-^{1}H COSY
δ / ppm
3.5
4.0
4.5
5.0
5.5
6.0
6.5
7.0
7.5
8.0

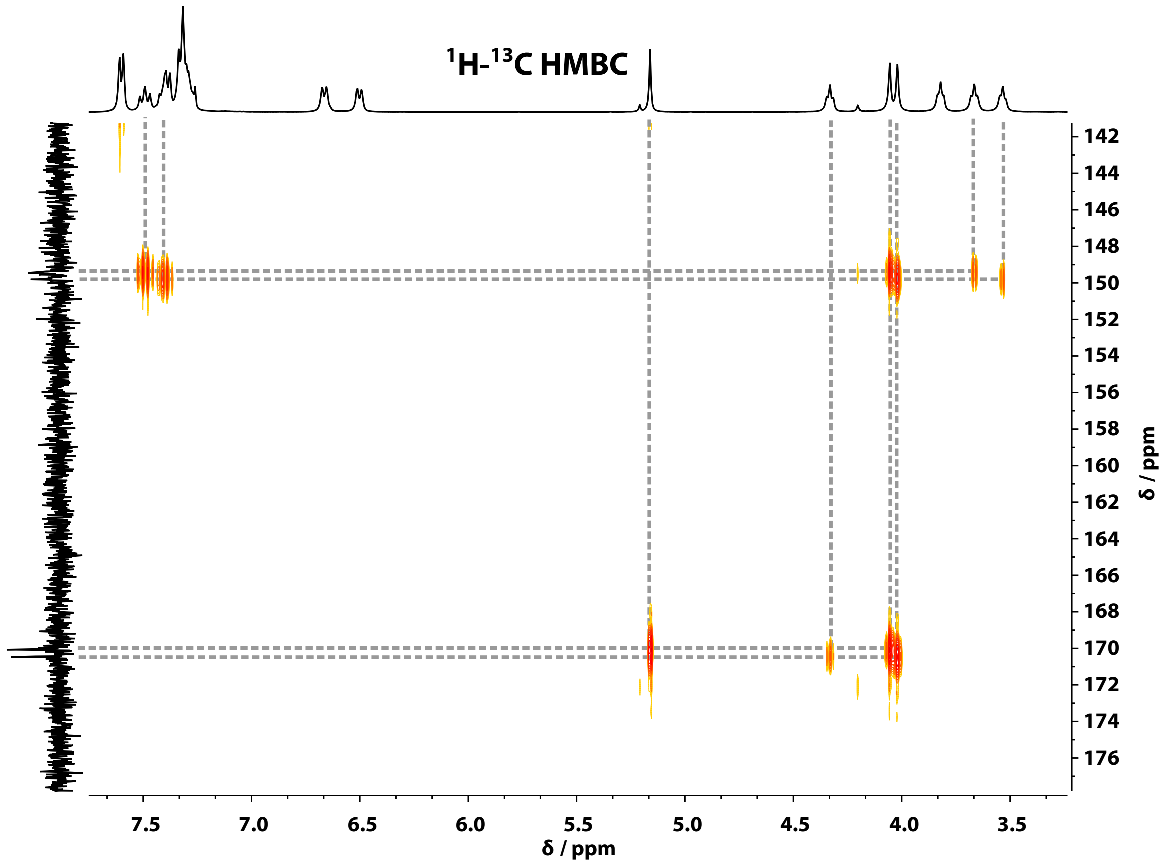

^{1}H-^{13}C HMBC
δ / ppm
142
144
146
148
150
152
154
156
158
160
162
164
166
168
170
172
174
176
7.5
7.0
6.5
6.0
5.5
5.0
4.5
4.0
3.5

2.14 Synthesis of 17

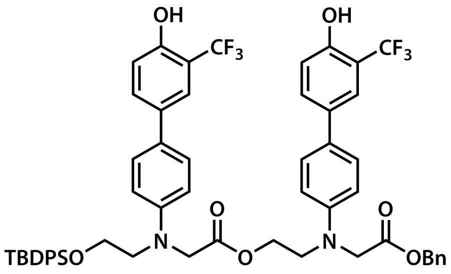

Compounds **12** (45 mg, 0.07 mmol) and **13** (35 mg, 0.07 mmol) in anhydrous dichloromethane (5 ml) with *N,N*-dimethylaminopyridine (1 mg, 0.01 mmol) and EDC·HCl (16 mg, 0.09 mmol) was stirred overnight under nitrogen atmosphere. The reaction mixture was poured into water (10 ml) and the layers were separated. The aqueous layer was subsequently washed with dichloromethane (3 × 5 ml). The combined organic extracts were washed with brine (20 ml). The washed solution was dried with anhydrous magnesium sulfate, filtered and concentrated under vacuum. The residue was purified using column chromatography (0-30% ethyl acetate in petroleum ether) to yield **17**-acetate as a white wax (37 mg, 0.03 mmol).

The intermediate (13 mg, 0.01 mmol) and ammonium acetate (7 mg, 0.1 mmol) were dissolved in a mixture of methanol (0.75 ml) and water (0.25 ml). The reaction mixture was stirred under nitrogen atmosphere overnight. After completion, the mixture was partitioned between water (5 ml) and ethyl acetate (5 ml). The layers were separated and the aqueous layer was subsequently washed with dichloromethane (2 × 5 ml). The combined organic extracts were washed with brine (10 ml). The washed solution was dried with anhydrous magnesium sulfate, filtered and concentrated under vacuum to yield compound **17** as a colourless oil (10 mg, 0.01 mmol, 60% over two steps).

^{1}H NMR (400 MHz, $CDCl_3$) δ 7.69 – 7.30 (m, 21H), 7.24 (d, J = 8.9 Hz, 2H), 6.93 (d, J = 8.7 Hz, 2H), 6.67 (d, J = 8.4 Hz, 2H), 6.48 (d, J = 8.4 Hz, 2H), 5.57 (s, 1H), 5.52 (s, 1H), 5.17 (s, 2H), 4.39 (br t,2 H), 4.08 (s, 2H), 4.03 (s, 2H), 3.85 (br t, 2H), 3.71 (br t, 2H), 3.56 (br t, 2H), 1.06 (s, 9H) ppm.

^{13}C NMR (101 MHz, $CDCl_3$) δ 171.2, 170.6, 151.9, 151.7, 146.5, 146.2, 135.2, 133.3, 132.9, 132.8, 130.6 (br), 129.4, 128.5, 128.3, 128.1, 128.0, 127.7, 127.4, 127.2, 127.1, 123.8 (q, J = 273 Hz), 123.8 (q, J = 273 Hz), 123.6 (q, J = 5 Hz), 123.6 (q, J = 5 Hz), 117.6, 117.6, 116.2 (q, J = 30 Hz), 116.2 (q, J = 30 Hz), 112.1, 111.7, 66.7, 62.1, 61.1, 53.4, 52.7, 52.4, 50.0, 26.5, 18.7 ppm.

^{19}F NMR (376 MHz, $CDCl_3$) δ -60.8, -60.8 ppm.

FT-IR (neat): 3380 (br), 2954, 2931, 2859, 1747 (br), 1610, 1528 cm^{-1}.

HR-MS (ESI): m/z = 1021.3713 $[M+H]^+$ (calcd. for $C_{57}H_{55}F_6N_2O_7Si$: 1021.3677, Δ 3.0 ppm).

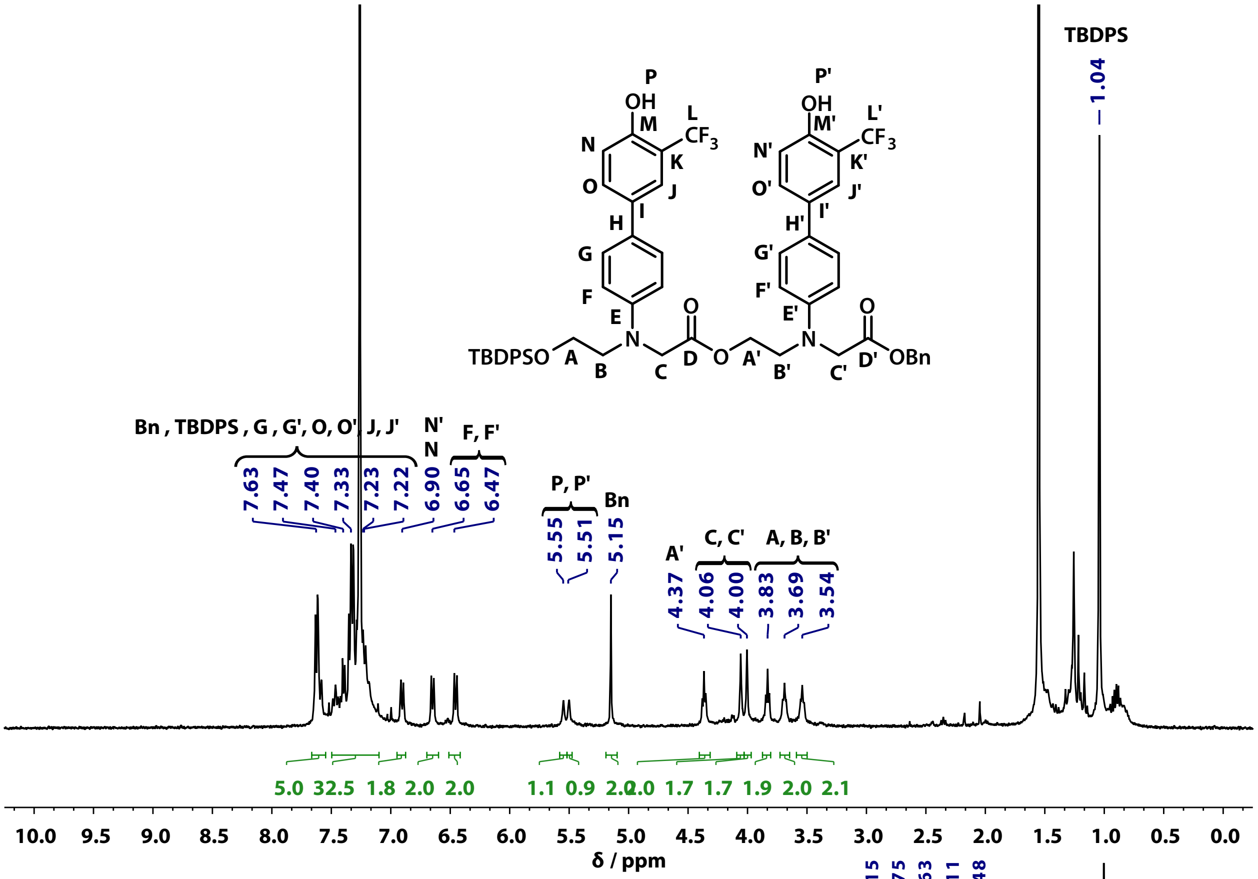
TBDPS
1.04
P
OH
M
L
CF3
N
O
K
J
I
H
G
F
E
P'
M'
L'
N'
O'
K'
J'
I'
H'
G'
F'
E'
TBDPSO A B N C D O A' B' N C' D' OBn
Bn , TBDPS , G , G', O, O', J, J'
7.63 7.47 7.40 7.33 7.23 7.22
N'
N
6.90
F, F'
6.65 6.47
P, P'
5.55 5.51
Bn
5.15
A'
4.37
C, C'
4.06 4.00
A, B, B'
3.83 3.69 3.54
5.0 32.5 1.8 2.0 2.0 1.1 0.9 2.0 2.0 1.7 1.7 1.9 2.0 2.1
10.0 9.5 9.0 8.5 8.0 7.5 7.0 6.5 6.0 5.5 5.0 4.5 4.0 3.5 3.0 2.5 2.0 1.5 1.0 0.5 0.0
δ / ppm

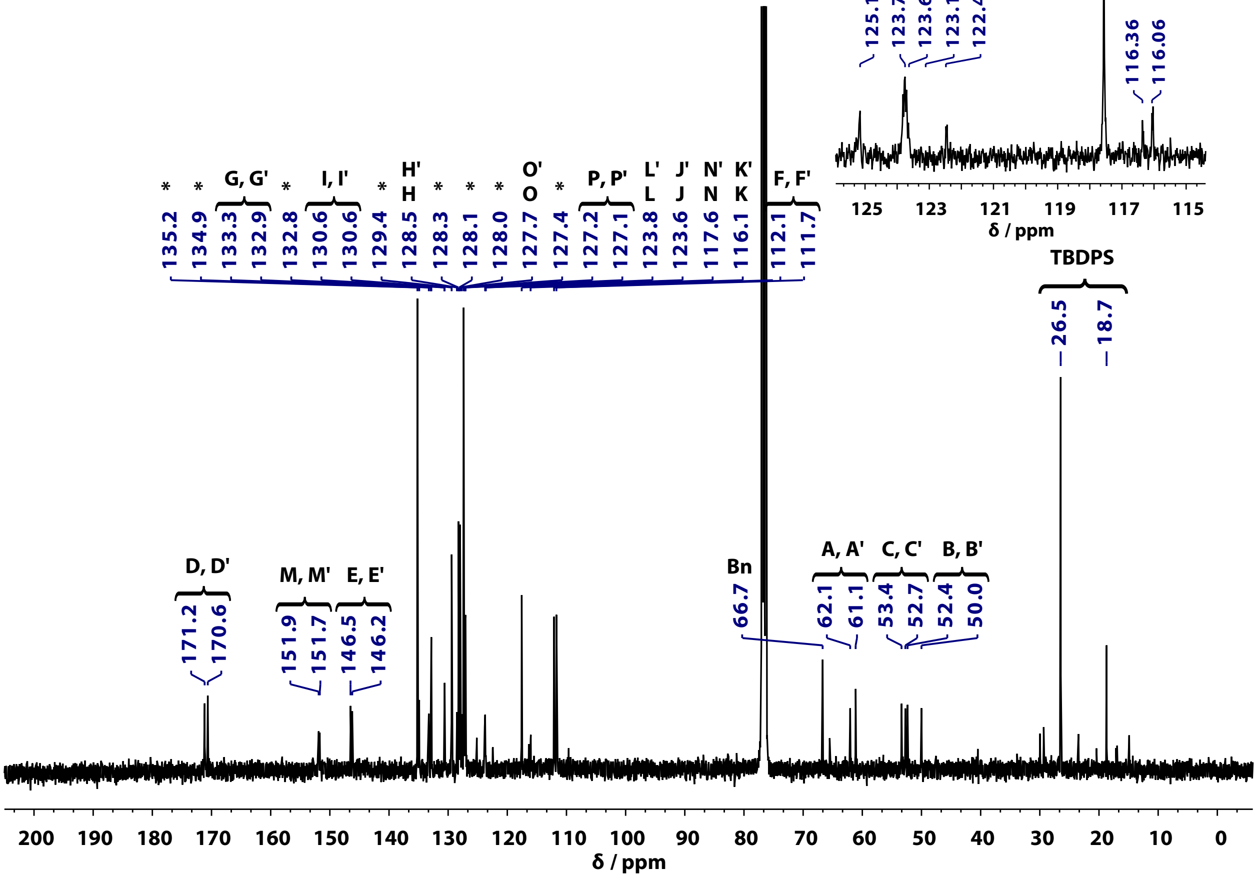
125.15 123.75 123.63 123.11 122.48
116.36 116.06
125 123 121 119 117 115
δ / ppm
* * G, G' * I, I' * H' H * * * O' O * P, P' L' L J' J N' N K' K F, F'
135.2 134.9 133.3 132.9 132.8 130.6 130.6 129.4 128.5 128.3 128.1 128.0 127.7 127.4 127.2 127.1 123.8 123.6 117.6 116.1 112.1 111.7
TBDPS
26.5 18.7
D, D'
171.2 170.6
M, M'
151.9 151.7
E, E'
146.5 146.2
Bn
66.7
A, A'
62.1 61.1
C, C'
53.4 52.7
B, B'
52.4 50.0
200 190 180 170 160 150 140 130 120 110 100 90 80 70 60 50 40 30 20 10 0
δ / ppm

2.15 Synthesis of 18

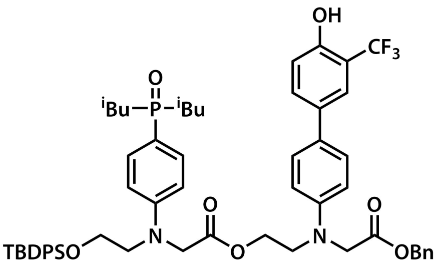

Compounds **13** (23 mg, 0.04 mmol) and **14** (19 mg, 0.04 mmol) in anhydrous dichloromethane (5 ml) with *N,N*-dimethylaminopyridine (1.5 mg, 0.01 mmol) and EDC·HCl (4 mg, 0.05 mmol) were stirred overnight under nitrogen atmosphere. The mixture was poured into water (10 ml) and the aqueous layer was extracted with dichloromethane (3 × 5 ml). The organic extracts were washed with brine (20 ml), dried with anhydrous magnesium sulfate, filtered and concentrated under vacuum. Purification using column chromatography (0-100% ethyl acetate in petroleum ether) gave **18**-acetate as a white wax (22 mg, 0.02 mmol).

The intermediate (22 mg, 0.02 mmol) and ammonium acetate (40 mg, 0.5 mmol) were dissolved in methanol (1 ml) and water (0.25 ml). The reaction was stirred under nitrogen atmosphere overnight. After completion, the mixture was partitioned between water (10 ml) and ethyl acetate (10 ml). The layers were separated and the aqueous layer was subsequently washed with dichloromethane (2 × 10 ml). The organic extracts were washed with brine (10 ml), dried with anhydrous magnesium sulfate, filtered and concentrated under vacuum to yield compound **18** as a colourless oil (20 mg, 0.04 mmol, 52% over two steps).

^{1}H NMR (400 MHz, $CDCl_3$) δ 10.24 (br s, 1H), 7.67 – 7.59 (m, 5H), 7.50 – 7.31 (m, 16H), 7.21 (d, J = 8.5 Hz, 2H), 6.70 (d, J = 8.9 Hz, 2H), 6.49 (d, J = 8.6 Hz, 2H), 5.20 (s, 2H), 4.40 (t, J = 5.9 Hz, 2H), 4.08 (s, 2H), 4.03 (s, 2H), 3.84 (t, J = 5.9 Hz, 2H), 3.71 (t, J = 6.0 Hz, 2H), 3.55 (t, J = 5.9 Hz, 2H), 2.04 – 1.80 (m, 4H), 1.73 – 1.65 (m, 2H), 1.05 (s, 9H), 1.03 (d, J = 6.9 Hz, 6H), 0.86 (d, J = 6.7 Hz, 6H) ppm.

^{13}C NMR (101 MHz, $CDCl_3$) δ 170.7, 170.4, 155.0, 150.1 (d, J = 2= Hz), 146.5, 135.5, 135.4, 133.1, 131.8 (d, J = 10= Hz), 131.3, 130.6, 129.8, 128.6, 128.5, 128.4, 127.8, 127.5, 124.3 (d, J = 273 Hz), 124.3 (q, J = 5= Hz), 118.5 (d, J = 100 Hz), 117.9, 116.9 (q, J = 30= Hz), 112.6, 111.5 (d, J = 12= Hz), 77.2, 66.9, 62.4, 61.3, 53.3, 52.6 (d, J = 15 Hz), 50.3, 39.8 (d, J = 68= Hz), 26.8, 24.7 (d, J = 9 Hz), 24.5, 23.4 (d, J = 4 Hz), 19.0 ppm.

^{19}F NMR (376 MHz, $CDCl_3$) δ -61.6 ppm.

^{31}P NMR(162 MHz, $CDCl_3$) δ 41.2 ppm.

FT-IR (neat): 3072 (br), 3037, 2957, 2870, 2741, 1746 (br), 1598, 1504 cm^{-1}.

HR-MS (ESI): m/z = 1021.4533 $[M+H]^+$ (calcd. for $C_{58}H_{69}F_3N_2O_7PSi$: 1021.4558, Δ 3.0 ppm).

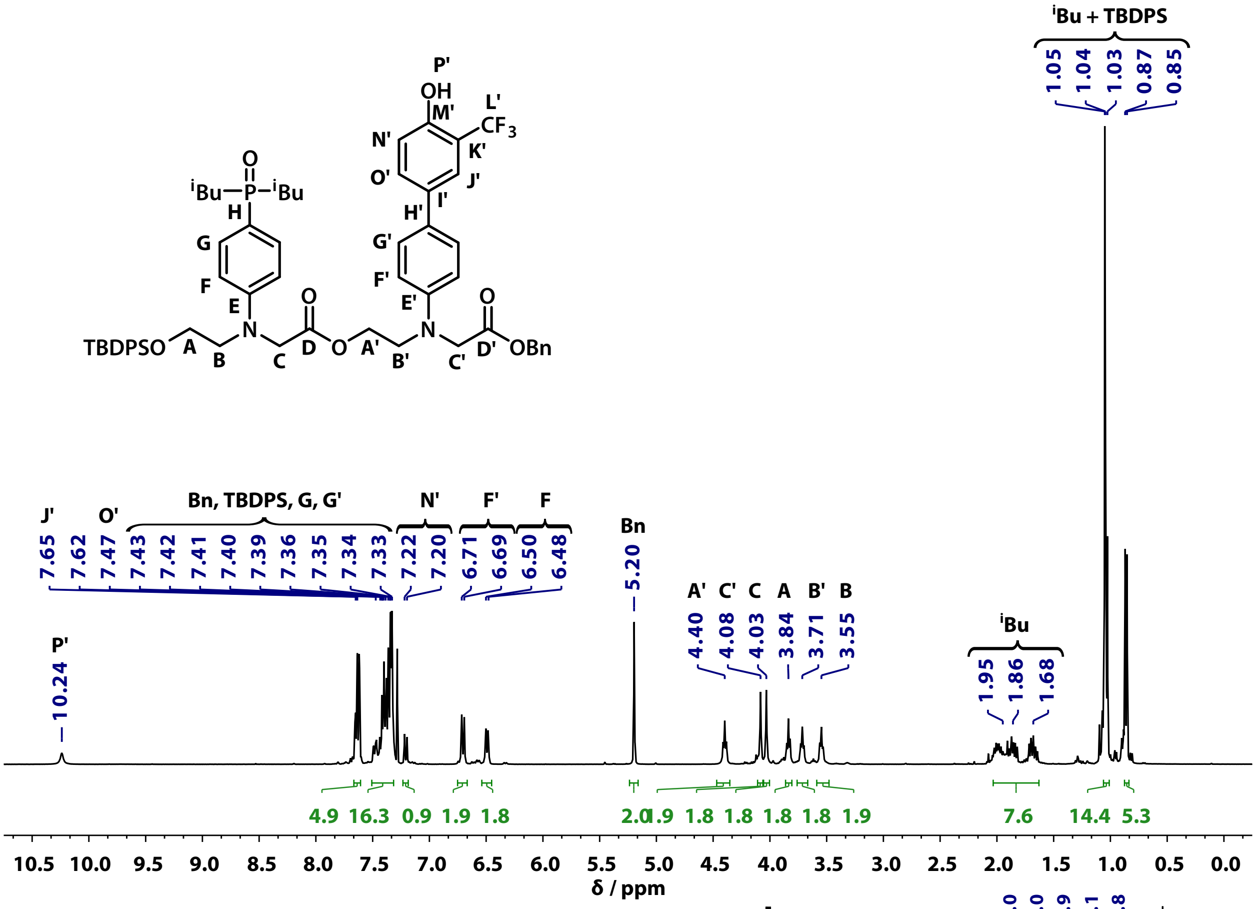

P'
OH
M'
L'
CF3
N'
K'
O'
J'
O
iBu—P—iBu
H
I'
H'
G
G'
F
F'
E
E'
TBDPSO
A
B
N
C
O
D
O
A'
B'
N
C'
O
D'
OBn
iBu + TBDPS
1.05
1.04
1.03
0.87
0.85
J'
O'
Bn, TBDPS, G, G'
N'
F'
F
7.65
7.62
7.47
7.43
7.42
7.41
7.40
7.39
7.36
7.35
7.34
7.33
7.22
7.20
6.71
6.69
6.50
6.48
Bn
5.20
A' C' C A B' B
4.40
4.08
4.03
3.84
3.71
3.55
P'
10.24
iBu
1.95
1.86
1.68
4.9 16.3 0.9 1.9 1.8
2.0 1.9 1.8 1.8 1.8 1.8 1.9
7.6
14.4 5.3
10.5 10.0 9.5 9.0 8.5 8.0 7.5 7.0 6.5 6.0 5.5 5.0 4.5 4.0 3.5 3.0 2.5 2.0 1.5 1.0 0.5 0.0
δ / ppm

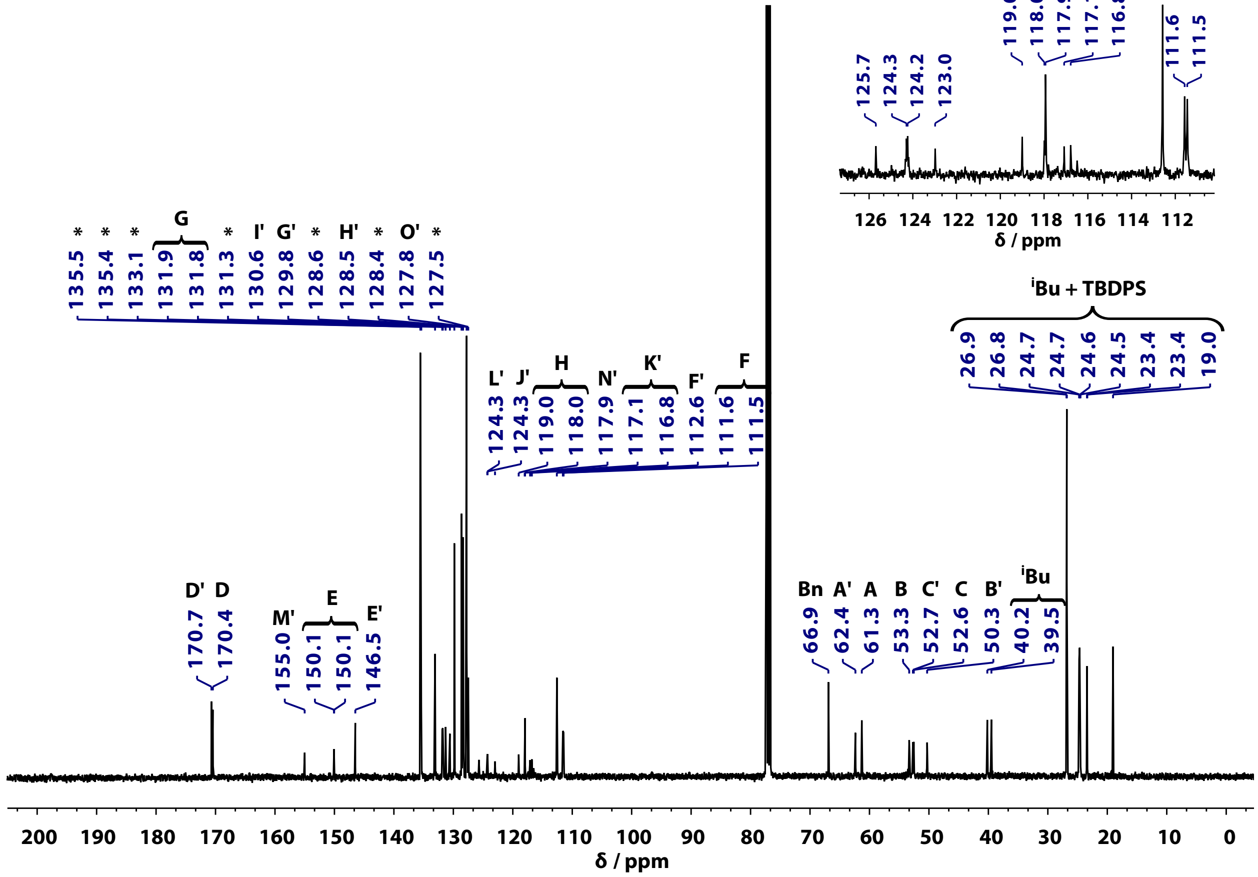

125.7
124.3
124.2
123.0
119.0
118.0
117.9
117.1
116.8
111.6
111.5
126 124 122 120 118 116 114 112
δ / ppm
* * * G * I' G' * H' * O' *
135.5
135.4
133.1
131.9
131.8
131.3
130.6
129.8
128.6
128.5
128.4
127.8
127.5
iBu + TBDPS
26.9
26.8
24.7
24.7
24.6
24.5
23.4
23.4
19.0
L' J' H N' K' F' F
124.3
124.3
119.0
118.0
117.9
117.1
116.8
112.6
111.6
111.5
D' D
170.7
170.4
M' E E'
155.0
150.1
150.1
146.5
Bn A' A B C' C B' iBu
66.9
62.4
61.3
53.3
52.7
52.6
50.3
40.2
39.5
200 190 180 170 160 150 140 130 120 110 100 90 80 70 60 50 40 30 20 10 0
δ / ppm

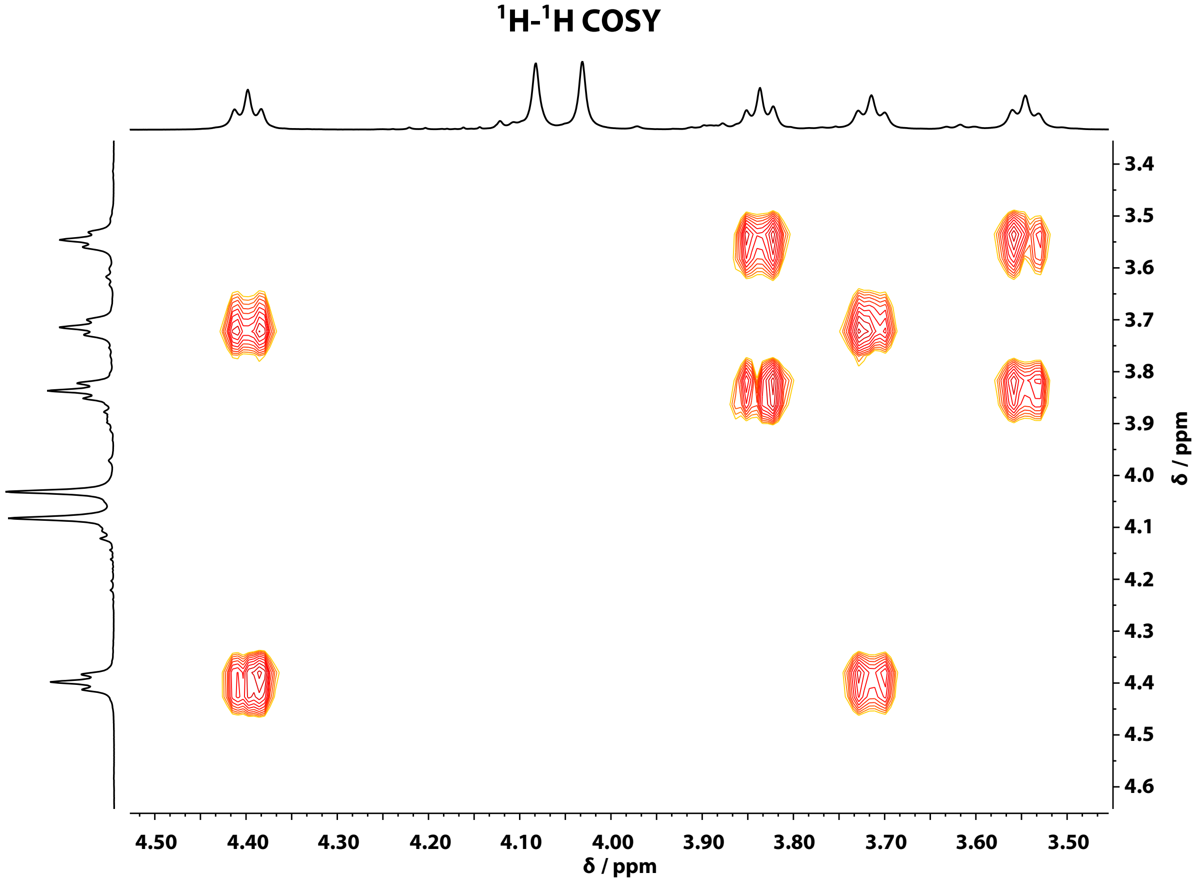

^{1}H-^{1}H COSY
δ / ppm
δ / ppm

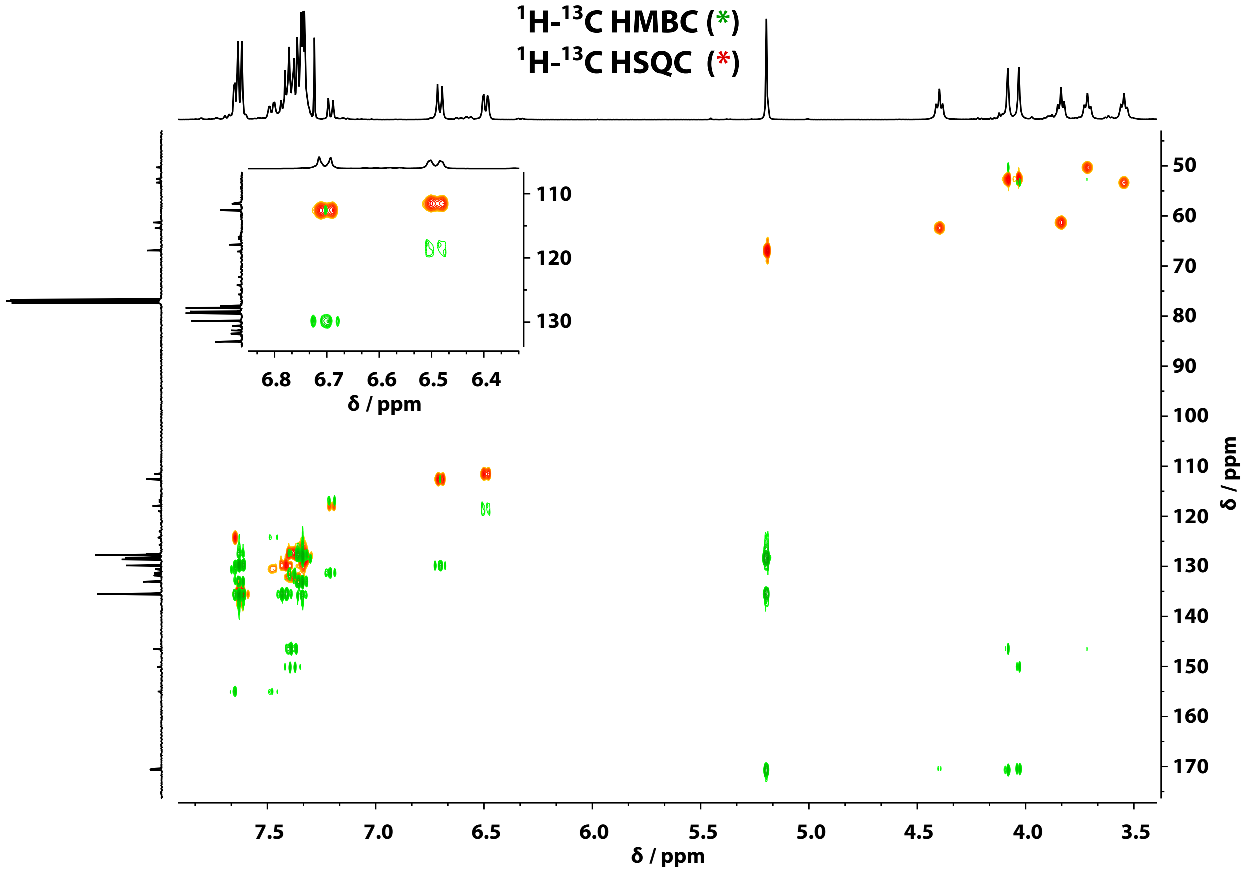

^{1}H-^{13}C HMBC (*)
^{1}H-^{13}C HSQC (*)
δ / ppm
δ / ppm
δ / ppm

3 1:1 Binding Isotherm Derivation and Implementation

Equilibrium constant $K_{\mathbf{H \cdot G}}$ for an interaction between host **H** and guest **G** is defined as:

$$\mathbf{H} + \mathbf{G} \xrightleftharpoons{K_{\mathbf{H \cdot G}}} \mathbf{H \cdot G} \tag{1}$$

$$K_{\mathbf{H \cdot G}} = \frac{[\mathbf{H \cdot G}]}{[\mathbf{H}][\mathbf{G}]} \tag{2}$$

where [**H**], [**G**], and [**H·G**] are the equilibrium concentrations of the free host, the free guest and the resulting host-guest complex, respectively. Assuming that no other equilibria occur in the mixture, the total concentrations of the host $[\mathbf{H}]_0$ and of the guest $[\mathbf{G}]_0$ are simply:

$$[\mathbf{H}]_0 = [\mathbf{H}] + [\mathbf{H \cdot G}] \tag{3}$$

$$[\mathbf{G}]_0 = [\mathbf{G}] + [\mathbf{H \cdot G}] \tag{4}$$

Hence, the equilibrium constant $K_{\mathbf{H \cdot G}}$ can be alternatively expressed as:

$$K_{\mathbf{H \cdot G}} = \frac{[\mathbf{H \cdot G}]}{\left([\mathbf{H}]_0 - [\mathbf{H \cdot G}]\right)\left([\mathbf{G}]_0 - [\mathbf{H \cdot G}]\right)} \tag{5}$$

which can be easily rearranged to give a quadratic in [**H·G**]:

$$[\mathbf{H \cdot G}]^2 - [\mathbf{H \cdot G}]\left([\mathbf{H}]_0 + [\mathbf{G}]_0 + \frac{1}{K_{\mathbf{H \cdot G}}}\right) + [\mathbf{H}]_0[\mathbf{G}]_0 = 0 \tag{6}$$

Equation 6 has only one physically meaningful root:

$$[\mathbf{H \cdot G}] = \frac{1}{2}\left([\mathbf{H}]_0 + [\mathbf{G}]_0 + \frac{1}{K_{\mathbf{H \cdot G}}}\right) - \frac{1}{2}\sqrt{\left([\mathbf{H}]_0 + [\mathbf{G}]_0 + \frac{1}{K_{\mathbf{H \cdot G}}}\right)^2 - 4[\mathbf{H}]_0[\mathbf{G}]_0} \tag{7}$$

which gives the molar fraction of bound host $\chi_{\mathbf{H \cdot G}}$ as:

$$\chi_{\mathbf{H \cdot G}} = \frac{[\mathbf{H \cdot G}]}{[\mathbf{H}]_0} = \frac{1}{2[\mathbf{H}]_0}\left(1 + \frac{[\mathbf{G}]_0}{[\mathbf{H}]_0} + \frac{1}{K_{\mathbf{H \cdot G}}[\mathbf{H}]_0}\right) - \frac{1}{2[\mathbf{H}]_0}\sqrt{\left([\mathbf{H}]_0 + [\mathbf{G}]_0 + \frac{1}{K_{\mathbf{H \cdot G}}}\right)^2 - 4[\mathbf{H}]_0[\mathbf{G}]_0} \tag{8}$$

In the fast-exchange regime of NMR spectroscopy, the observed chemical shift δ_{obs} is a weighted average of the signals arising from all species present in solution:

$$\delta_{obs} = \delta_H \frac{[H]}{[H]_0} + \delta_{H \cdot G} \frac{[H \cdot G]}{[H]_0} = \delta_H (1 - \chi_{H \cdot G}) + \delta_{H \cdot G} \chi_{H \cdot G} \tag{9}$$

where δ_H and $\delta_{H \cdot G}$ are the chemical shifts corresponding to the free **H** and the fully bound **H·G** complex, respectively. A model defined by Equations (8) and (9) can be fitted to the NMR data with $K_{H \cdot G}$, $\delta_{H \cdot G}$, and δ_H as parameters, using **lmfit** package in Python.[3]

```
# FITTING 1:1 BINDING ISOTHERM TO NMR TITRATION DATA
# Non-linear regression done using lmfit
# https://lmfit.github.io/lmfit-py/model.html

from lmfit import Parameters, Model

# Define model parameters with initial values
# add with tuples: (NAME VALUE VARY MIN  MAX  EXPR  BRUTE_STEP)

params = Parameters()
params.add_many(('K', 1000, True, None, None, None, None),
                ('d_bound', -70, True, None, None, None),
                ('d_free', -50, True, None, None, None, None))

# Molar fraction of bound host from two-state equilibrium constant

def alpha(c_guest, K):
    return 0.5*(1 + c_guest/c_host + 1/(K*c_host)) - ((0.5/c_host) * \
    np.sqrt((c_host + c_guest + 1/(K))*(c_host + c_guest + 1/(K)) - 4*c_guest*c_host))

# MODEL FUNCTION: Observed chemical shift (weighted average)

def d_obs(c_guest, K, d_bound, d_free):
    return d_bound * alpha(c_guest, K) + d_free * (1 - alpha(c_guest, K))

# Fitting parameters to the data
# xvalues : dataframe containing guest concentrations (M)
# yvalues : dataframe containing observed chemical shifts (ppm)
# c_host : float containing host concentration (M)
# List of minimisation methods: https://lmfit.github.io/lmfit-py/fitting.html
# Default minimisation method='leastsq' is Levenberg-Marquardt algorithm
# Here by default use robust Nelder-Mead method
#       and then estimate confidence interval from L-M covariance matrix
# results : contains best fit parameters

model = Model(d_obs)
results = model.fit(yvalues, params, c_guest=xvalues, method='nelder')
results2 = model.fit(yvalues, params=results.params, c_guest=xvalues, method='leastsq')

# Print best fit parameters and fit statistics

print(results.fit_report() + '\n' + results2.fit_report())
```

4 Dimerisation Isotherm Derivation and Implementation

Equilibrium constant $K_{\mathbf{M\cdot M}}$ the dimerisation of a self-complementary monomer **M** to form **M·M** is:

$$2\,\mathbf{M} \xrightleftharpoons{K_{\mathbf{M\cdot M}}} \mathbf{M\cdot M} \tag{10}$$

$$K_{\mathbf{M\cdot M}} = \frac{[\mathbf{M\cdot M}]}{[\mathbf{M}]^2} \tag{11}$$

where [**M**] and [**M·M**], are the equilibrium concentrations of the free monomer and the dimer. Assuming that no other equilibria occur in the mixture, the total concentration of the dimerising molecule $[\mathbf{M}]_0$is simply:

$$[\mathbf{M}]_0 = [\mathbf{M}] + 2[\mathbf{M\cdot M}] \tag{12}$$

Hence, the equilibrium constant $K_{\mathbf{M\cdot M}}$ can alternatively expressed as:

$$K_{\mathbf{M\cdot M}} = \frac{[\mathbf{M\cdot M}]}{\left([\mathbf{H}]_0 - 2[\mathbf{M\cdot M}]\right)^2} \tag{13}$$

which can be easily rearranged to give a quadratic in [**M·M**]:

$$[\mathbf{M\cdot M}]^2 - [\mathbf{M\cdot M}]\left([\mathbf{M}]_0 + \frac{1}{4K_{\mathbf{M\cdot M}}}\right) + \frac{1}{4}[\mathbf{M}]_0^2 = 0 \tag{14}$$

Equation 14 has only one physically meaningful root:

$$[\mathbf{M\cdot M}] = \frac{1}{2}\left([\mathbf{M}]_0 + \frac{1}{4K_{\mathbf{M\cdot M}}}\right) - \frac{1}{2}\sqrt{\left([\mathbf{M}]_0 + \frac{1}{4K_{\mathbf{M\cdot M}}}\right)^2 - [\mathbf{M}]_0^2} \tag{15}$$

which gives the molar fraction of the dimer $\chi_{\mathbf{M\cdot M}}$ as:

$$\chi_{\mathbf{M\cdot M}} = \frac{2[\mathbf{M\cdot M}]}{[\mathbf{M}]_0} = 1 + \frac{1}{4[\mathbf{M}]_0 K_{\mathbf{M\cdot M}}} - \sqrt{\left(1 + \frac{1}{4[\mathbf{M}]_0 K_{\mathbf{M\cdot M}}}\right)^2 - 1} \tag{16}$$

In the fast-exchange regime of NMR spectroscopy, the observed chemical shift δ_{obs} is a weighted average of the signals arising from all species present in solution:

$$\delta_{\text{obs}} = \delta_{\mathbf{M}}\frac{[\mathbf{M}]}{[\mathbf{M}]_0} + \delta_{\mathbf{M\cdot M}}\frac{2[\mathbf{M\cdot M}]}{[\mathbf{M}]_0} = \delta_{\mathbf{M}}(1 - \chi_{\mathbf{M\cdot M}}) + \delta_{\mathbf{M\cdot M}}\chi_{\mathbf{M\cdot M}} \tag{17}$$

where δ_{M} and $\delta_{M \cdot M}$ are the chemical shifts corresponding to the free **M** and the fully bound **M·M** complex, respectively. A model defined by Equations (16) and (17) can be fitted to the NMR data with $K_{M \cdot M}$, $\delta_{M \cdot M}$, and δ_{M} as parameters, using **lmfit** package in Python.[3]

Values in the text are quoted as arithmetic means and the errors were estimated as 95% confidence intervals based on at least two repetitions.

```
# FITTING DIMERISATION ISOTHERM TO NMR DILUTION DATA
# Non-linear regression done using lmfit
# https://lmfit.github.io/lmfit-py/model.html

from lmfit import Parameters, Model

# Define model parameters with initial values
# add with tuples: (NAME VALUE VARY MIN  MAX  EXPR  BRUTE_STEP)

params = Parameters()
params.add_many ((’K’, 100, True, None, None, None, None),
                ('d_bound’, -70, True, None, None, None),
                (’d_free’, -50, True, None, None, None, None))

# Molar fraction from the two-state dimerisation equilibrium

def alpha(c, K):
    return (1 + 1/(4*K*c) - np.sqrt((1 + 1/(4*K*c))*(1 + 1/(4*K*c)) - 1))

# MODEL FUNCTION: Observed chemical shift (weighted average)

def d_obs(c, K, d_bound, d_free):
    return d_bound * alpha(c, K) + d_free * (1 - alpha(c,K))

# Fitting parameters to the data
# xvalues : dataframe containing total concentration (M)
# yvalues : dataframe containing observed chemical shifts (ppm)
# List of minimisation methods: https://lmfit.github.io/lmfit-py/fitting.html
# Default minimisation method=’leastsq’ is Levenberg-Marquardt algorithm
# Here by default use robust Nelder-Mead method
#      and then estimate confidence interval from L-M covariance matrix
# results : contains best fit parameters

model = Model(d_obs)
results = model.fit(yvalues, params, c=xvalues, method=’nelder’)
results2 = model.fit(yvalues, params=results.params, c=xvalues, method=’leastsq’)

# Print best fit parameters and fit statistics

print(results.fit_report() + ’\n’ + results2.fit_report())
```

5 Implementation of 1:2 Binding Isotherm

A 1:2 equilibrium mixture between host **H** and guest **G**:

$$\mathrm{H + G \underset{}{\overset{K_1}{\rightleftarrows}} H{\cdot}G} \tag{18}$$

$$\mathrm{H{\cdot}G + A \underset{}{\overset{K_2}{\rightleftarrows}} H{\cdot}2G} \tag{19}$$

can be analysed analogously to the previous systems and the equilibrium concentration of the guest [G] can then be expressed as a cubic equation:

$$K_1K_2[\mathrm{G}]^3 + K_1\left\{(2K_2[\mathrm{H}]_0 - K_2[\mathrm{G}]_0 + 1)\right\}[\mathrm{G}]^2 + \left\{K_1([\mathrm{H}]_0 - [\mathrm{G}]_0 + 1\right\}[\mathrm{G}] - [\mathrm{G}]_0 = 0 \tag{20}$$

where $[\mathrm{H}]_0$ and $[\mathrm{G}]_0$ are total concentrations of the host and guest in solution. In the fast-exchange regime of NMR spectroscopy, the observed chemical shift δ_{obs} is a weighted average of the signals arising from all species present in solution:

$$\delta_{\mathrm{obs}} = \delta_{\mathrm{H}}\frac{[\mathrm{H}]}{[\mathrm{H}]_0} + \delta_{\mathrm{H\cdot G}}\frac{[\mathrm{H\cdot G}]}{[\mathrm{H}]_0} + +\delta_{\mathrm{H\cdot 2G}}\frac{[\mathrm{H\cdot 2G}]}{[\mathrm{H}]_0} \tag{21}$$

where δ_{H}, $\delta_{\mathrm{H\cdot G}}$ and $\delta_{\mathrm{H\cdot 2G}}$ are the chemical shifts corresponding to the free **H**, **H·G** complex, and **H·2G** complex respectively. A model defined by Equations (20) and (21) can be fitted to the NMR data with K_1, K_2, $\delta_{\mathrm{H\cdot G2}}$, $\delta_{\mathrm{H\cdot G}}$, and δ_{H} as parameters, using **lmfit** package in Python.[3] Roots of the cubic equation for the equilibrium guest concentration are found numerically using the **numpy** package.[4] Analysis was performed in Jupyter and the results were plotted using **matplotlib**.[5,6]

```
# FITTING 1:2 BINDING ISOTHERM TO NMR TITRATION DATA
# Non-linear regression done using lmfit
# https://lmfit.github.io/lmfit-py/model.html

from lmfit import Parameters, Model
import numpy as np
import pandas as pd

# Define model parameters with initial values, fix K1*K2 = K_m * K_m

params = Parameters()
params.add_many(('K1', 7640, True, None, None, None, None),
                ('K2', 1910, True, None, None, '14622976/K1', None),
                ('d_HG2', -61.6, True, None, None, None),
                ('d_HG', -61.4, True, None, None, None),
                ('d_H', -61.3, True, None, None, None, None))
```

```
# MODEL FUNCTION: Observed chemical shift (weighted average)
# c_host : float containing total host concentration (M)

def d_obs(G, K1, K2, d_H, d_HG, d_HG2):
    H = (c_host)/(1+K1*G+K1*K2*G*G)
    HG = K1*H*G
    HG2 = HG*K2*G
    return H/c_host * d_H + HG/c_host * d_HG + HG2/c_host * d_HG2

# Objective function to be minimised
# Returns array of residuals of the model

def fit_function(params, c_guest, d_F):
  # Unpack the parameter values

    K1 = params['K1'].value
    K2 = params['K2'].value
    d_HG2 = params['d_HG2'].value
    d_HG = params['d_HG'].value
    d_H = params['d_H'].value

    # Solve cubic equation for equilibrium guest concentration
    # Solved numerically using numpy library
    # Meaningful solution is the smallest positive real root

    def Groot(G0):
        Gall = np.roots([K1*K2, K1*(2*K2*c_host-K2*G0+1), (K1*(c_host-G0)+1), -G0])
        real_valued = Gall.real[abs(Gall.imag)<1e-5]
        G = min(real_valued[real_valued >= 0])
        return G

    modelF = d_obs(c_guest.apply(Groot), K1, K2, d_H, d_HG, d_HG2)
    residF = d_F - modelF
    return residF

# Fitting parameters to the data
# c_guest : dataframe containing total guest concentrations (M)
# d_F : dataframe containing observed chemical shifts (ppm)
# results : contains best fit parameters

results = minimize(fit_function, params, args=(c_guest, d_F), method='nelder')
results2 = minimize(fit_function, params, args=(c_guest, d_F), method='leastsq')

print(fit_report(results) + '\n' + fit_report(results2))
```

6 NMR Studies

6.1 D · A Binding Isotherm - Repetition 1

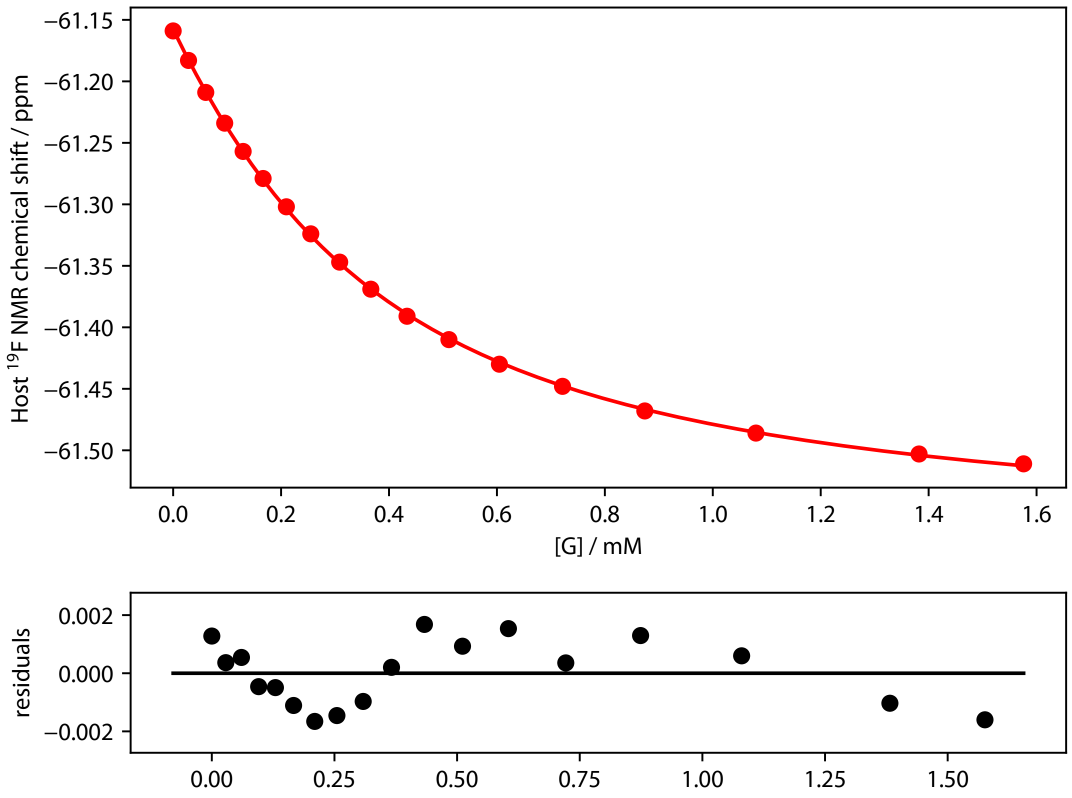

Fig. 1 Best fit 1:1 binding isotherm for **D** (host) and **A** (guest) in toluene-d_8 at 298 K against the original data.

```
[[Model]]
    Model(d_obs)
[[Fit Statistics]]
    # fitting method   = leastsq
    # function evals   = 5
    # data points      = 18
    # variables        = 3
    chi-square         = 2.1446e-05
    reduced chi-square = 1.4297e-06
    Akaike info crit   = -239.526414
    Bayesian info crit = -236.855299
[[Variables]]
    K:         3760.32707 +/- 55.3883020 (1.47%) (init = 3760.328)
    d_bound: -61.5796411 +/- 0.00163344 (0.00%) (init = -61.57964)
    d_free:  -61.1577205 +/- 7.3411e-04 (0.00%) (init = -61.15772)
[[Correlations]] (unreported correlations are < 0.100)
    C(K, d_bound)      =  0.917
    C(K, d_free)       =  0.632
    C(d_bound, d_free) =  0.404
```

6.2 D · A Binding Isotherm - Repetition 2

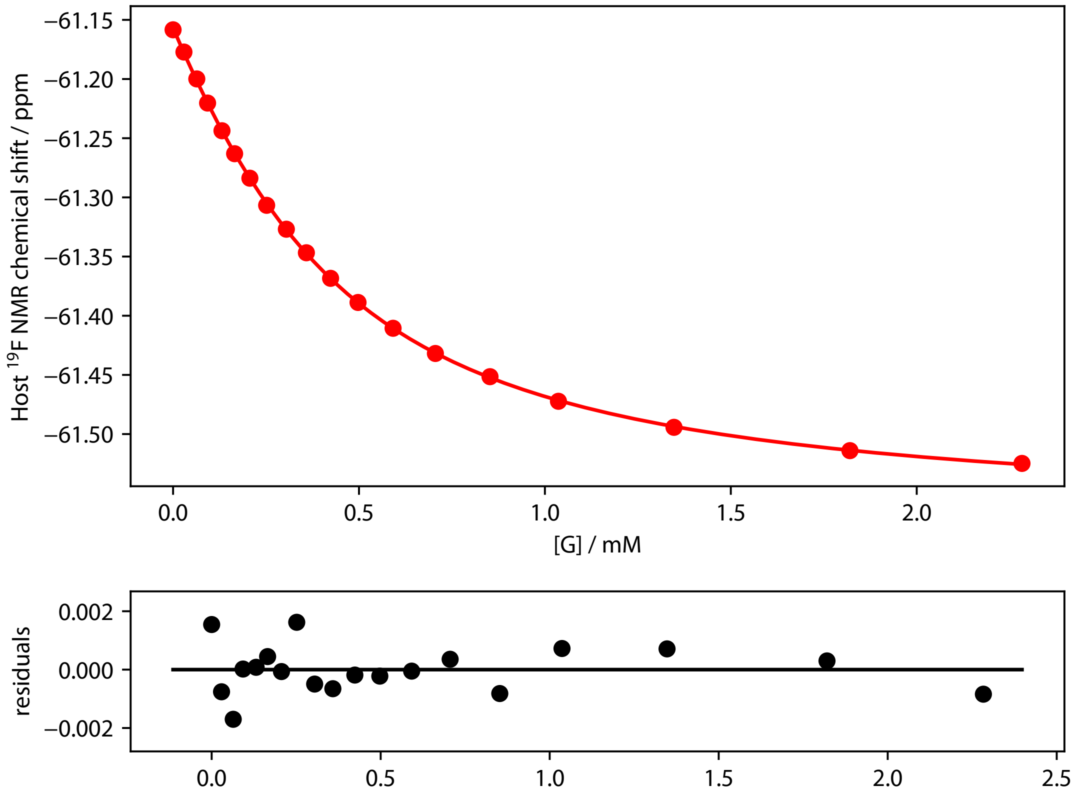

Fig. 2 Best fit 1:1 binding isotherm for **D** (host) and **A** (guest) in toluene-d_8 at 298 K against the original data.

```
[[Model]]
    Model(d_obs)
[[Fit Statistics]]
    # fitting method   = leastsq
    # function evals   = 5
    # data points      = 19
    # variables        = 3
    chi-square         = 1.2076e-05
    reduced chi-square = 7.5474e-07
    Akaike info crit   = -265.106108
    Bayesian info crit = -262.272791
[[Variables]]
    K:         3806.70174 +/- 39.3950098 (1.03%) (init = 3806.702)
    d_bound: -61.5736570 +/- 0.00102039 (0.00%) (init = -61.57366)
    d_free:  -61.1568555 +/- 4.8319e-04 (0.00%) (init = -61.15686)
[[Correlations]] (unreported correlations are < 0.100)
    C(K, d_bound)      =  0.900
    C(K, d_free)       =  0.599
    C(d_bound, d_free) =  0.353
```

6.3 D · A Binding Isotherm - Repetition 3

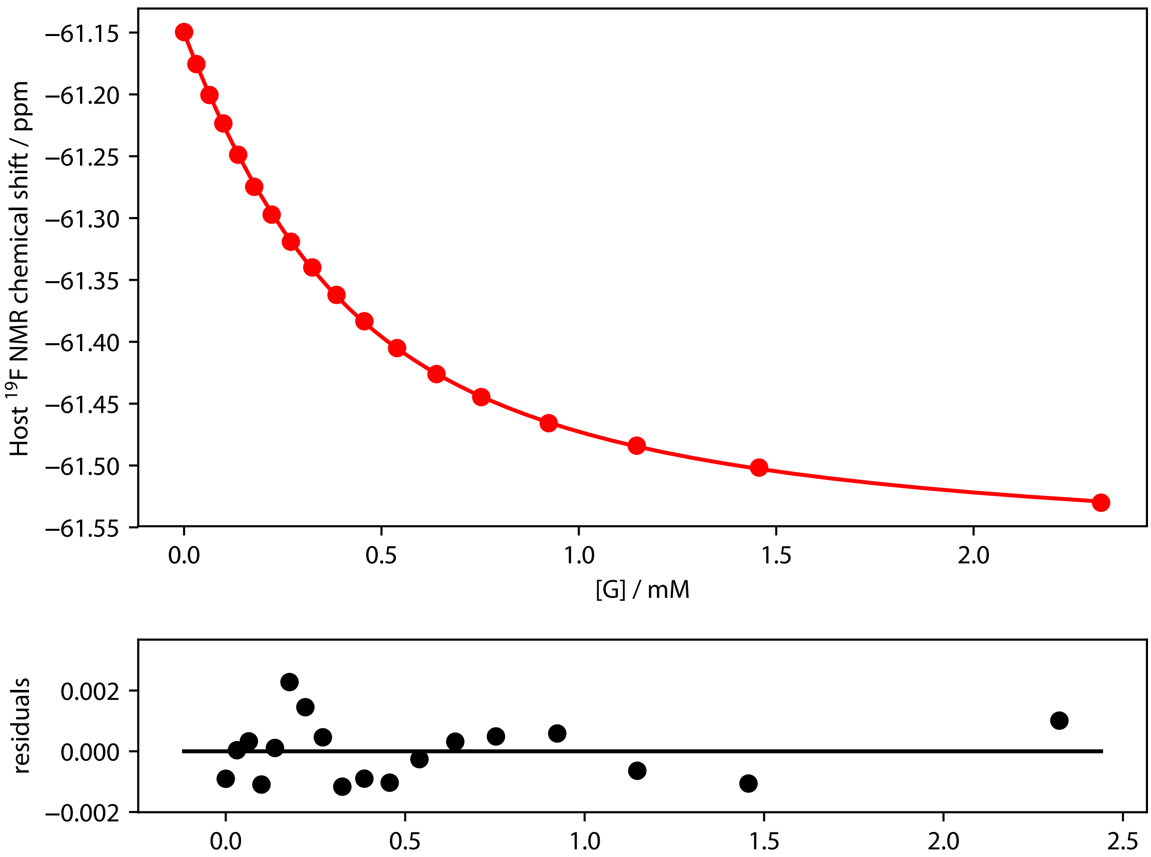

Fig. 3 Best fit 1:1 binding isotherm for **D** (host) and **A** (guest) in toluene-d_8 at 298 K against the original data.

```
[[Model]]
    Model(d_obs)
[[Fit Statistics]]
    # fitting method   = leastsq
    # function evals   = 5
    # data points      = 18
    # variables        = 3
    chi-square         = 1.6194e-05
    reduced chi-square = 1.0796e-06
    Akaike info crit   = -244.582326
    Bayesian info crit = -241.911211
[[Variables]]
    K:          3904.54897 +/- 47.7592566 (1.22%) (init = 3904.55)
    d_bound:  -61.5754544 +/- 0.00127523 (0.00%) (init = -61.57545)
    d_free:   -61.1505047 +/- 6.0897e-04 (0.00%) (init = -61.1505)
[[Correlations]] (unreported correlations are < 0.100)
    C(K, d_bound)      =  0.902
    C(K, d_free)       =  0.602
    C(d_bound, d_free) =  0.354
```

6.4 DD · AA Binding Isotherm - Repetition 1

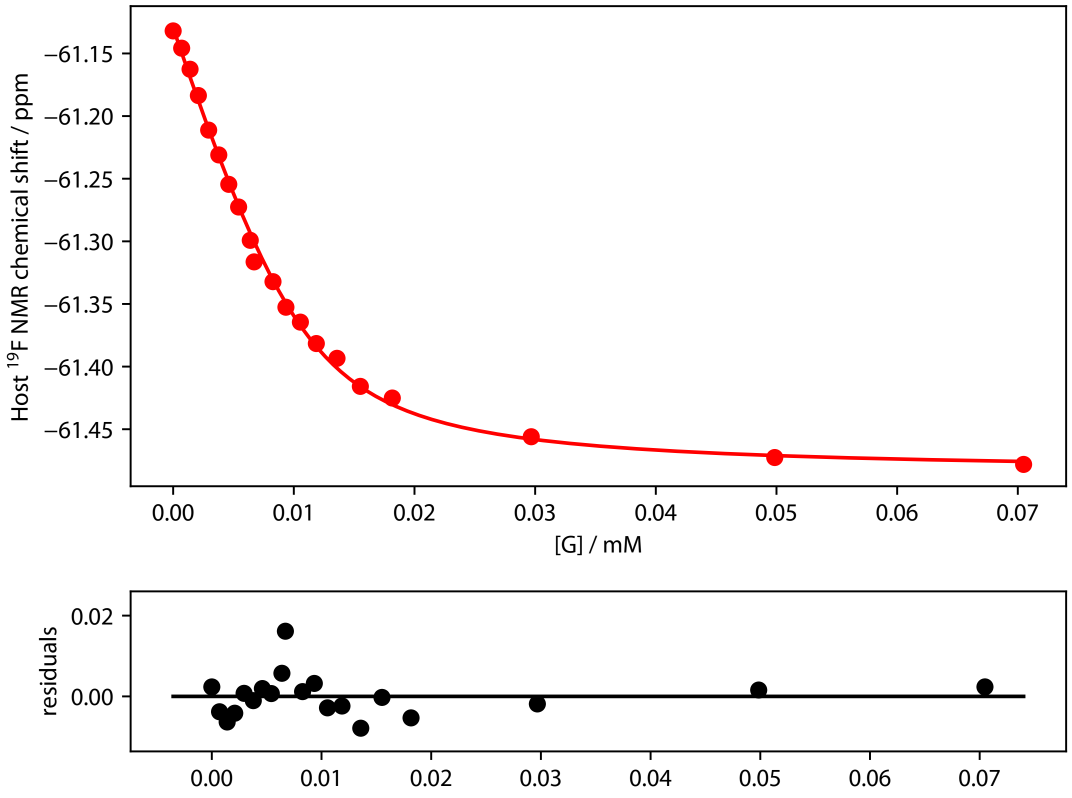

Fig. 4 Best fit 1:1 binding isotherm for **DD** (host) and **AA** (guest) in toluene-d_8 at 298 K against the original data.

```
[[Model]]
    Model(d_obs)
[[Fit Statistics]]
    # fitting method   = leastsq
    # function evals   = 5
    # data points      = 20
    # variables        = 3
    chi-square         = 5.0384e-04
    reduced chi-square = 2.9638e-05
    Akaike info crit   = -205.779699
    Bayesian info crit = -202.792502
[[Variables]]
    K:         609682.682 +/- 57865.2997 (9.49%) (init = 609685.6)
    d_bound: -61.4852187 +/- 0.00442042 (0.01%) (init = -61.48522)
    d_free:  -61.1296556 +/- 0.00257790 (0.00%) (init = -61.12966)
[[Correlations]] (unreported correlations are < 0.100)
    C(K, d_bound) =  0.857
    C(K, d_free)  =  0.359
```

6.5 DD · AA Binding Isotherm - Repetition 2

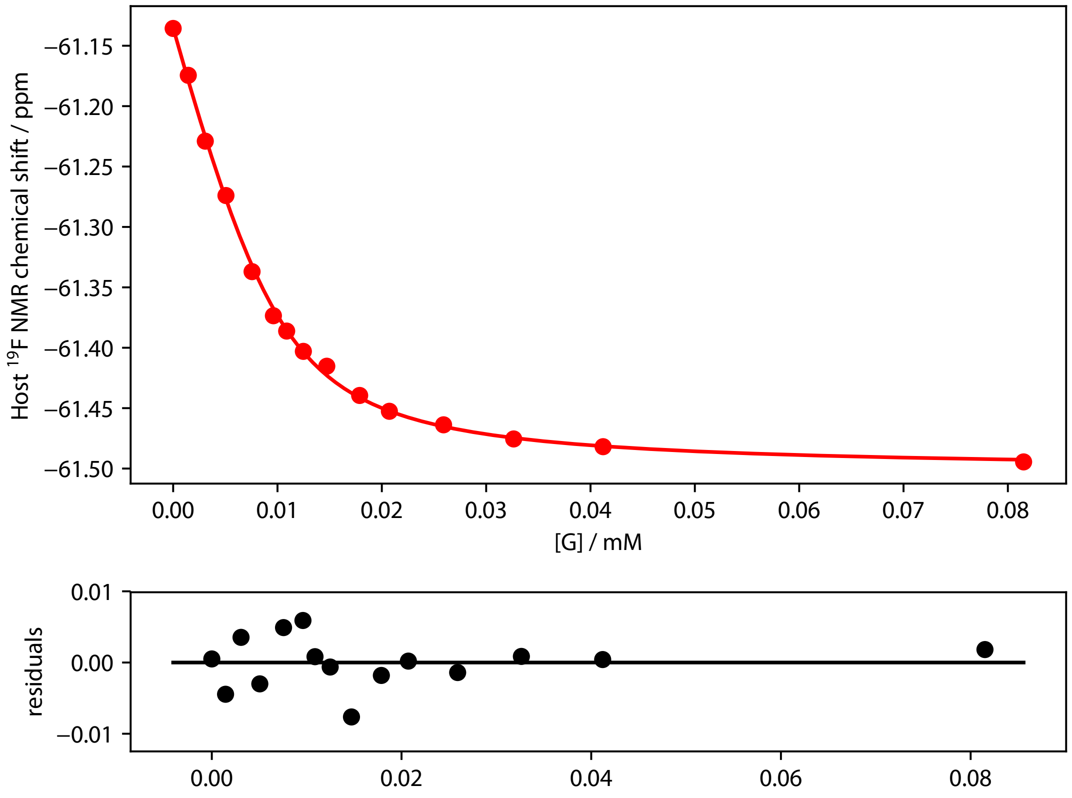

Fig. 5 Best fit 1:1 binding isotherm for **DD** (host) and **AA** (guest) in toluene-d_8 at 298 K against the original data.

```
[[Model]]
    Model(d_obs)
[[Fit Statistics]]
    # fitting method   = leastsq
    # function evals   = 5
    # data points      = 15
    # variables        = 3
    chi-square         = 1.6945e-04
    reduced chi-square = 1.4121e-05
    Akaike info crit   = -164.864998
    Bayesian info crit = -162.740847
[[Variables]]
    K:         528848.942 +/- 33609.4665 (6.36%) (init = 528849.1)
    d_bound: -61.5021016 +/- 0.00296419 (0.00%) (init = -61.5021)
    d_free:  -61.1351964 +/- 0.00246107 (0.00%) (init = -61.1352)
[[Correlations]] (unreported correlations are < 0.100)
    C(K, d_bound)      =  0.860
    C(K, d_free)       =  0.399
    C(d_bound, d_free) =  0.149
```

6.6 AD Dimerisation Isotherm - Repetition 1

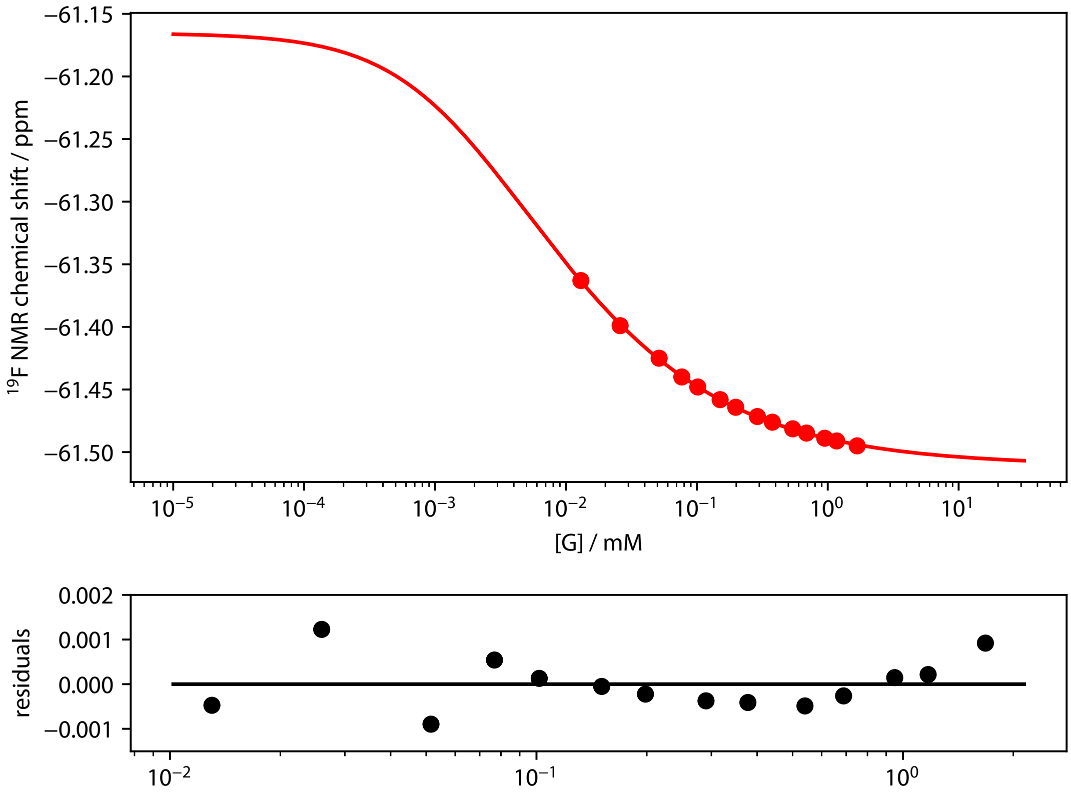

Fig. 6 Best fit dimerisation isotherm for **AD** in toluene-d_8 at 298 K against the original data.

```
[[Model]]
    Model(d_obs)
[[Fit Statistics]]
    # fitting method   = leastsq
    # function evals   = 5
    # data points      = 14
    # variables        = 3
    chi-square         = 4.4239e-06
    reduced chi-square = 4.0217e-07
    Akaike info crit   = -203.545571
    Bayesian info crit = -201.628399
[[Variables]]
    K:        121098.546 +/- 13490.0177 (11.14%) (init = 121098.3)
    d_bound: -61.5107889 +/- 5.2298e-04 (0.00%) (init = -61.51079)
    d_free:  -61.1654292 +/- 0.01440514 (0.02%) (init = -61.16543)
[[Correlations]] (unreported correlations are < 0.100)
    C(K, d_free)       =  0.996
    C(K, d_bound)      =  0.816
    C(d_bound, d_free) =  0.772
```

6.7 AD Dimerisation Isotherm - Repetition 2

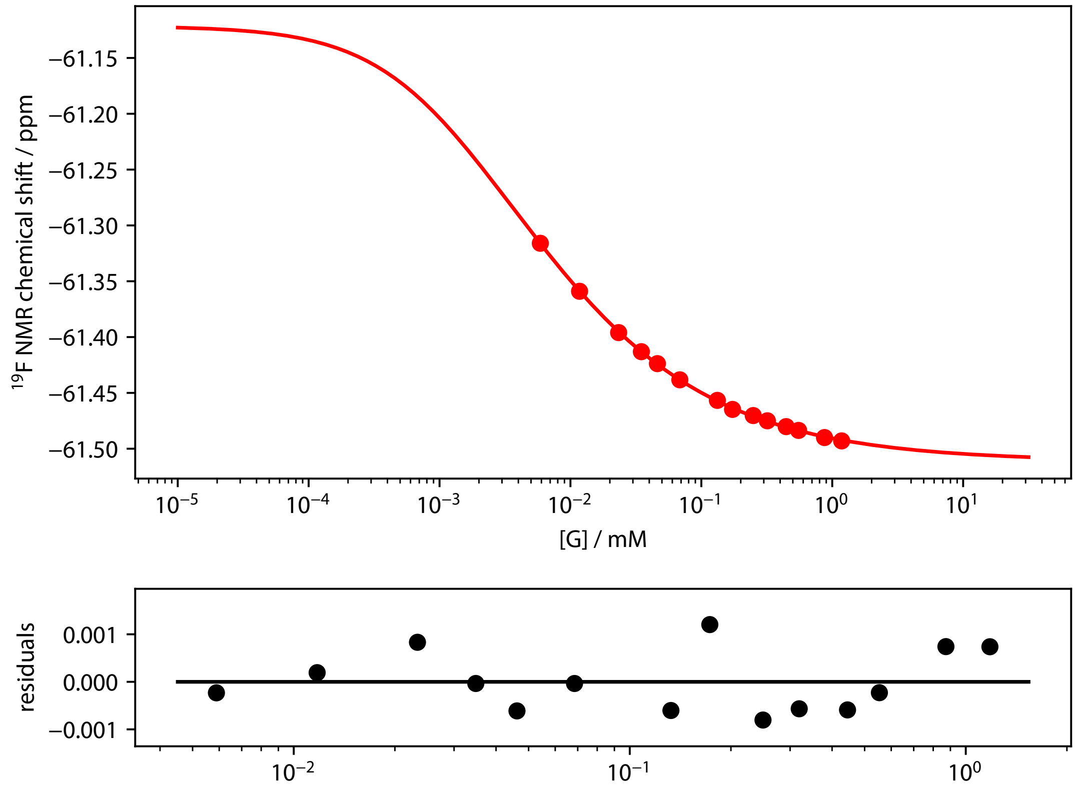

Fig. 7 Best fit dimerisation isotherm for **AD** in toluene-d_8 at 298 K against the original data.

```
[[Model]]
    Model(d_obs)
[[Fit Statistics]]
    # fitting method   = leastsq
    # function evals   = 5
    # data points      = 14
    # variables        = 3
    chi-square         = 5.4366e-06
    reduced chi-square = 4.9424e-07
    Akaike info crit   = -200.659739
    Bayesian info crit = -198.742567
[[Variables]]
    K:          169444.001 +/- 12080.0193 (7.13%) (init = 169446.3)
    d_bound: -61.5112713 +/- 5.1896e-04 (0.00%) (init = -61.51127)
    d_free:  -61.1215293 +/- 0.00978808 (0.02%) (init = -61.12153)
[[Correlations]] (unreported correlations are < 0.100)
    C(K, d_free)       =  0.993
    C(K, d_bound)      =  0.784
    C(d_bound, d_free) =  0.725
```

7 Double Hydrogen Bonding

Four different models were used to explain the change in the ^{19}F NMR signals upon addition of **A** into **DD**. The 1:1 binding model was not expected to give meaningful prediction as it would be chemically incorrect to assume only one binding interaction between divalent **DD** and monovalent **A** when **A** was in 20-fold excess.

Three different 1:2 binding models were investigated. Initially, the two binding sites in **DD** were assumed to be identical to the corresponding monomer binding site. Hence $K_1 * K_2 = K^2_{\mathbf{A\cdot D}}$, with $K_1 = 2K_{\mathbf{A\cdot D}}$ and $K_2 = 0.5K_{\mathbf{A\cdot D}}$ (including statistical factors). Such constraints could be implemented by not allowing the corresponding parameters to be varied during the fit:

```
params.add_many(('K1', 7640, False, None, None, None, None),
                ('K2', 1910, True, None, None, '0.25*K1', None),
                ...
```

Alternatively, the binding constants could be varied independently but their relationship was constrained as $K_2 = 0.25K_1$ to account for the binding sites being independent (corrected for the statistical factors). Their product was also allowed to vary:

```
params.add_many(('K1', 7640, True, None, None, None, None),
                ('K2', 1910, True, None, None, '0.25*K1', None),
                ...
```

The values for the two binding constants and their errors thus obtained were physically unreasonable. All above methods gave large residuals with possible sinusoidal trends, implying a possiblity of more complex binding equilibria. Hence, a 1:2 binding model with two independent binding sites was tested, where the only constraint was that $K_1 * K_2 = K^2_{\mathbf{A\cdot D}}$:

```
params.add_many(('K1', 7640, True, None, None, None, None),
                ('K2', 1910, True, None, None, '14622976/K1', None),
                ...
```

Comparison of the reduced χ^2 values and the Bayesian Information Criterion (BIC, which penalises free parameters more strongly) of all the above models suggests that the two binding sites are indeed independent and that the last model best explains the behaviour of the system (see Table 1). Difference in the BIC values of more than 10 provides very strong evidence against the model with the higher BIC value.[7] The best fit curves against the original data are shown in Fig. 8 and Fig. 9, alongside the residuals for each model.

Table 1 Fit statistics for the models used to explain the **DD-A** titration data.

Model	Reduced χ^2	BIC
**Repetition 1**		
1:1 binding	3.3×10^{-5}	−180
2 identical sites (fixed values)	5.5×10^{-5}	−171
2 identical sites (variable)	5.9×10^{-5}	−168
2 independent sites	2.7×10^{-6}	−223
**Repetition 2**		
1:1 binding	5.7×10^{-6}	−224
2 identical sites (fixed values)	9.8×10^{-6}	−213
2 identical sites (variable)	8.5×10^{-6}	−214
2 independent sites	2.6×10^{-6}	−236

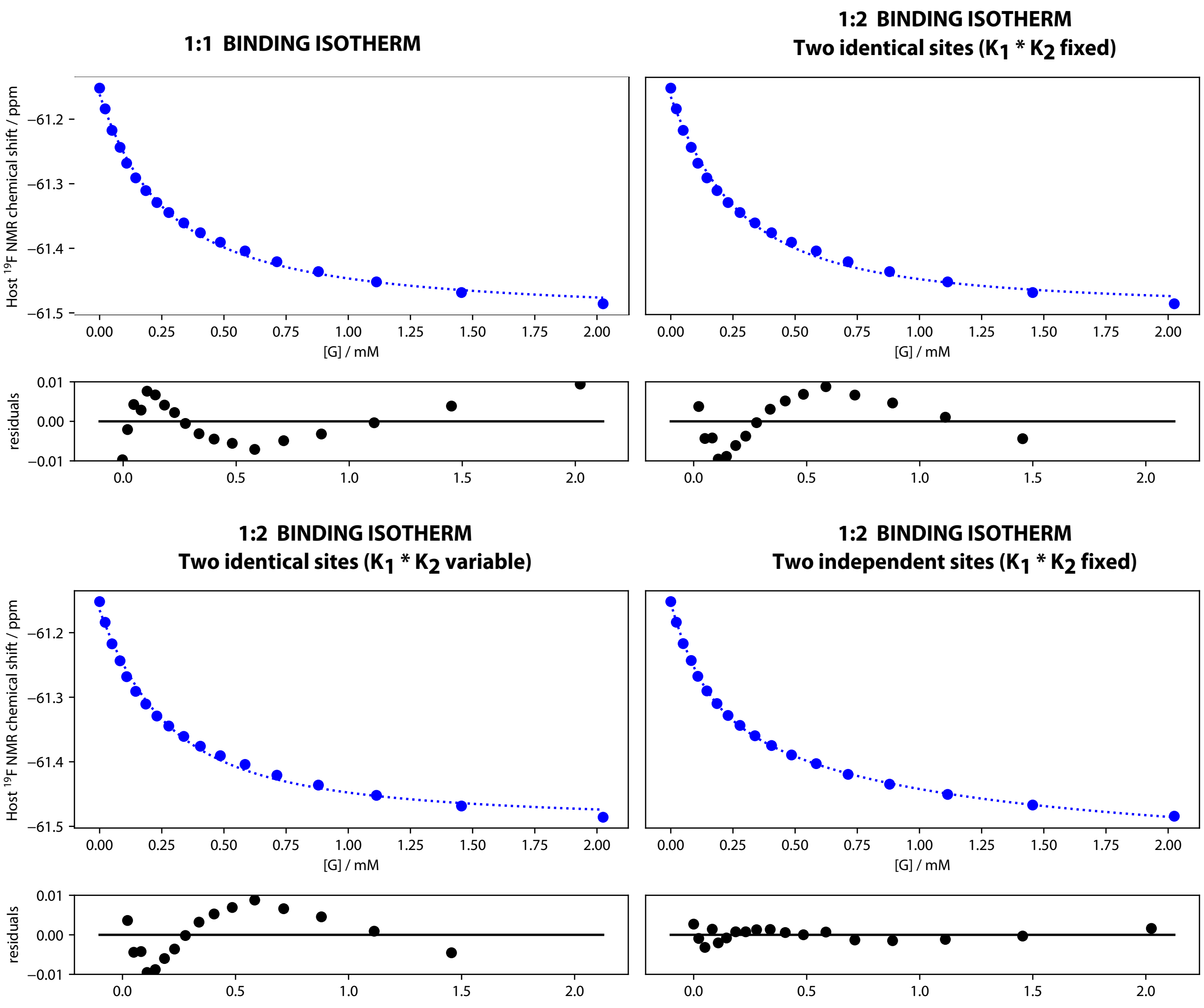

Fig. 8 Best fit isotherms for **DD** (host) and **A** (guest) in toluene-d_8 at 298 K against the original data.

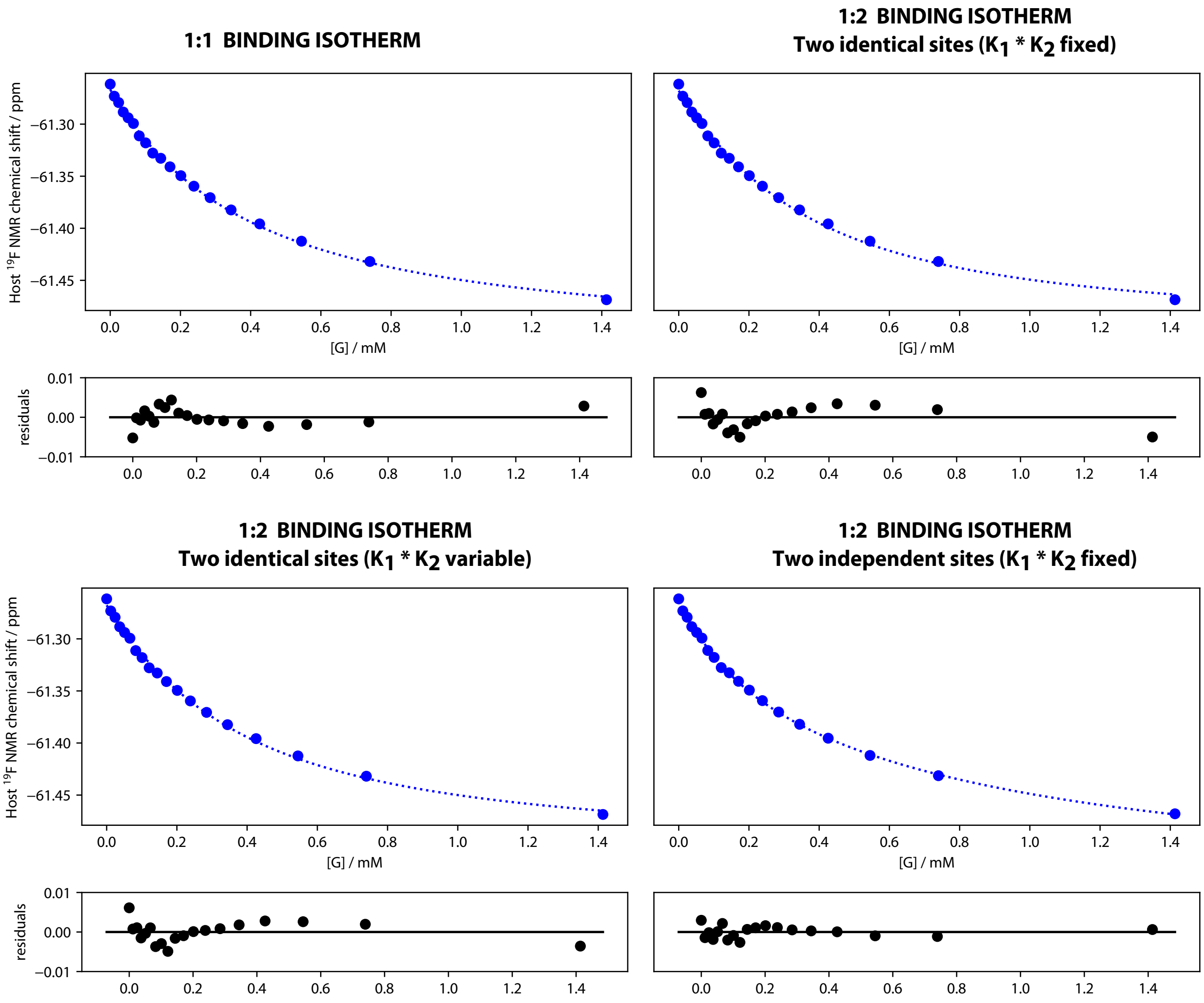

Fig. 9 Best fit isotherms for **DD** (host) and **A** (guest) in toluene-d_8 at 298 K against the original data.

7.1 DD · A 1:1 Binding Isotherm - Repetition 1

```
[[Model]]
    Model(d_obs)
[[Fit Statistics]]
    # fitting method   = leastsq
    # function evals   = 5
    # data points      = 18
    # variables        = 3
    chi-square         = 4.9913e-04
    reduced chi-square = 3.3275e-05
    Akaike info crit   = -182.874374
    Bayesian info crit = -180.203259
[[Variables]]
    K:        4957.50188 +/- 329.691907 (6.65%) (init = 4957.508)
    d_bound: -61.5093129 +/- 0.00537719 (0.01%) (init = -61.50931)
    d_free:  -61.1616175 +/- 0.00379355 (0.01%) (init = -61.16162)
[[Correlations]] (unreported correlations are < 0.100)
    C(K, d_bound)      =  0.860
    C(K, d_free)       =  0.653
    C(d_bound, d_free) =  0.356
```

7.2 DD · A 1:1 Binding Isotherm - Repetition 2

```
[[Model]]
    Model(d_obs)
[[Fit Statistics]]
    # fitting method   = leastsq
    # function evals   = 5
    # data points      = 19
    # variables        = 3
    chi-square         = 9.1921e-05
    reduced chi-square = 5.7451e-06
    Akaike info crit   = -226.541329
    Bayesian info crit = -223.708012
[[Variables]]
    K:        2997.41164 +/- 152.207001 (5.08%) (init = 2997.419)
    d_bound: -61.5155309 +/- 0.00456378 (0.01%) (init = -61.51553)
    d_free:  -61.2668274 +/- 0.00121275 (0.00%) (init = -61.26683)
[[Correlations]] (unreported correlations are < 0.100)
    C(K, d_bound)      =  0.919
    C(K, d_free)       =  0.647
    C(d_bound, d_free) =  0.418
```

7.3 DD · A 1:2 Binding Isotherm (Identical Sites, Fixed Values) - Repetition 1

```
[[Fit Statistics]]
    # fitting method   = leastsq
    # function evals   = 8
    # data points      = 18
    # variables        = 3
    chi-square         = 8.3167e-04
    reduced chi-square = 5.5445e-05
    Akaike info crit   = -173.684039
    Bayesian info crit = -171.012924
[[Variables]]
    K1:     7640 (fixed)
    d_HG2: -61.4990878 +/- 0.00767277 (0.01%) (init = -61.4991)
    d_HG:  -61.4083154 +/- 0.00894190 (0.01%) (init = -61.40832)
    d_H:   -61.1652504 +/- 0.00470929 (0.01%) (init = -61.16525)
    K2:     1910.00000 +/- 0.00000000 (0.00%) == '0.25*K1'
[[Correlations]] (unreported correlations are < 0.100)
    C(d_HG2, d_HG) = -0.775
    C(d_HG, d_H)   = -0.633
    C(d_HG2, d_H)  =  0.395
```

7.4 DD · A 1:2 Binding Isotherm (Identical Sites, Fixed Values) - Repetition 2

```
[[Fit Statistics]]
    # fitting method   = leastsq
    # function evals   = 8
    # data points      = 19
    # variables        = 3
    chi-square         = 1.5677e-04
    reduced chi-square = 9.7983e-06
    Akaike info crit   = -216.397820
    Bayesian info crit = -213.564503
[[Variables]]
    K1:     7640 (fixed)
    d_HG2: -61.5026521 +/- 0.00482125 (0.01%) (init = -61.50262)
    d_HG:  -61.3910907 +/- 0.00398488 (0.01%) (init = -61.39114)
    d_H:   -61.2678648 +/- 0.00162376 (0.00%) (init = -61.26785)
    K2:     1910.00000 +/- 0.00000000 (0.00%) == '0.25*K1'
[[Correlations]] (unreported correlations are < 0.100)
    C(d_HG2, d_HG) = -0.768
    C(d_HG, d_H)   = -0.670
    C(d_HG2, d_H)  =  0.412
```

7.5 DD · A 1:2 Binding Isotherm (Identical Sites) - Repetition 1

```
[[Fit Statistics]]
    # fitting method   = leastsq
    # function evals   = 7
    # data points      = 18
    # variables        = 4
    chi-square         = 8.2988e-04
    reduced chi-square = 5.9277e-05
    Akaike info crit   = -171.722884
    Bayesian info crit = -168.161397
[[Variables]]
    K1:     7992.64942 +/- 22756.4535 (284.72%) (init = 7996.674)
    d_HG2: -61.4991989 +/- 0.02932266 (0.05%) (init = -61.4992)
    d_HG:  -61.4023177 +/- 0.40770366 (0.66%) (init = -61.40225)
    d_H:   -61.1650961 +/- 0.00548269 (0.01%) (init = -61.1651)
    K2:     1998.16236 +/- 5689.11336 (284.72%) == '0.25*K1'
[[Correlations]] (unreported correlations are < 0.100)
    C(K1, d_HG)    =  1.000
    C(d_HG2, d_HG) = -0.969
    C(K1, d_HG2)   = -0.965
    C(d_HG2, d_H)  =  0.526
    C(d_HG, d_H)   = -0.462
    C(K1, d_H)     = -0.450
```

7.6 DD · A 1:2 Binding Isotherm (Identical Sites) - Repetition 2

```
[[Fit Statistics]]
    # fitting method   = leastsq
    # function evals   = 7
    # data points      = 19
    # variables        = 4
    chi-square         = 1.2759e-04
    reduced chi-square = 8.5059e-06
    Akaike info crit   = -218.311684
    Bayesian info crit = -214.533928
[[Variables]]
    K1:     5002.63574 +/- 18655.8373 (372.92%) (init = 5003.102)
    d_HG2: -61.5058273 +/- 0.04870718 (0.08%) (init = -61.50583)
    d_HG:  -61.4319362 +/- 0.42007286 (0.68%) (init = -61.43193)
    d_H:   -61.2677169 +/- 0.00157116 (0.00%) (init = -61.26772)
    K2:     1250.65893 +/- 4663.95932 (372.92%) == '0.25*K1'
[[Correlations]] (unreported correlations are < 0.100)
    C(K1, d_HG)    =  1.000
    C(d_HG2, d_HG) = -0.993
    C(K1, d_HG2)   = -0.992
    C(d_HG2, d_H)  =  0.453
    C(d_HG, d_H)   = -0.412
    C(K1, d_H)     = -0.406
```

7.7 DD · A 1:2 Binding Isotherm (Independent Sites) - Repetition 1

```
[[Fit Statistics]]
    # fitting method   = leastsq
    # function evals   = 6
    # data points      = 18
    # variables        = 4
    chi-square         = 3.8497e-05
    reduced chi-square = 2.7498e-06
    Akaike info crit   = -226.995257
    Bayesian info crit = -223.433770
[[Variables]]
    K1:     16278.6099 +/- 661.905537 (4.07%) (init = 16278.66)
    d_HG2: -61.5634817 +/- 0.00539244 (0.01%) (init = -61.56348)
    d_HG:  -61.3712798 +/- 0.00193418 (0.00%) (init = -61.37128)
    d_H:   -61.1546022 +/- 0.00122781 (0.00%) (init = -61.1546)
    K2:     898.293902 +/- 36.5255825 (4.07%) == '14622976 / K1'
[[Correlations]] (unreported correlations are < 0.100)
    C(K1, d_HG2)   = -0.911
    C(d_HG2, d_HG) = -0.891
    C(K1, d_HG)    =  0.752
    C(K1, d_H)     =  0.459
    C(d_HG2, d_H)  = -0.293
```

7.8 DD · A 1:2 Binding Isotherm (Independent Sites) - Repetition 2

```
[[Fit Statistics]]
    # fitting method   = leastsq
    # function evals   = 6
    # data points      = 19
    # variables        = 4
    chi-square         = 3.9289e-05
    reduced chi-square = 2.6193e-06
    Akaike info crit   = -240.690892
    Bayesian info crit = -236.913136
[[Variables]]
    K1:     13843.5561 +/- 1139.72768 (8.23%) (init = 13843.78)
    d_HG2: -61.5497001 +/- 0.00919229 (0.01%) (init = -61.5497)
    d_HG:  -61.3738410 +/- 0.00236466 (0.00%) (init = -61.37384)
    d_H:   -61.2645625 +/- 9.7251e-04 (0.00%) (init = -61.26456)
    K2:     1056.30200 +/- 86.9644057 (8.23%) == '14622976 / K1'
[[Correlations]] (unreported correlations are < 0.100)
    C(K1, d_HG2)   = -0.942
    C(d_HG2, d_HG) = -0.902
    C(K1, d_HG)    =  0.798
    C(K1, d_H)     =  0.468
    C(d_HG2, d_H)  = -0.330
```

8 Molecular Modelling

Molecular mechanics calculations were performed in Schrödinger Suite 2016-4 using MacroModel software.[8] Simplified **AD** 2-mers were used, in which the end-capping protecting groups groups and the *iso*-butyl chains on the phosphine oxides were changed to methyl groups in order to reduce the computational cost. All structures were minimised first and the minimised structures were then used as the starting molecular structures for all MacroModel conformational searches. Two independent searches were performed, using MMFFs and OPLS3 as force fields with implicit solvation in chloroform, as implemented in the software.[9] The charges were defined by the force field library and no cut-off was used for non-covalent interaction. A single hydrogen bond was constrained, with distance defined as (1.7 ± 0.5) Å and force constant of 100. Mixed torsional/Large-Scale Low-Mode Sampling was used with Enhanced torsion sampling options, so as to include ester C–O bonds, and 100 steps per rotatable bond. Maximum of 10.000 iterations was performed per sample with redundant conformers eliminated using root mean square deviation (RMSD) of 2 Å. The minima converged on a Polak-Ribiere Conjugate Gradient (PRCG) with a threshold of 1.0.

The resulting lowest energy structures were used as the starting structures for a further conformational search with no constrained interactions. The second search was only performed using OPLS3 force field and the above parameters were changed to a maximum of 20,000 and the structure redundancy criterion was reduced to 2 Å RMSD. The lowest energy conformation was further minimised with OPLS3 force field and the PRCG with a threshold of 0.01.

The results were visualised using CYLview.[10]

References

(1) Klaeui, W.; Song, C. E. *Inorg. Chem.* **1989**, *28*, 3845–3849.

(2) De Rycke, N.; St Denis, J.; Hughes, J.; Rosadiuk, K.; Gleason, J. *Synlett* **2014**, *25*, 2802–2805.

(3) Newville, M.; Stensitzki, T.; Allen, D. B.; Ingargiola, A. LMFIT: Non-Linear Least-Square Minimization and Curve-Fitting for Python., version 0.8.0, 2014.

(4) Oliphant, T. E. A guide to NumPy., 2006.

(5) Perez, F.; Granger, B. E. *Comput. Sci. Eng.* **2007**, *9*, 21–29.

(6) Hunter, J. D. *Comput. Sci. Eng.* **2007**, *9*, 90–95.

(7) Kass, R. E.; Raftery, A. E. *J. Am. Stat. Assoc.* **1995**, *90*, 773–795.

(8) Schrödinger Release 2016-4: MacroModel., New York, NY, 2016.

(9) Harder, E. et al. *J. Chem. Theory Comput.* **2016**, *12*, 281–296.

(10) Legault, C. Y. CYLview, 1.0b., 2009.
